# Non-Abelian gauge field optics

**DOI:** 10.1038/s41467-019-10974-8

**Published:** 2019-07-16

**Authors:** Yuntian Chen, Ruo-Yang Zhang, Zhongfei Xiong, Zhi Hong Hang, Jensen Li, Jian Qi Shen, C. T. Chan

**Affiliations:** 1 School of Optical and Electronic Information, Huazhong University of Science and Technology, 430074 Wuhan, China; 2 Wuhan National Laboratory of Optoelectronics, Huazhong University of Science and Technology, 430074 Wuhan, China; 3 Department of Physics, The Hong Kong University of Science and Technology, Clear Water Bay, Hong Kong; 4 School of Physical Science and Technology and Institute for Advanced Study, Soochow University, 215006 Suzhou, China; 5 Centre for Optical and Electromagnetic Research, State Key Laboratory of Modern Optical Instrumentations, Zhejiang University, 310058 Hangzhou, China

**Keywords:** Metamaterials, Transformation optics

## Abstract

The concept of gauge field is a cornerstone of modern physics and the synthetic gauge field has emerged as a new way to manipulate particles in many disciplines. In optics, several schemes of Abelian synthetic gauge fields have been proposed. Here, we introduce a new platform for realizing synthetic SU(2) non-Abelian gauge fields acting on two-dimensional optical waves in a wide class of anisotropic materials and discover novel phenomena. We show that a virtual non-Abelian Lorentz force arising from material anisotropy can induce light beams to travel along Zitterbewegung trajectories even in homogeneous media. We further design an optical non-Abelian Aharonov–Bohm system which results in the exotic spin density interference effect. We can extract the Wilson loop of an arbitrary closed optical path from a series of gauge fixed points in the interference fringes. Our scheme offers a new route to study SU(2) gauge field related physics using optics.

## Abstract (web summary)

In optics, schemes have been proposed to realize synthetic gauge fields, but are restricted to the Abelian type. Here, the authors demonstrate synthetic SU(2) non-Abelian gauge fields in anisotropic media, which allows the study of novel optical phenomena not found in Abelian synthetic gauge field systems.

## Introduction

Gauge fields originated from classical electromagnetism, and have become the kernel of fundamental physics after being extended to non-Abelian by Yang and Mills^1^. Apart from real gauge bosons, emergent gauge fields in either real^2^ or parameter spaces^3,4^ have recently been widely used to elucidate the complicated dynamics in a variety of physical systems^5^, including electronic^6,7^, ultracold atom^8–10^, and photonic^11–31^ systems. The geometric nature^32^ of gauge theory makes it a powerful tool for studying the topological phases of matter^33–36^.

The concept of emergent gauge fields has offered us new insights in optics and photonics, such as the manifestation of the gauge structure (Berry connection and curvature) in momentum space^11–16^. Artificial gauge fields realized by breaking time reversal symmetry with magnetic effects^17–19^ or dynamic modulation^21–23^ have given rise to new paradigms for controlling light trajectories in real space. Even for time-reversal-invariant systems, a pair of virtual magnetic fields—each being the time-reversed partner of the other—can be generated using methods, such as coupled optical resonators^20^, engineering lattices with strain^24,25^, or reciprocal metamaterials^26–30^. However, except for a few works revealing the non-Abelian gauge structure in momentum space^13,14,16^, all of these schemes of synthetic gauge fields in real space are restricted to the Abelian type.

Recently, anisotropic metamaterials were used to manipulate light through artificial Abelian gauge fields^27–30^. It was demonstrated that the off-diagonal components of permittivity and permeability appear as a pair of “spin-dependent” vector potentials in the two-dimensional (2D) wave equation for certain anisotropic media. Though the material parameters are subjected to strong restriction in this scheme, the internal pseudo-spin degree of freedom implies the possible generalization to a synthetic non-Abelian gauge field theory for light by coupling the spin-up and spin-down states.

In this work, we discover that the transport of optical waves in a wide class of anisotropic media can be associated with an emergent 2D non-Abelian SU(2) gauge interaction in real space, enabling us to obtain the first scheme for realizing synthetic non-Abelian gauge field for classical waves. Contrary to intuition, we show that a more exotic general SU(2) gauge framework can manifest in 2D optical dynamics, provided the restriction on the material parameters employed in refs. ^27–30^ is relaxed. Our platform presents broader applicability and allows the study of novel optical phenomena not found in Abelian synthetic gauge field systems. We illustrate our idea with two examples. The first example is the Zitterbewegung (ZB) of light in homogeneous non-Abelian media, which refers to the trembling motion of wave packets^37^. ZB has been realized in systems possessing Dirac dispersion^38–43^, but we will see that ZB of light can arise from a distinctly different mechanism: emergent non-Abelian Lorentz force. In the second example, we propose for the first time a concrete design of a genuine non-Abelian Aharonov–Bohm (AB) system^44^ using two synthetic non-Abelian vortices, and reveal that the noncommutativity of winding around the two vortices gives rise to nontrivial interference results. In particular, we show that there exists a series of fixed points in the interference fringes invariant under gauge transformation, from which we can obtain the Wilson loops of the closed path concatenated by the two interfering optical paths. As evidenced by the examples, our scheme offers a fresh angle to understand the dynamic effects of light in anisotropic media, and also suggests an optical approach to probe new physics accompanied by SU(2) gauge fields.

## Results

### Non-Abelian gauge fields acting on light

Our scheme focuses on 2D propagating optical waves in nondissipative anisotropic media characterized by the permittivity and permeability tensors:1

Here, all of the parameters depend on *x*, *y*; the diagonal blocks $$\mathord{\buildrel{\lower3pt\hbox{$\scriptscriptstyle \leftrightarrow$}} \\ \over \varepsilon } _T$$, $$\mathord{\buildrel{\lower3pt\hbox{$\scriptscriptstyle \leftrightarrow$}} \\ \over \mu } _T$$, $$\varepsilon _z$$, $$\mu _z$$ are real numbers, while the off-block-diagonal components $${\mathbf{g}}_i = (g_{i{\kern 1pt} x},g_{i{\kern 1pt} y})^ \top = g_{i{\kern 1pt} x}{\mathbf{e}}_x + g_{i{\kern 1pt} y}{\mathbf{e}}_y$$ (*i* = 1, 2) are in-plane complex vectors whose imaginary parts could be induced by the gyrotropic effect with in-plane gyration vectors. The only constraint on the media is the “in-plane duality”, $$\mathord{\buildrel{\lower3pt\hbox{$\scriptscriptstyle \leftrightarrow$}} \\ \over \varepsilon } _T = \alpha \mathord{\buildrel{\lower3pt\hbox{$\scriptscriptstyle \leftrightarrow$}} \\ \over \mu } _T$$, where *α* is a positive constant. For simplicity, we set *α* = 1 in the following, and *α* ≠ 1 results can be obtained directly by redefining $$\varepsilon _0 \to \alpha {\kern 1pt} \varepsilon _0$$. Under this constraint, the in-plane monochromatic wave equation of frequency *ω* can be written as

2 $${\hat {H}}\left| \psi \right\rangle = \left[ {\frac{1}{2}(\hat {\mathbf{p}} - {{\hat {\cal{A}}}})\, \cdot \,{ \mathop{\mathbf{m}}\limits^\leftrightarrow} ^{ - 1}\, \cdot \,(\hat {\mathbf{p}} - {{\hat {\cal{A}}}}) - {\hat {\cal{A}}}_0 + V_0} \right]\left| \psi \right\rangle = 0.$$

Here $$\left| \psi \right\rangle = (E_z,\eta _0H_z)^ \top$$ $$\left( {\eta _0 = \sqrt {\mu _0/\varepsilon _0} } \right)$$ serves as a two-component wave function, and $$\hat H$$ resembles the Hamiltonian of a non-relativistic spin-1/2 particle traveling in SU(2) non-Abelian gauge potentials^45^, where $$\widehat {\mathbf{p}} = - {\mathrm{i}}\, \hat \sigma_0\partial _i{\mathbf{e}}^i$$ (*i* = 1, 2) is the canonical momentum operator with $$\hat \sigma _0$$ being the 2D identity matrix, $${\kern 1pt} \mathop{\mathbf{m}}\limits^{\leftrightarrow} = \mathord{\buildrel{\lower3pt\hbox{$\scriptscriptstyle \leftrightarrow$}} \\ \over \varepsilon } _T^{ - 1}{\mathrm{det}}({\kern 1pt} \mathord{\buildrel{\lower3pt\hbox{$\scriptscriptstyle \leftrightarrow$}} \\ \over \varepsilon } _T)/2$$ represents an effective anisotropic mass, in particular, $${\hat {\cal{A}}} = {\cal{A}}^1\hat \sigma _1 + {\cal{A}}^2\hat \sigma _2$$ and $${\hat {\cal{A}}}_0 = {\cal{A}}_0^a\hat \sigma _a$$ ($$\hat \sigma _a$$ (*a* = 1, 2, 3) are Pauli matrices) can be interpreted as emergent non-Abelian vector and scalar potentials, respectively, and *V*_0_ is an additional Abelian scalar potential. As shown in Table 1, the emergent gauge potentials are determined by the material parameters, especially, the vector potential directly corresponds to the off-diagonal terms of $$\mathord{\buildrel{\lower3pt\hbox{$\scriptscriptstyle \leftrightarrow$}} \\ \over \varepsilon }$$ and $$\mathord{\buildrel{\lower3pt\hbox{$\scriptscriptstyle \leftrightarrow$}} \\ \over \mu }$$. This correspondence can be intuitively understood from the SU(2) gauge covariance of 2D Maxwell equations (see the “Methods” section), and the detailed derivation of Eq. (2) is given in the Supplementary Note 1. Thereby, in this broad class of anisotropic media, the materials’ influence on the 2D optical waves imitates a SU(2) gauge interaction. Furthermore, if the background media are extended to be bi-anisotropic materials, a complete construction of $${\mathrm{U}}(2) = {\mathrm{SU}}(2)\rtimes {\mathrm{U}}(1)$$ gauge fields for light can be achieved (see Supplementary Note 1).

**Table 1:** The expressions of the synthetic SU(2) and U(1) gauge potentials in terms of the constitutive parameters of the non-Abelian media

SU(2)	Vector potential $\hat {\cal{A}} = {\cal{A}}^a\hat \sigma _a$	${\cal{A}}^1 = k_0{\mathrm{Re}}\left( {{\mathbf{g}}_ - } \right) \times {\mathbf{e}}_z$ ^a^
		$${\cal{A}}^2 = k_0{\mathrm{Im}}\left( {{\mathbf{g}}_ - } \right) \times {\mathbf{e}}_z$$
		$${\cal{A}}^3 = 0$$
	Scalar potential $$\hat {\cal{A}}_0 = {\cal{A}}_0^a\hat \sigma _a$$	$${\cal{A}}_0^1 = k_0{\mathbf{e}}_z \cdot \left[ {\nabla \times \left( {\mathord{\buildrel{\lower3pt\hbox{$\scriptscriptstyle \leftrightarrow$}} \\ \over \varepsilon } _T^{ - 1} \cdot {\mathrm{Im}}\left( {{\mathbf{g}}_ + } \right)} \right)} \right]$$
		$${\cal{A}}_0^2 = - k_0\,{\mathbf{e}}_z \cdot \left[ {\nabla \times \left( {\mathord{\buildrel{\lower3pt\hbox{$\scriptscriptstyle \leftrightarrow$}} \\ \over \varepsilon } _T^{ - 1} \cdot {\mathrm{Re}}\left( {{\mathbf{g}}_ + } \right)} \right)} \right]$$
		$${\cal{A}}_0^3 = k_0^2\left[ {\frac{{\varepsilon _z - \mu _z}}{2} - 2\,{\mathrm{Re}}\left( {{\mathbf{g}}_ - ^\dagger \cdot {\kern 1pt} \mathord{\buildrel{\lower3pt\hbox{$\scriptscriptstyle \leftrightarrow$}} \\ \over \varepsilon } _T^{ - 1} \cdot {\mathbf{g}}_ + } \right)} \right]$$
U(1)	Scalar potential	$$V_0 = k_0^2\left[ {\left( {{\mathbf{g}}_ + ^\dagger \cdot \mathord{\buildrel{\lower3pt\hbox{$\scriptscriptstyle \leftrightarrow$}} \\ \over \varepsilon } _T^{ - 1} \cdot {\mathbf{g}}_ + } \right) - \frac{{\varepsilon _z + \mu _z}}{2}} \right]$$

^a^Here $k_0 = \omega /c$ is the vacuum wave number, and ${\mathbf{g}}_ \pm = ({\mathbf{g}}_1 \pm {\mathbf{g}}_2^ \ast )/2$.

The emergent SU(2) gauge potential $\{ {\hat {\cal{A}}}_\mu \} = \{ {\hat {\cal{A}}}_0,{\hat {\cal{A}}}\}$ induces a synthetic SU(2) gauge field acting on light:

3 $$\hat {\cal{F}}_{\mu \nu } = {\mathrm{i}}[\hat {\cal{D}}_\mu ,\hat {\cal{D}}_\nu ] = \partial _\mu {\hat {\cal{A}}}_\nu - \partial _\nu {\hat {\cal{A}}}_\mu - {\mathrm{i}}[{\hat {\cal{A}}}_\mu ,{\hat {\cal{A}}}_\nu ],$$

where $\hat {\cal{D}}_\mu = \hat \sigma _0\partial _\mu - {\mathrm{i}}{\kern 1pt} {\hat {\cal{A}}}_\mu$ (*μ* = 0, 1, 2) is the covariant derivative. Analogous to real electromagnetic (EM) fields, the synthetic SU(2) gauge field can be separated into a non-Abelian magnetic field $\hat {\cal{B}} = \frac{1}{2}\epsilon ^{ij}\hat {\cal{F}}_{ij}{\mathbf{e}}_z$ along the *z*-axis and a non-Abelian in-plane electric field $\hat {\cal{E}} = - \hat {\cal{F}}_{0i}{\mathbf{e}}_i$, which are associated with the gauge potential as

4 $$\hat {\cal{B}} = \nabla \times {\hat {\cal{A}}} - {\mathrm{i}}{\hat {\cal{A}}} \times {\hat {\cal{A}}},\quad \hat {\cal{E}} = \nabla {\hat {\cal{A}}}_0 + {\mathrm{i}}[{{\hat {\cal{A}}}}_0,{{\hat {\cal{A}}}}].$$

The second terms of $\hat {\cal{B}}$, $\hat {\cal{E}}$ cannot be found in the Abelian case since they are induced entirely by the noncommutativity of the non-Abelian gauge potential. Indeed, a matrix-valued gauge potential would not be regarded as (apparently) non-Abelian, unless some of its components do not commute with each other $[{\hat {\cal{A}}}_\mu ,{\hat {\cal{A}}}_\nu ] \ne 0$^10^. For instance, the scheme in ref. ^27^ is actually a specific reduction of ours with the strict constraints on the media that (i) ${\mathbf{g}}_1 = - {\mathbf{g}}_2$ being real and (ii) $\varepsilon _z = \mu _z$. In this case, the vector potential only has $\hat \sigma _1$ component ${{\hat {\cal{A}}}} = {\cal{A}}^1{\hat {\sigma}}_1$ and the scalar potential ${\hat {\cal{A}}}_0$ vanishes. As such, $[{{\hat {\cal{A}}}}_i,{{\hat {\cal{A}}}}_j] \equiv 0$, and the gauge group is reduced to the Abelian subgroup ${\mathrm{U}}(1)$ of ${\mathrm{SU}}(2)$. In general, if Eq. (2) has any U(1) spin rotation symmetry, which means $\hat U\hat H\hat U^\dagger = \hat H$ for $\hat U = {\mathrm{exp}}\left( {{\mathrm{i}}\phi {\kern 1pt} \vec n \cdot \vec {\hat \sigma } } \right)$ with a parameter *ϕ*, the gauge potential would be reducible. Hence, only for those materials that can imitate irreducible SU(2) gauge potentials, we call them non-Abelian media.

The two-component wave function of light $|\psi \rangle$ behaves like a spin-1/2 spinor with the pseudo-spin at a local point

5 $$\vec s = \langle \psi |\vec {\hat \sigma } |\psi \rangle /|\psi |^2,$$

where the overhead arrow indicates a vector in the pseudo-spin space, and $\langle \psi |\vec {\hat \sigma } |\psi \rangle$ gives the local spin density. The frame $\{ \vec e_a\}$ in the pseudo-spin space can be chosen arbitrarily. The rotation of the frame corresponds to a gauge transformation of spinor $|\psi^{\prime}\rangle = \hat U({\mathbf{r}})|\psi \rangle$, where in general $\hat U({\mathbf{r}})$ is a space-varying SU(2) matrix. By substituting $|\psi^{\prime}\rangle$ into Eq. (2), one can easily check that the wave equation is gauge covariant as long as the material is transformed accordingly (see Supplementary Note 2), while the synthetic gauge potentials and fields obey the gauge transformations

6 $${{\hat {\cal{A}}}}^{\prime}_\mu = \hat U{{\hat {\cal{A}}}}_{\mu} \hat U^\dagger + {\mathrm{i}}\hat U\partial _\mu \hat U^\dagger ,$$

7 $$\hat {\cal{B}}^\prime = \hat U\hat {\cal{B}}\hat U^\dagger ,\quad \hat {\cal{E}}^\prime = \hat U\hat {\cal{E}}\hat U^\dagger .$$

In addition, it is worth comparing the present idea of non-Abelian gauge field optics (NAGFO) with the transformation optics (TO)^46–50^. When TO is applied to design invisibility cloaks, it results in anisotropic media whose permittivity and permeability are real and equal $$\mathord{\buildrel{\lower3pt\hbox{$\scriptscriptstyle \leftrightarrow$}} \\ \over \varepsilon } = \mathord{\buildrel{\lower3pt\hbox{$\scriptscriptstyle \leftrightarrow$}} \\ \over \mu }$$^46,47^. Due to the equivalence of the constitutive tensor and the metric of a curved spacetime for light, such kind of duality symmetric materials can also be used to mimic gravitational effects^49–53^. In contrast to TO, NAGFO involves a more general class of complex-valued media respecting in-plane duality symmetry. The in-plane block $$\mathord{\buildrel{\lower3pt\hbox{$\scriptscriptstyle \leftrightarrow$}} \\ \over \varepsilon } _T$$ of permittivity, which determines the effective mass in Eq. (2), can alternatively be equated to the metric of a virtual 2D curved space as with TO, whereas, apart from $$\mathord{\buildrel{\lower3pt\hbox{$\scriptscriptstyle \leftrightarrow$}} \\ \over \varepsilon } _T$$, all the remaining components of $$\mathord{\buildrel{\lower3pt\hbox{$\scriptscriptstyle \leftrightarrow$}} \\ \over \varepsilon }$$ and $$\mathord{\buildrel{\lower3pt\hbox{$\scriptscriptstyle \leftrightarrow$}} \\ \over \mu }$$ contribute to the synthetic SU(2) gauge potentials. Therefore, NAGFO proposes an optical way to simulate the 2D spinor systems under both a SU(2) gauge interaction and the influence of a curved space. To highlight the effects stemming purely from the non-Abelian gauge interaction, we will hereinafter concentrate on the simplified scenario that $$\mathord{\buildrel{\lower3pt\hbox{$\scriptscriptstyle \leftrightarrow$}} \\ \over \varepsilon } _T = \varepsilon _T{ \mathop{\mathbf{I}}\limits^\leftrightarrow}{\kern 1pt} _{2 \times 2}$$ is isotropic and homogeneous. As such, the virtual 2D background space is trivialized to be flat, and the effective mass is reduced to $$m = \varepsilon _T/2$$.

### Zitterbewegung of optical beams

The wave packet dynamics in homogeneous media can give the most straightforward effect distinguishing the non-Abelian media from the Abelian type. The effective Abelian electric and magnetic fields vanish in homogeneous media^27^, whereas the non-Abelian fields persist due to the noncommutativity of ${{\hat {\cal{A}}}}_{\mu}$. In our case, $\hat {\cal{B}} = {\cal{B}}\hat \sigma _3$ with ${\cal{B}} = {\mathrm{i}}k_0^2({\mathbf{g}}_ - \times {\mathbf{g}}_ - ^ \ast )$, and $\hat {\cal{E}} = 2{\cal{A}}_0^3\left( {{\cal{A}}^2\hat \sigma _1 - {\cal{A}}^1\hat \sigma _2} \right)$. We consider the propagation of 2D optical beams in homogeneous non-Abelian media. In general, there are two non-degenerate branches of plane wave eigenstates. Because the two eigenstates of a certain direction of wave vector **k** are always orthogonal, their pseudo-spins correspond to a pair of antipodal points on the Bloch sphere. Generally speaking, the non-degenerate eigenmodes would evolves independently along different semiclassical trajectories. However, if the two eigenstates for a particular direction of **k** are quasi-degenerate, in the overlapped region, their superposed wave can be viewed as an intact “semiclassical particle” with an internal spin degree of freedom, whose centroid trajectory follows the Hamilton’s canonical equations (see the “Methods” section)

8 $$\frac{{\mathrm{{d}}}}{{{\mathrm{{d}}}\tau }}\langle \widehat {\mathbf{p}}\rangle = {\mathrm{i}}\left\langle {[\hat H,\widehat {\mathbf{p}}]} \right\rangle \equiv 0\quad \Rightarrow \quad \langle {\hat{\mathbf{p}}}\rangle \equiv {\mathbf{k}},$$

9 $$\frac{{\mathrm{{d}}}}{{{\mathrm{{d}}}\tau }}\langle \widehat {\mathbf{r}}\rangle = \langle \widehat {\mathbf{v}}\rangle = \frac{1}{m}({\mathbf{k}} - {\cal{A}}^a\langle \hat \sigma _a\rangle ).$$

Here $\widehat {\mathbf{v}} = \frac{{\mathrm{{d}}}}{{{\mathrm{{d}}}\tau }}{\hat{\mathbf{r}}} = {\mathrm{i}}[\hat H,{\hat{\mathbf{r}}}] = ({\hat{\mathbf{p}}} - {{\hat {\cal{A}}}})/m$ is the velocity operator, $\tau$ represents path parameter along the beam, and $\langle \hat a\rangle ({\mathbf{r}}_0) = {\int} {\mathrm{{d}}}{\mathbf{r}}_ \bot {\kern 1pt} \psi ^\dagger ({\mathbf{r}}_0 + {\mathbf{r}}_ \bot )\hat a({\mathbf{r}}_0 + {\mathbf{r}}_ \bot ){\kern 1pt} \psi ({\mathbf{r}}_0 + {\mathbf{r}}_ \bot )$ means the expectation value of an operator $\hat a$ averaged over the transverse cross section of a point ${\mathbf{r}}_0$ along an optical beam, differing from the local expectation value $\langle \psi |\hat a|\psi \rangle ({\mathbf{r}}) = \psi ^\dagger ({\mathbf{r}})\hat a({\mathbf{r}})\psi ({\mathbf{r}})$. According to Eq. (8), the canonical momentum along the beam is conserved, and is equal to the quasi-degenerate eigen wave vector **k** (see Eq. (39)). Moreover, it turns out that the in-plane projection of the total energy flux over the transverse cross section of the beam is always parallel to the velocity given by Eq. (9) (see proof in the section “Methods”), therefore the canonical equations do describe the path of energy propagation. Along the optical beam, the pseudo-spin $\vec s = \langle \vec {\hat \sigma } \rangle$ undergoes precession as follows:

10 $$\frac{{\mathrm{{d}}}}{{{\mathrm{{d}}}\tau }}\vec s = {\mathrm{i}}\langle [\hat H,\vec{\hat{\sigma}}\, ]\rangle = \vec \Omega \times \vec s,$$

where $\vec \Omega = - 2\left( {{\cal{A}}_0^a + \frac{1}{m}{\mathbf{k}} \cdot {\cal{A}}^a} \right)\vec e_a$ is the precession angular velocity. During precession, the pseudo-spin component parallel to $\vec \Omega$ is conserved.

In terms of Eqs. (8–10), we arrive at the Newton-type equation of motion where a virtual non-Abelian Lorentz force^10,45^ associated with the non-Abelian fields emerges

11 $$\begin{array}{*{20}{l}} {m\frac{{{\mathrm{{d}}}^2}}{{{\mathrm{{d}}}\tau ^2}}\langle \hat {\mathbf{r}}\rangle } \hfill & = \hfill & {\frac{1}{2}\langle \hat {\mathbf{v}} \times \hat {\cal{B}} + \hat {\cal{B}} \times \hat {\mathbf{v}}\rangle + \langle \hat {\cal{E}}\rangle } \hfill \\ {} \hfill & = \hfill & {\langle \hat {\mathbf{j}}_{\hat \sigma _3}\rangle \times {\cal{B}} + {\cal{E}}^a\langle \hat \sigma _a\rangle ,} \hfill \end{array}$$

Here, $\widehat {\mathbf{j}}_{\hat \sigma _3} = \frac{1}{2}\left( {\widehat {\mathbf{v}}\hat \sigma _3 + \hat \sigma _3\widehat {\mathbf{v}}} \right) = \frac{1}{m}\widehat {\mathbf{p}}\hat \sigma _3$ represents the $\hat \sigma _3$-component of the linear spin current operator^54^, thus the non-Abelian Lorentz force can also be regarded as a spin-induced force with a magnetic part acting on the spin current and an electric part acting on the average spin over the transverse cross sections of the beam. In particular, the magnetic part of the force, ${\mathbf{f}}_{\hat \sigma _3} = \langle \widehat {\mathbf{j}}_{\hat \sigma _3}\rangle \times {\cal{B}}$, duplicates the “spin transverse force” in electronics which acts on an electronic spin current exerted by a vertical electric field^54^.

The integration of either the canonical equations or Eq. (11) yields the intensity centroid trajectory of the beam

12 $$\begin{array}{*{20}{l}} {\langle \widehat {\mathbf{r}}\rangle } \hfill & = \hfill & {\frac{1}{m}\left[ {{\mathbf{k}} - {\cal{A}}^a{s_0}_a + \frac{1}{{{\mathrm{\Omega }}^2}}{\mathbf{F}}^a\epsilon _{abc}\Omega ^b{s_0}^c} \right]\tau } \hfill \\ {} \hfill & {} \hfill & { - \frac{{{\mathbf{F}}^a}}{{m{\mathrm{\Omega }}^2}}\left[ {({\mathrm{cos}}(\tau {\mathrm{\Omega }}) - 1)\delta _{ac} + \frac{{{\mathrm{sin}}(\tau {\mathrm{\Omega }})}}{{\mathrm{\Omega }}}\epsilon _{abc}{\mathrm{\Omega }}^b} \right]{s_0}^c,} \hfill \end{array}$$

where ${\mathbf{F}}^a = (\vec \Omega \times {\hat {\cal{A}}})^a = {\cal{E}}^a + {\mathbf{k}} \times {\cal{B}}^a/m$, $\Omega = |\vec \Omega |$, $\vec s_0$ represents the initial spin, $\epsilon _{abc}$ is the Levi–Civita symbol, and the initial position of the beam is assumed at $\langle \widehat {\mathbf{r}}\rangle _0 = 0$. The first line of the equation refers to a straight path, while the second line shows that the beam oscillates around the equilibrium path periodically. As a result, the emergent non-Abelian Lorentz force may lead to wavy trajectories for optical beams propagating in the non-Abelian media. This phenomenon resembles the ZB effect of Dirac particles^37^. According to Eq. (12), the trembling motion of light depends not only on the non-Abelian gauge fields but also on the initial spin $\vec s_0$ of the beam. If the initial state is purely one of the eigenmodes with the wave vector in **k** direction, i.e., $\vec s_0$ is along $\vec \Omega ({\mathbf{k}})$, the trembling term in Eq. (12) will vanish. This implies the present ZB effect stems from the interference of the two quasi-degenerate eigenmodes just as electronic ZB is caused by the superposition of positive and negative energy components (see Supplementary Note 3).

In recent years, ZB has been investigated for spin–orbit coupled atoms^38,39^ and photons^40–43^. However, unlike most schemes of ZB for light realized in periodic systems^40–42^, our result shows that light can travel along curved paths even if the background medium is homogeneous. At first glance, this counterintuitive curved trajectory seems to violate the momentum conservation in translation invariant systems. However, it is well known that the kinetic momentum associated with centroid movement can be different from the canonical momentum for a particle traveling in a background vector potential. This conclusion is also valid for our situation. As shown in Eqs. (8) and (9), the semiclassical canonical momentum $\langle \widehat {\mathbf{p}}\rangle$ is always conserved in homogeneous media, while the kinetic momentum $m\langle \widehat {\mathbf{v}}\rangle$ deviates from $\langle \widehat {\mathbf{p}}\rangle$ and can change along the path by virtue of the synthetic non-Abelian potential ${\hat {\cal{A}}}$. A more rigorous analysis shows that the conserved quantity protected by space translational symmetry in generic non-Abelian media is the time-averaged Minkowski-type momentum ${\int} {\mathrm{{d}}}^3x\,{\mathrm{Re}}\left( {{\mathbf{D}}^ \ast \times {\mathbf{B}}} \right)$, while the centroid motion corresponds to the Abraham-type momentum ${\int} {\mathrm{{d}}}^3x\,{\mathrm{Re}}\left( {{\mathbf{E}}^ \ast \times {\mathbf{H}}} \right)/c^2$.

### Example I: ZB induced by non-Abelian magnetic field

**Fig. 1:**
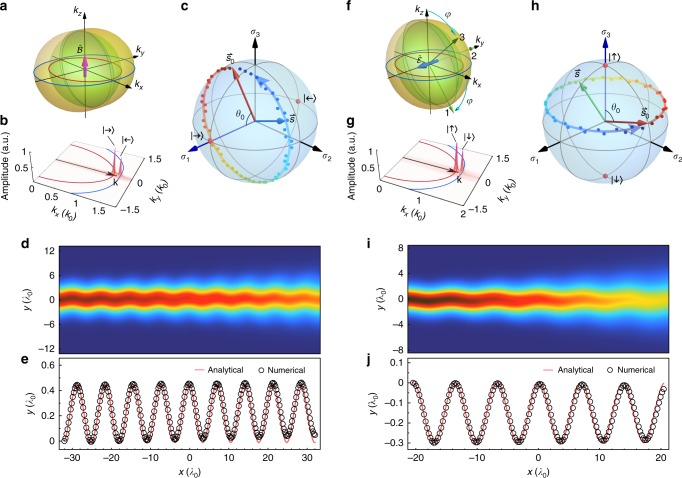
Zitterbewegung effect in homogeneous non-Abelian media. **a**–**e** ZB induced by a synthetic non-Abelian magnetic field in a gyrotropic medium with the parameters $$\mathord{\buildrel{\lower3pt\hbox{$\scriptscriptstyle \leftrightarrow$}} \\ \over \varepsilon }_{T} = \mathord{\buildrel{\lower3pt\hbox{$\scriptscriptstyle \leftrightarrow$}} \\ \over \mu }_{T} = 1.5{ \mathop{\mathbf{I}}\limits^\leftrightarrow}{\kern 1pt}_{2 \times 2}$$, $$\varepsilon _{z} = \mu _{z} = 1.5$$, $${\mathbf{g}}_1 = - {\mathbf{g}}_2^ \ast = (0.3{\mathrm{i}}, - 0.07)^ \top$$. This medium produces a synthetic SU(2) magnetic field along $$z$$ direction, $$\hat {\cal{B}} = - k_0^2{\kern 1pt} 0.042{\mathbf{e}}_z\hat \sigma _3$$, with a null SU(2) electric field $$\hat {\cal{E}} = 0$$. **f**–**j** ZB induced by a synthetic non-Abelian electric field in a biaxial non-magnetic medium with the parameters $$\varepsilon _1 = 1.65$$, $$\varepsilon _2 = 2.45$$, $$\varepsilon _3 = 3$$, and $$\mu /\mu _0 = 1$$. The synthetic $${\mathrm{SU}}(2)$$ electric field, $$\hat {\cal{E}} = - k_0^3{\kern 1pt} 0.08919\,{\mathbf{e}}_y\hat \sigma _2$$, is along the *y*-aixs, while the SU(2) magnetic field vanishes $$\hat {\cal{B}} = 0$$. **a**, **f** The isofrequency surfaces and their *xy* cross sections (red and blue curves) of both cases. The green arrows in **f** are the three principal axes 1, 2, 3 of permittivity tensor. **b**, **g** Fourier spectra in *k*-space of the beams in the two media. In each case, the two peaks in the spectrum correspond to the two eigenmodes with wave vectors in the *x* direction. And the average wave vectors **k** are marked by the black arrows. **c**, **h** The spin precession along each beam on the Bloch sphere. The colored dots are the numerical data within one ZB period. **d**, **i** Full-wave simulated intensity distributions, where the beam waists equal $$4.4\lambda _0$$ and $$6.2\lambda _0$$, respectively ($$\lambda _0 = 2\pi /k_0$$ is the wavelength in vacuum). **e**, **j** Numerical (black circles) and analytical (red curves) trajectories of the intensity centroid

According to the theory, the ZB effect for monochromatic beams can be generated by either non-Abelian magnetic fields or non-Abelian electric fields. In Fig. 1a–e, we first show an example of ZB induced solely by a non-Abelian magnetic field. To realize nonzero $\hat {\cal{B}}$ but vanishing $\hat {\cal{E}}$, we let the medium satisfy $\varepsilon _z = \mu _z$, ${\mathbf{g}}_1 = - {\mathbf{g}}_2^ \ast = ( - {\mathrm{i}}{\cal{A}}_y^2/k_0,{\cal{A}}_x^1/k_0)^ \top$, then the synthetic SU(2) magnetic field in this medium is given by $\hat {\cal{B}} = 2{\cal{A}}_x^1{\cal{A}}_y^2{\mathbf{e}}_z\hat \sigma _3$. The isofrequency surfaces of eigenmodes are illustrated in Fig. 1a. Along the *k*_*x*_ direction, the two eigenstates are $| \mathord{\to} \rangle = (1,1)^ \top /\sqrt 2$ and $| \mathord{\leftarrow} \rangle = (1, - 1)^ \top /\sqrt 2$ with the wave vectors ${\mathbf{k}}_ \pm = \left[ {\sqrt {k_0^2\varepsilon _T\varepsilon _z - ({\cal{A}}_y^2)^2} \pm {\cal{A}}_x^1} \right]{\mathbf{e}}_x$, and their pseudo-spins are polarized along the $\hat \sigma _1$-axis, as labeled on the Bloch sphere in Fig. 1c. As long as $|{\cal{A}}_x^1| \ll k = \sqrt {k_0^2\varepsilon _T\varepsilon _z - ({\cal{A}}_y^2)^2}$, the quasi-degenerate approximation is valid for beams incident from *x* direction. In this case, the precession angular velocity is $\vec \Omega = - 4k{\cal{A}}_x^1/\varepsilon _T\vec e_1$, so the pseudo-spin will precess about the $\hat \sigma _1$-axis. For the initial spin $\vec s_0 = ({\mathrm{cos}}\theta _0,{\mathrm{sin}}\theta _0{\mathrm{cos}}\phi _0,{\mathrm{sin}}\theta _0{\mathrm{sin}}\phi _0)^ \top$ at an angle $\theta _0$ from $\hat \sigma _1$-axis, we can obtain the centroid trajectory of the beam by eliminating the ray parameter *τ* in Eq. (12),

13 $$y(x) = Y_{{\mathrm{ZB}}}\left[ {{\mathrm{sin}}(k_{\mathrm{ZB}}(x - x_0) - \phi _0) + {\mathrm{sin}}\phi _0} \right],$$

where *x*, *y* are the coordinates of centroid. The ZB amplitude

14 $$Y_{{\mathrm{ZB}}} = \frac{{ - {\cal{A}}_y^2{\kern 1pt} {\mathrm{{sin}}}\theta _0}}{{2{\kern 1pt} k{\cal{A}}_x^1}} = \frac{{ - {\cal{A}}_y^2{\kern 1pt} {\mathrm{{sin}}}\theta _0}}{{2{\cal{A}}_x^1\sqrt {k_0^2\varepsilon _T\varepsilon _z - ({\cal{A}}_y^2)^2} }}$$

is proportional to ${\mathrm{{sin}}}\theta _0$, so ZB reaches the maximum when the initial spin $\vec s_0$ is perpendicular to $\vec \Omega$, corresponding to the equal-weighted superposition of the two eigenmodes. Meanwhile, the ZB wave number

15 $$k_{{\mathrm{ZB}}} = \frac{{2k{\cal{A}}_x^1}}{{k - {\cal{A}}_x^1{\mathrm{cos}}\theta _0}} \approx 2{\cal{A}}_x^1 = k_ + - k_ -$$

is equal to the difference of the two eigen wave vectors, showing that ZB originates from the beating between the two eigenstates. Yet we should emphasize the phase beating is not a sufficient condition to realize ZB, and the ZB amplitude cannot be obtained without the knowledge of the non-Abelian dynamics. For instance, if ${\cal{A}}_y^2 = 0$ in the present medium, the beat of the two states persists, however, as the medium is relegated to the Abelian-type with $\hat {\cal{B}} = 0$, ZB just disappears.

We have performed the full-wave simulation of a Gaussian beam propagating in this medium using COMSOL Multiphysics. The beam is emitted along *x*-direction and the angle between its initial spin and $\hat \sigma _1$-axis is set as $\theta _0 = 0.43\pi$. Figure 1b shows the *k*-space Fourier amplitude of the simulated wave function *ψ*, the two peaks in the spectrum manifest that the beam is mainly comprised of the two eigenstates |→〉 and $|{\leftarrow} {\rangle}$. The numerical time-averaged energy densities plotted in Fig. 1d show clearly a transverse tremor along the beam. As shown in Fig. 1e, the centroid trajectory extracted from the full-wave result agrees perfectly with the analytical expression in Eq. (13). And according to the numerical data of the pseudo-spins in one ZB period shown in Fig. 1c, the spin precession about the $\hat \sigma _1$-axis is also demonstrated.

### Example II: ZB induced by non-Abelian electric field

In the previous example, the non-Abelian medium contains both gyroelectric and gyromagnetic components. In fact, the synthetic non-Abelian gauge fields as well as ZB can be simply realized with reciprocal media without gyrotropy. Here, we elaborate on synthesizing non-Abelian electric field with a biaxial non-magnetic material and the ZB effect in it.

We consider a non-magnetic material with the biaxial permittivity $${\kern 1pt} \widetilde {\mathord{\buildrel{\lower3pt\hbox{$\scriptscriptstyle \leftrightarrow$}} \\ \over \varepsilon } }/\varepsilon _0 = {\mathrm{diag}}(\varepsilon _1,\varepsilon _2,\varepsilon _3)$$ $$\left( {\varepsilon _1 < \varepsilon _2 < \varepsilon _3} \right)$$ along the principal axis and the permeability $$\mu /\mu _0 = 1$$. If the second principal axis of $$\mathord{\buildrel{\lower3pt\hbox{$\scriptscriptstyle \leftrightarrow$}} \\ \over \varepsilon }$$ is fixed along the *y*-axis, while the first principal axis is rotated by an angle *φ* with respect to the *x*-axis such that $${\mathrm{{cos}}}^2\varphi\ \varepsilon _1 + {\mathrm{{sin}}}^2\varphi\ \varepsilon _3 = \varepsilon _2$$ $$\left( {|\varphi | < \pi /2} \right)$$ as shown in Fig. 1f, the permittivity tensor in the *xyz* coordinate system is given by16with $$\varepsilon _z = \varepsilon _1 + \varepsilon _3 - \varepsilon _2$$ and $$g = {\mathrm{sgn}}(\varphi )\sqrt {(\varepsilon _2 - \varepsilon _1)(\varepsilon _3 - \varepsilon _2)}$$. Since the in-plane duality condition is satisfied as $$\varepsilon _T = \varepsilon _2\mu _T$$ ($$\mu _T = 1$$), by rescaling the vacuum permittivity $$\varepsilon^{\prime}_0 = \varepsilon _2\varepsilon _0$$, we obtain the synthetic gauge potentials

17 $${{\hat {\cal{A}}}} = - \frac{{k_0{\kern 1pt} g}}{{2\sqrt {\varepsilon _2} }}{\mathbf{e}}_y\hat \sigma _1,\quad \quad {{\hat {\cal{A}}}}_0 = k_0^2{\kern 1pt} \frac{{\varepsilon _1\varepsilon _3 - \varepsilon _2^2}}{{2\varepsilon _2}}\hat \sigma _3,$$

and a uniform non-Abelian electric field polarized along the second principal axis

18 $$\hat {\cal{E}} = k_0^3\frac{{g{\kern 1pt} (\varepsilon _1\varepsilon _3 - \varepsilon _2^2)}}{{2\,\varepsilon _2^{3/2}}}{\mathbf{e}}_y\hat \sigma _2.$$

The two eigenstates in the *x*-direction are $| \mathord{\uparrow} \rangle = (1,0)^ \top$ and $| \mathord{\downarrow} \rangle = (0,1)^ \top$ corresponding to the two poles along the $\hat \sigma _3$-axis on the Bloch sphere (see Fig. 1h), the eigen wave vectors are ${\mathbf{k}}_ \uparrow = \sqrt {\varepsilon _1\varepsilon _3/\varepsilon _2} {\kern 1pt} k_0{\kern 1pt} {\mathbf{e}}_x$ and ${\mathbf{k}}_ \downarrow = \sqrt {\varepsilon _2} {\kern 1pt} k_0{\kern 1pt} {\mathbf{e}}_x$ respectively. Providing that $|\sqrt {\varepsilon _1\varepsilon _3} /\varepsilon _2 - 1|$ is small enough, the centroid trajectory of a beam mainly consisting of these two states satisfies

19 $$y(x) = Y_{{\mathrm{ZB}}}\left[ {{\mathrm{sin}}(k_{{\mathrm{ZB}}}(x - x_0) + \phi _0) - {\mathrm{sin}}\phi _0} \right],$$

where $\theta _0$, $\phi _0$ are the Euler angles of the initial spin $\vec s_0 = ({\mathrm{sin}}\theta _0{\mathrm{cos}}\phi _0,{\mathrm{sin}}\theta _0{\mathrm{sin}}\phi _0,{\mathrm{cos}}\theta _0)^ \top$, the ZB amplitude is

20 $$Y_{{\mathrm{ZB}}} = \frac{{{\cal{A}}_y^1{\kern 1pt} {\mathrm{{sin}}}\theta _0}}{{{\cal{A}}_0^3}} = \frac{{\sqrt {\varepsilon _2} g\,{\mathrm{sin}}\theta _0}}{{k_0(\varepsilon _2^{2} - \varepsilon _1\varepsilon _3)}},$$

and the ZB wave number

21 $$k_{{\mathrm{ZB}}} = \frac{{k_0^2(\varepsilon _2 - \varepsilon _1\varepsilon _3/\varepsilon _2)}}{{2k}} = \frac{{k_ \downarrow ^2 - k_ \uparrow ^2}}{{2k}} \approx k_ \downarrow - k_ \uparrow$$

is still determined by the beating of the two eigenstates. In the full-wave simulation of Fig. 1i, we obtained a trembling beam (also see Fig. 1g for its Fourier spectrum) where the decay of intensity along the beam is due to the beam divergence, the extracted centroid trajectory faithfully reproduces the analytic prediction of Eq. (19), shown by Fig. 1j. In Fig. 1h, the numerical spin trajectory on the Bloch sphere also verifies that the pseudo-spin precesses about the $\hat \sigma _3$-axis.

**Fig. 2:**
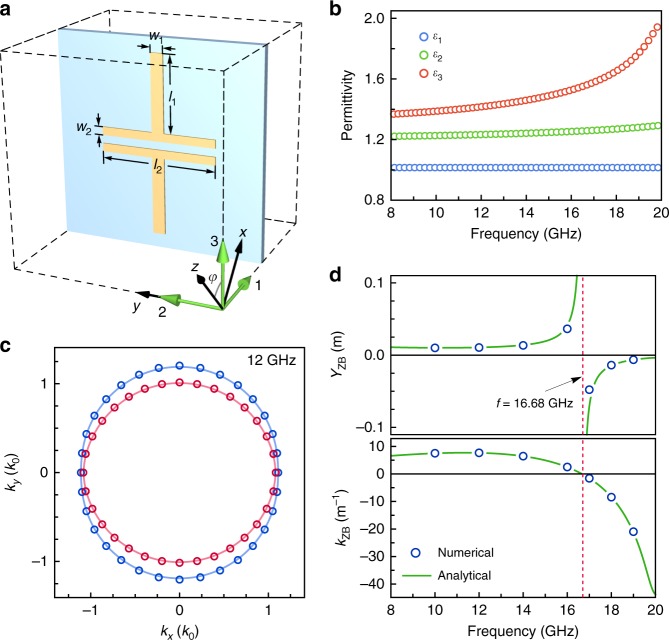
Design of a biaxial metamaterial. **a** Unit cell of the simple cubic structure with lattice constants *d* = 5 mm, where a FR4 PCB slab (light blue) with thickness 0.2 mm and relative permittivity $\varepsilon _{{\mathrm{pcb}}} = 3.3$ fills the coronal plane, and a copper structure of thickness 0.035 mm is printed on the PCB slab with the geometric parameters *l*_1_ = 2 mm, *l*_2_ = 2.8 mm, *w*_1_ = 0.3 mm, and *w*_2_ = 0.2 mm. **b** Dispersions of the retrieved relative permittivities along the three principal axes. **c** Isofrequency contours of the metamaterial in the *xy*-plane at 12 GHz, where the circular markers and solid curves correspond, respectively, to the real structure in **a** and the retrieved homogeneous medium. **d** Full-wave simulated (blue circles) and analytical (green curves) ZB amplitude *Y*_ZB_ and ZB wave number *k*_ZB_ in the homogenized media varying with frequency

In principle, the ZB effect induced by non-Abelian electric field can be observed in any natural and artificial biaxial non-magnetic materials. Here, we designed a simple metamaterial structure with the unit cell shown in Fig. 2a for realizing ZB in microwave regime. The copper strips on printed circuit board (PCB) layers support strong and anisotropic electric dipole resonances along principal axes labeled as 1, 2. Consequently, all the three principal values $\varepsilon _i\;(i = 1,2,3)$ of the effective permittivity are different, and their dispersions obtained by *S*-parameter retrieval approach^55^ are plotted in Fig. 2b. According to our theory, the ZB beams should travel in the *xy* plane whose orientation is determined by $\varepsilon _i$ and thus is frequency-dependent. As an example, we compared in Fig. 2c the isofrequency contours in *xy*-plane of the real structure and that of the homogenized medium at 12 GHz. Their perfect consistency confirms the retrieval result. To test the ZB effect in the metamaterial, we numerically simulated the ZB beams with a constant waist of 0.2 m propagating along *x*-direction in the retrieved media at some discrete frequencies and extracted the ZB amplitudes $Y_{{\mathrm{ZB}}}$ and ZB wave numbers $k_{{\mathrm{ZB}}}$. We find good agreement with the theoretical predictions given by Eqs. (20) and (21) as shown in Fig. 2d. Notably, both of the ZB amplitude and period tend to infinity at a singular frequency $f = 16.68{\kern 1pt} {\mathrm{GHz}}$, due to the fact that $\varepsilon _1\varepsilon _3 = \varepsilon _2^{{\kern 1pt} 2}$ is accidentally satisfied at the frequency such that the material is reduced to Abelian type with $\hat {\cal{E}} = 0$, and the beam splits into two branches^30^. We have also analyzed the finite width effect of the beam in the *z*-direction, and the analysis demonstrates that the 2D theory works well in the region where the two eigenmodes do not split away along the *z*-axis (see Supplementary Note 4).

### Non-Abelian Aharonov–Bohm system for light

ZB discussed in the previous section can be viewed as the interference between two eigenmodes, each of which evolves with Abelian dynamics. In this sense, ZB is an apparent non-Abelian effect. Next, we will introduce the genuine non-Abelian AB effect, which cannot be reduced to Abelian subsystems.

The AB effect covers a group of phenomena associated with the path-dependent phase factors for particles traveling in a field-free region, but with irremovable gauge potential ${\hat {\cal{A}}}_\mu$, the discovery of which confirmed the physical reality of gauge potentials and the nonlocality of gauge interactions^56,57^. The AB effect was first generalized to non-Abelian by Wu and Yang^32^, who showed that the scattering of nucleons (isospinors) around a non-Abelian flux tube (vortex) can generate peculiar phenomena. However, their governing Hamiltonian can be globally diagonalized into two decoupled Abelian subsystems under a proper gauge^58^, and all relevant phenomena can be interpreted from the superposition of the two subsystems. Hence, Wu and Yang’s proposal is now viewed as an apparent non-Abelian effect^10,44^. According to a rigorous definition^44^, a genuine non-Abelian AB system requires its holonomy group ${\mathrm{Hol}}({\hat {\cal{A}}})$ to be non-Abelian (see the “Methods” section and Supplementary Note 5). As such, there should exist such loops based at a fixed point that their non-Abelian AB phase factors (holonomies) are noncommutable, i.e. if a particle travels along two such loops in opposite sequences, the obtained AB phase factors would be different. This implies that at least two vortices exist in a genuine non-Abelian system^44^.

**Fig. 3:**
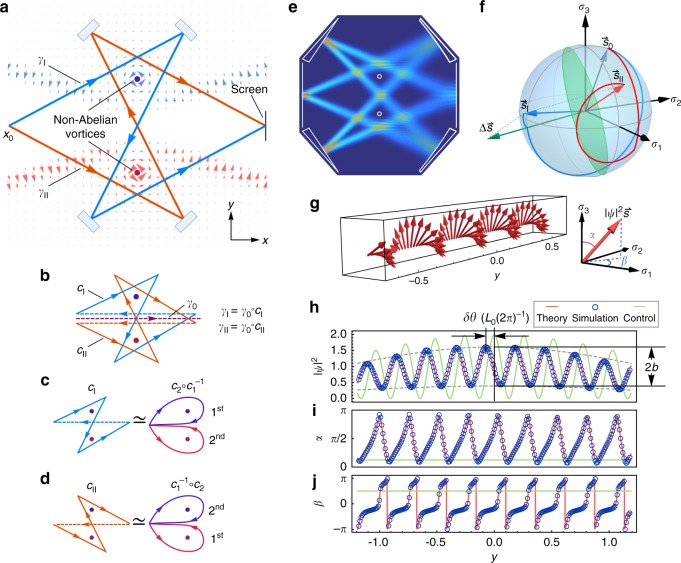
Genuine non-Abelian AB effect for light. **a** Sketch of the non-Abelian AB system with two optical paths $\gamma _{\mathrm{I}}$, $\gamma _{{\mathrm{II}}}$ interfering on the screen, where the background light blue (red) arrows denote the $\hat \sigma _1$ ($\hat \sigma _2$) component ${\cal{A}}^1$ $({\cal{A}}^2)$ of the non-Abelian vector potential. **b** $\gamma _{\mathrm{I}}$ ($\gamma _{{\mathrm{II}}}$) can be divided into a closed loop $c_{\mathrm{I}}$ ($c_{{\mathrm{II}}}$) and a common path *γ*_0_. **c**, **d** *c*_I_ and *c*_II_ can, respectively, deform continuously into a closed path that winds around the two vortices successively but in opposite sequences. **e** Snapshot of the simulated field intensity for the proposed non-Abelian optical interferometer with incident spinor $(1,{\mathrm{i}}1/5)^ \top$ for both beams and the vortex fluxes ${\mathrm{\Phi }}_1 = - 2\pi /3$, ${\mathrm{\Phi }}_2 = - \pi /3$. **f** Spin evolution on the Bloch sphere along two beams $\gamma _{\mathrm{I}}$, $\gamma _{{\mathrm{II}}}$, which share the same initial spin $\vec s_0$ but achieve different final spins $\vec s_{\mathrm{I}}$ and $\vec s_{{\mathrm{II}}}$. **g** Spin density interference corresponding to **e**, where each arrow denotes the local pseudo-spin density $|\psi |^2\vec s$ at a point on the screen. All of the local spins $\vec s(y)$ are perpendicular to $\Delta \vec s = \vec s_{\mathrm{I}} - \vec s_{{\mathrm{II}}}$, and thus fall on the green circle in **f**. The corresponding intensity interference $|\psi |^2(y)$ and the two Euler angles *α*, *β* of the local spins $\vec s(y)$ on the screen are shown in **h**–**j**, where blue circles and red curves indicate simulated and theoretical results, respectively, and $\delta \theta$, *b* are the phase shift and relative amplitude relative to the case of $\hat {\cal{A}} = 0$ (*L*_0_ is the period of $\Delta \theta (y)\ {\mathrm{{mod}}}\ 2\pi$). The green lines correspond to the “control experiment”

Indeed, we can use anisotropic and gyrotropic materials (see Table 1) to synthesize two vortices of SU(2) vector potential ${{\hat {\cal{A}}}} = {\cal{A}}^1\hat \sigma _1 + {\cal{A}}^2\hat \sigma _2$ $\left( {{{\hat {\cal{A}}}}_0 = 0} \right)$ with vanishing field $\hat {\cal{B}} = 0$ in the whole space except for two small domains, taken as point singularities for simplicity. Here, we provide the synopsis of our scheme, and more details are given in Supplementary Note 6 (also see Supplementary Note 8 for an alternative design). As illustrated in Fig. 3a, we demand ${{\hat {\cal{A}}}} = {\cal{A}}^1\hat \sigma _1$ $\left( {{\cal{A}}^2 = 0} \right)$ in the upper half-space, while ${{\hat {\cal{A}}}} = {\cal{A}}^2\hat \sigma _2$ $\left( {{\cal{A}}^1 = 0} \right)$ in the lower half-space. We also require that ${\cal{A}}^1$, ${\cal{A}}^2$ smoothly tend to zero in the middle region without overlap. In the vicinity of the upper (lower) singularity, ${\cal{A}}^1$ $\left( {{\cal{A}}^2} \right)$ forms an irrotational vortex carrying the flux $\Phi _1$ (Φ_2_) (see Supplementary Eq. (44) for the concrete expression of ${\hat {\cal{A}}}$ fulfilling these requirements). For a closed loop with a fixed base-point, its non-Abelian holonomy is invariant against continuous deformation of the path within the $\hat {\cal{B}} = 0$ region. As a consequence, for the two homotopy classes of loops [*c*_1_] and [*c*_2_] (where [*c*_i_] denote the path homotopy classes; see the “Methods” section), based at **x**_0_ and encircling the upper (for [*c*_1_]) or lower (for [*c*_2_]) vortex once, their holonomies are $\hat U_i = \hat {\cal{U}}_{[c_i]}[{\mathbf{x}}_0] = {\mathrm{{exp}}}\left[ {{\mathrm{i\Phi }}_i\hat \sigma _i} \right]$ (*i* = 1, 2,) respectively. As $\hat U_1$ and $\hat U_2$ do not commute with each other, this double-vortex system is a genuine non-Abelian AB system.

In order to realize the vector potential shown in Fig. 3a, the background media are set up as ${\mathbf{g}}_1 = - {\mathbf{g}}_2^ \ast$ (i.e. ${\mathbf{g}}_ + = 0$) and $\varepsilon _T = \varepsilon _z = \mu _T = \mu _z = {\mathrm{const}}{\mathrm{.}}$ to guarantee ${\hat {\cal{A}}}_0 \equiv 0$ and $V_0 = {\mathrm{const}}{\mathrm{.}}$ Also, we use reciprocal anisotropic materials with purely real off-block-diagonal components ${\mathbf{g}}_1 = - {\mathbf{g}}_2$ to build the vector potential ${{\hat {\cal{A}}}} = {\cal{A}}^1\hat \sigma _1$ in the upper half plane but gyrotropic materials with purely imaginary ${\mathbf{g}}_1 = {\mathbf{g}}_2$ to build ${\hat {\cal{A}}} = {\cal{A}}^2\hat \sigma _2$ in the lower half plane (see Supplementary Note 6 for details). As a result, we have designed a genuine non-Abelian AB system for light. Then, we will show how the genuine non-Abelian nature of the system can be detected from interference effects.

### Non-Abelian AB interference

Consider two coherent light beams with the same initial spin $\vec s_0$ propagating separately along the two folded paths $\gamma _{\mathrm{I}}$ and $\gamma _{{\mathrm{II}}}$, and finally superposing on the screen (Fig. 3a). For the trivial situation of ${{\hat {\cal{A}}}} = 0$, the two beams are uniformly polarized along the whole paths, thus their final states are given by $|\psi _i(y)\rangle = a(y){\mathrm{e}}^{{\mathrm{i}}\theta _i(y)}|s_0\rangle$ ($i = {\mathrm{I}},{\mathrm{II}}$), where $a(y)$ is the envelope of both beams on the screen, $|s_0\rangle$ is the normalized initial spinor at **x**_0_, and $\theta _i(y)$ denote the dynamic phases which have included the initial phases. The dynamic phase difference, $\Delta \theta (y) = \theta _{\mathrm{I}}(y) - \theta _{{\mathrm{II}}}(y)$, determines the interference pattern: $|\psi _{\mathrm{I}} + \psi _{{\mathrm{II}}}|^2(y) = 2a(y)[1 + \mathrm{cos}(\Delta \theta (y))]$.

In the presence of the two non-Abelian vortices of ${{\hat {\cal{A}}}}$, the two optical paths are unchanged thanks to the null gauge field. However, the gauge potential drives the pseudo-spins to rotate along the paths, and the two final states convert to

22 $$|\psi _i(y)\rangle = a(y)\hat U_{\gamma _i}{\mathrm{e}}^{{\mathrm{i}}\theta _i(y)}|s_0\rangle ,\quad (i = {\mathrm{I}},{\mathrm{II}})$$

where an additional non-Abelian AB phase factor $\hat U_{\gamma _i} = {\cal{P}}{\mathrm{exp}}\left[ {{\mathrm{i}}{\int}_{\gamma _i} {{\hat {\cal{A}}}} \cdot d{\mathbf{r}}} \right]$ appears in each state. The optical path of each beam can be regarded as a concatenation of a closed loop $c_i$ and a common path $\gamma _0$, i.e.,$\gamma _i = \gamma _0 \circ c_i$ ($i = {\mathrm{I}},{\mathrm{II}}$), as illustrated in Fig. 3b. The closed loop $c_{\mathrm{I}}$ can be further deformed continuously into two successive loops $c_2 \circ c_1^{ - 1}$, which winds around the upper vortex (clockwise) first and subsequently the lower vortex (anticlockwise) (Fig. 3c). Likewise, $c_{{\mathrm{II}}}$ is homotopic to $c_1^{ - 1} \circ c_2$, namely $c_{{\mathrm{II}}}$ winds around the lower vortex first before it does the upper vortex (Fig. 3d). Because of the noncommutativity of the sequences of winding around the two vortices, the AB phase factors of the two beams are different:

23 $$\hat U_{\gamma _{\mathrm{I}}} = \hat U_{\gamma _0}\hat U_2\hat U_1^{ - 1} \ne \hat U_{\gamma _{{\mathrm{II}}}} = \hat U_{\gamma _0}\hat U_1^{ - 1}\hat U_2.$$

Consequently, the two beams will end up with distinct spins $\vec s_{\mathrm{I}}$ and $\vec s_{{\mathrm{II}}}$ on the screen (Fig. 3f), and they will interfere with each other in a nontrivial way. The term spin density interference was coined for this phenomenon and it can be calculated as follows:

24 $$\langle \psi _{\mathrm{I}}(y) + \psi _{{\mathrm{II}}}(y)|{\kern 1pt} \vec {\hat \sigma } |\psi _{\mathrm{I}}(y) + \psi _{{\mathrm{II}}}(y)\rangle = |\psi |^2(y)\vec s(y).$$

Here, the angular bracket denotes the spinor inner product at a local position *y* on the screen, the obtained result describes the spin density distribution on the screen. The spin density can be further decomposed into two parts: the intensity interference $|\psi |^2(y)$ and the spin orientation interference $\vec s(y)$. The intensity interference part can be derived as

25 $$\begin{array}{*{20}{l}} {\left| \psi \right|^2(y)} \hfill & = \hfill & {2\left[ {a(y)^2 + {\mathrm{Re}}\langle \psi _{{\mathrm{II}}}|{\kern 1pt} \psi _{\mathrm{I}}\rangle (y)} \right]} \hfill \\ {} \hfill & = \hfill & {2a(y)^2\left[ {1 + {\mathrm{Re}}\left( {{\mathrm{e}}^{{\mathrm{i}}\Delta \theta (y)}\langle s_0|{\kern 1pt} \hat {\cal{U}}_{[c_0]}{\kern 1pt} |s_0\rangle } \right)} \right]} \hfill \\ {} \hfill & = \hfill & {2a(y)^2{\kern 1pt} \left[ {1 + b\,{\mathrm{cos}}\left( {\Delta \theta (y) + \delta \theta } \right)} \right],} \hfill \end{array}$$

where $\hat {\cal{U}}_{[c_0]} = \hat U_{\gamma _{{\mathrm{II}}}}^{ - 1}\hat U_{\gamma _{\mathrm{I}}} = \hat U_2^{ - 1}\hat U_1\hat U_2\hat U_1^{ - 1}$ is the non-Abelian holonomy of the closed path $c_0 = \gamma _{{\mathrm{II}}}^{ - 1} \circ \gamma _{\mathrm{I}}$. The nontrivial expectation value of the holonomy of $c_0$, $\langle s_0|{\kern 1pt} \hat {\cal{U}}_{[c_0]}|s_0\rangle = b{\kern 1pt} {\mathrm{e}}^{{\mathrm{i}}\delta \theta } \ne 1$, leads to a phase shift $\delta \theta$ and a change of the relative amplitude *b *(≤1) in comparison with the interference result of ${\hat {\cal{A}}} = 0$. In the mean time, the interfering spin orientation $\vec s(y)$ turns out to be always perpendicular to ${\mathrm{\Delta }}\vec s = \vec s_{\mathrm{I}} - \vec s_{{\mathrm{II}}}$, namely lying on the green great circle of $\vec s(y) \cdot {\mathrm{\Delta }}\vec s \equiv 0$ in Fig. 3f, and fluctuates around it (see Supplementary Note 7).

We have performed a full-wave simulation of this non-Abelian AB interference as shown in Fig. 3e. In the simulation, the envelope $a(y)$ of each beam is set to be Gaussian type with a central amplitude $a(0) = 1/\sqrt{2}$. The spin density interference is shown in Fig. 3g, with the intensity interference $|\psi |^2(y)$ in Fig. 3h, and the spin orientation given by Euler angles in Fig. 3i, j. In Fig. 3h–j, the blue circles are the simulated results, which are fairly consistent with the theoretical results (red curves) obtained from Eq. (24).

To demonstrate that the non-Abelian feature of the above design is indeed genuine, we consider a control experiment with an almost identical system except that the vector potential is ${\hat {\cal{A}}} \propto \hat \sigma _1$ in the whole space. In this case, $\hat U_i = {\mathrm{exp}}[{\mathrm{i\Phi }}_i\hat \sigma _1]$ (*i* = 1, 2) commute with each other, and their winding around the two vortices in opposite sequences gives the same AB phase factor $\hat U_{\gamma _{\mathrm{I}}} = \hat U_{\gamma _{{\mathrm{II}}}} = {\mathrm{exp}}\left[ {{\mathrm{i}}({\mathrm{\Phi }}_2 - {\mathrm{\Phi }}_1)\hat \sigma _1} \right]$. Thus, the interfering spin density is uniformly orientated, and there is no phase shift $\left( {\delta \theta \equiv 0} \right)$ and amplitude contraction $\left( {b \equiv 1} \right)$ compared with the case of ${\hat {\cal{A}}} = 0$ (see green lines in Fig. 3h–j).

### Measurement of Wilson loops

In Abelian AB systems, the AB phase factor (holonomy) of a closed loop only depends on the flux inside the loop but independent of the choice of gauge. However, in non-Abelian systems, the holonomy $\hat {\cal{U}}_{[c]}[{\mathbf{x}}_{0}]$ of a closed path *c* based at ${\mathbf{x}}_{0}$ varies as $\hat {\cal{U}}^{\prime}_{[c]}[{\mathbf{x}}_0] = \hat U({\mathbf{x}}_0)\hat {\cal{U}}_{[c]}[{\mathbf{x}}_0]\hat U^\dagger ({\mathbf{x}}_0)$, under a gauge transformation ${\hat {\cal{A}}}^\prime = \hat U{\hat {\cal{A}}}\hat U^\dagger + {\mathrm{i}}\hat U\nabla _T\hat U^\dagger$. Nevertheless, the trace of holonomy is an important gauge invariant observable, called the Wilson loop of the closed path *c*:

26 $$W(c) = {\mathrm{Tr}}\left( {{\cal{P}}{\mathrm{exp}}\left[ {{\mathrm{i}}{\oint}_{c} {{\hat {\cal{A}}}} \cdot{\mathrm{{{d}}}}{\mathbf{r}}} \right]} \right) = {\mathrm{Tr}}\,\hat {\cal{U}}_{[c]} = {\mathrm{Tr}}\,\hat {\cal{U}}^{\prime}_{[c]}.$$

In what follows, we show how to extract the Wilson loop of an arbitrary closed path via interferometry.

**Fig. 4:**
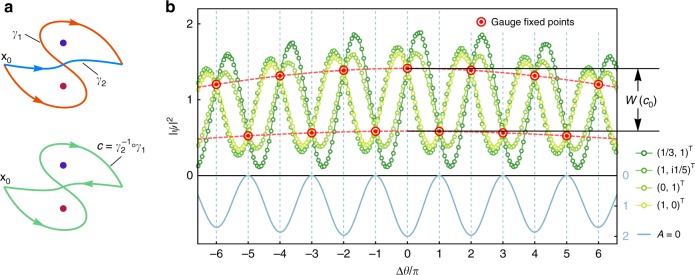
Extracting Wilson loops from gauge fixed points. **a** For two arbitrary beams *γ*_1_, *γ*_2_ interfering on the screen, the Wilson loop of the concatenate path $c = \gamma _2^{ - 1} \circ \gamma _1$ can be extracted from the interference fringes of the two beams. **b** Four intensity interference curves corresponding to four different incident spinors $(1,0)^ \top$, $(0,1)^ \top$, $(1,{\mathrm{i}}1/5)^ \top$, $(1/3,1)^ \top$ for the non-Abelian AB system shown in Fig. 3a with vortex fluxes $\Phi _1 = 0.22\pi$, ${\mathrm{\Phi }}_2 = - 0.33\pi$, where circles and solid curves represent numerical and analytical results, respectively. Their intersections, marked by red targets, are the gauge fixed points, which are located at the crests and troughs of the interference fringes (light blue curve) of $\hat {\cal{A}} = 0$. The maximal difference between the envelops of even and odd gauge fixed points gives the Wilson loop $W(c_0)$ of $c_0 = \gamma _{{\mathrm{II}}}^{ - 1} \circ \gamma _{\mathrm{I}}$

In order to obtain the Wilson loop of a homotopy class [*c*] in a non-Abelian AB system, we consider the interference of two beams along any two paths *γ*_1_ and *γ*_2_ as long as $\gamma _2^{ - 1} \circ \gamma _1 = c$ forms a closed loop in the class [*c*] as sketched in Fig. 4a. As we deduced in Eq. (25), the holonomy of *c*, together with the initial spinor $|s_0\rangle$, determines the phase shift and the relative amplitude through the term $\langle s_0|{\kern 1pt} \hat {\cal{U}}_{[c]}{\kern 1pt} |s_0\rangle = b\,{\mathrm{e}}^{{\mathrm{i}}\delta \theta }$. In fact, its real part depends solely on the Wilson loop of *c* (see proof in the “Methods” section):

27 $$W(c) = 2{\mathrm{Re}}{\kern 1pt} \langle s_0|{\kern 1pt} \hat {\cal{U}}_{[c]}|s_0\rangle = 2b\,{\mathrm{cos}}\,\delta \theta .$$

Thus, at certain positions $y_n$ satisfying $\Delta \theta (y_n) = n\pi$ (*n* belongs to integers), the intensities only depend on the Wilson loop of *c* and hence are fixed under gauge transformation:

28 $$\left| \psi \right|^2(y_n) \equiv a(y_n)^2\left[ {2 + ( - 1)^nW(c){\kern 1pt} } \right],$$

where the two beams are supposed to share the same envelope $a(y)$ on the screen, and the locations $y_n$ correspond to the crests and troughs in the interference fringes of ${{\hat {\cal{A}}}} = 0$. These particular points in the intensity fringes are termed the gauge fixed points for the closed path *c*. Since the change of incident spin at **x**_0_ is equivalent to a global gauge transformation, the interference fringes for different incident spins should intersect at the gauge fixed points.

Using the above method, we examine the two optical paths $\gamma _{\mathrm{I}}$, $\gamma _{{\mathrm{II}}}$ in Fig. 3a to extract the Wilson loop of $c_0 = \gamma _{{\mathrm{II}}}^{ - 1} \circ \gamma _{\mathrm{I}} \simeq c_2^{ - 1} \circ c_1 \circ c_2 \circ c_1^{ - 1}$. Figure 4b shows the intensity interference curves corresponding to four different incident spins. Indeed, they intersect exactly at the gauge fixed points (red targets in Fig. 4b) whose locations $y_n$ coincide with the crests and troughs of the interference fringe pattern for ${{\hat {\cal{A}}}} = 0$. By fitting the even and odd subsequences of the gauge fixed points, we obtain two curves $a(y)^2\left[ {2 \pm W(c_0)} \right]$ corresponding to the two red dashed lines in Fig. 4b. Thus, the Wilson loop *W*(*c*_0_) can be identified from the difference of the two dashed curves.

## Discussion

We have shown that the dynamics of 2D optical waves in a broad class of anisotropic media can be understood through an emergent SU(2) gauge interaction in real space. We predicted that the Zitterbewegung effect of light can be realized even in homogeneous anisotropic media, and we proposed a biaxial metamaterial to achieve synthetic non-Abelian electric field and ZB in microwave regime. We have also designed a genuine non-Abelian AB system with two synthetic non-Abelian vortices, and suggested a spin density interferometry to demonstrate the noncommutative feature of non-Abelian holonomies. Our scheme opens the door to the colorful non-Abelian world for light. In addition to inspiring new ideas to manipulate the flow and polarization of light, the scheme offers an optical platform to study physical effects relevant to SU(2) gauge fields, such as synthetic spin–orbit coupling^59^ and topological band structures in periodic non-Abelian gauge fields^60–63^. Furthermore, since the SU(2) gauge field description is valid for photons down to quantum scale, this approach might be applicable to the design of geometric gates for realizing non-Abelian holonomic quantum computation^64,65^ with photons.

## Methods

### Notations

In this paper, vectors in real space and in pseudo-spin space are indicated, respectively, by bold letters and letters with an overhead arrow “→”. Letters with an overhead bidirectional arrow “↔” denote two-order tensors in real space. Symbols with an overhead hat “∧” denote operators acting on the spinor wave functions. We use Greek letters, e.g. $\mu ,\nu$, to denote indices of (2+1)-dimensional spacetime. Latin letters *i*, *j* denote 2D spatial coordinate indices, and Latin letters *a*, *b*, *c* denote indices in pseudo-spin space. We follow the Einstein summation convention for repeated indices. The orthonormal coordinate bases in real space and pseudo-spin space are expressed as **e**_*i*_ and $\vec e_a$, respectively.

### SU(2) gauge covariance of 2D Maxwell equations

In block-diagonalized duality symmetric media, $${\kern 1pt} \mathord{\buildrel{\lower3pt\hbox{$\scriptscriptstyle \leftrightarrow$}} \\ \over \varepsilon } /\varepsilon _0 = {\kern 1pt} \mathord{\buildrel{\lower3pt\hbox{$\scriptscriptstyle \leftrightarrow$}} \\ \over \mu } /\mu _0 = {\mathrm{diag}}(\mathord{\buildrel{\lower3pt\hbox{$\scriptscriptstyle \leftrightarrow$}} \\ \over \varepsilon } _T,\varepsilon _z)$$, the Maxwell’s equations for 2D waves can be rearranged as29with $$\Psi = ({\mathbf{E}}_T,\eta _0{\mathbf{H}}_T,E_z,\eta _0H_z)^ \top$$. For an arbitrary (global) transformation $$\hat U \in {\mathrm{SU}}(2)$$ acting on $$|\psi \rangle = (E_z,\eta _0H_z)^ \top$$, the corresponding transformation for $$\Psi$$ is defined as

30 $$\tilde U = \hat U_T \oplus \hat U = (\hat \sigma _2\hat U\hat \sigma _2) \oplus \hat U,$$

which belongs to a 4D representation of SU(2). It turns out that ${\cal{M}}$ and ${\cal{N}}$ defined in Eq. (29) transform according to31

32 $$\tilde U{\cal{N}}\tilde U^\dagger = {\cal{N}}.$$

Hence, the 2D Maxwell equations are invariant under this SU(2) transformation. As the EM duality transformation $\hat R \in \mathrm{SO}(2)$ is a special case of $\hat U$, the emergent SU(2) symmetry for the 2D Maxwell equations in block-diagonalized duality symmetric materials is indeed the generalization of the original EM duality symmetry.

If $\hat U(x,y)$ is dependent on the *x*, *y* coordinates, the transformation of ${\cal{M}}$ changes to

33 $$\tilde U(x,y){\cal{M}}\tilde U^\dagger (x,y) = {\cal{M}} + {\mathrm{\Delta }}{\cal{M}}$$

with an additional term34where ${\cal{A}}^a\hat \sigma _a = {\mathrm{i}}\hat U\nabla _T\hat U^\dagger$ is precisely the vector potential induced purely by the gauge transformation, and only the components ${\cal{A}}^1,{\cal{A}}^2$ are supposed to exist. If we move the term ${\mathrm{\Delta }}{\cal{M}}$ to the right side of the Maxwell equation (29), it can be alternatively interpreted as a part of the constitutive tensor. By rotating $\Psi$ to the ordinary basis of EM field,35we obtain explicitly the contribution of ${\mathrm{\Delta }}{\cal{M}}$ to the material tensors

36 $$\left( {\begin{array}{*{20}{c}} {\overleftrightarrow \varepsilon } & 0 \\ 0 & {\overleftrightarrow \mu } \end{array}} \right) = U_0({\cal{N}} - {\mathrm{\Delta }}{\cal{N}}/k_0)U_0^\dagger ,$$

which shows that the effective SU(2) vector potential $${\hat {\cal{A}}}$$ emerging in $${\mathrm{\Delta }}{\cal{M}}$$ just corresponds to the off-diagonal terms of $$\mathord{\buildrel{\lower3pt\hbox{$\scriptscriptstyle \leftrightarrow$}} \\ \over \varepsilon }$$, $$\mathord{\buildrel{\lower3pt\hbox{$\scriptscriptstyle \leftrightarrow$}} \\ \over \mu }$$:

37 $${\mathbf{g}}_1 = - {\mathbf{g}}_2^ \ast = {\mathbf{e}}_z \times ({\cal{A}}^1 + {\mathrm{i}}{\cal{A}}^2)/k_0.$$

Indeed, this relation is valid for arbitrary $${\hat {\cal{A}}} = {\cal{A}}^1\hat \sigma _1 + {\cal{A}}^2\hat \sigma _2$$ but not limited to the pure gauge case $${\hat {\cal{A}}} = {\mathrm{i}}\hat U\nabla _T\hat U^\dagger$$. Furthermore, this correspondence can be generalized to any media satisfying in-plane duality condition $${\kern 1pt} \mathord{\buildrel{\lower3pt\hbox{$\scriptscriptstyle \leftrightarrow$}} \\ \over \varepsilon } _T = \alpha \mathord{\buildrel{\lower3pt\hbox{$\scriptscriptstyle \leftrightarrow$}} \\ \over \mu } _T$$ where SU(2) scalar potential may also appear (Supplementary Note 1).

### Quasi-degenerate approximation for ZB

Eq. (2) is essentially the stationary wave equation describing spin-1/2 particles coupling to the background SU(2) gauge fields without any approximation. However, the semiclassical trajectories of non-degenerate eigenmodes often split away from each other. To manifest the coupling effects of different eigenmodes in the geometric optics, the media of concern are usually assumed to be weakly anisotropic^14^. Nevertheless, if the eigenmodes are approximately degenerate in a particular direction of wave vector but not necessarily in all directions, it turns out that an intact wave composed of modes in the vicinity of the quasi-degenerate direction can be described adequately by the semiclassical approach including the interaction between eigenmodes in their interfering region^66^.

In homogeneous non-Abelian media, we separate the effective Hamiltonian into two parts:

38 $$\hat H({\mathbf{k}}) = \underbrace {\left[ {\frac{1}{{2m}}\left( {{\mathbf{k}}^2 + ({{\hat {\cal{A}}}})^2} \right) + V_0} \right]\hat \sigma _0}_{\hat H_0({\mathbf{k}})} + \underbrace {\left( {\frac{{ - 1}}{m}{\mathbf{k}} \cdot {{\hat {\cal{A}}}} - {{\hat {\cal{A}}}}_0} \right)}_{\delta \hat H({\mathbf{k}}) = \vec \Omega \cdot \vec {\hat \sigma } /2}.$$

If only $\hat H_0$ is present, the isofrequency surface is a doubly degenerate sphere with the radius $k = \sqrt { - 2m{\kern 1pt} V_0 - ({{\hat {\cal{A}}}})^2}$. When $\delta \hat H({\mathbf{k}})$ is taken into account, as long as it is sufficiently small for a given direction ${\mathbf{e}}_k$, the two eigenstates can be regarded as quasi-degenerate at the wave vector

39 $${\mathbf{k}} = k{\mathbf{e}}_k = \sqrt { - 2mV_0 - ({{\hat {\cal{A}}}})^2} {\mathbf{e}}_k,$$

and we can implement the eikonal approximation to the wave function mainly superposed by the two quasi-degenerate modes^66^: $|\psi\rangle = \widetilde \psi ({\mathbf{r}})\mathrm{exp}({\mathrm{i}}{\mathbf{k}} \cdot {\mathbf{r}})$ with a slowly varying envelope $\widetilde \psi ({\mathbf{r}})$ (i.e. $\left| {\nabla \widetilde \psi /\widetilde \psi } \right| \ll k$). Subsituting $|\psi\rangle$ into the wave equation (2), we obtain the equation of $\widetilde \psi$ with accuracy up to the first order of *k*:

40 $${\mathrm{i}}\widehat {\mathbf{v}} \cdot \nabla \widetilde \psi = \hat H({\mathbf{k}})\widetilde \psi .$$

By adopting the ansatz that the velocity operator $\widehat {\mathbf{v}} = \partial \hat H({\mathbf{k}})/\partial {\mathbf{k}} = ({\mathbf{k}} - {{\hat {\cal{A}}}})/m$ can be replaced by its averaged value $\langle \widehat {\mathbf{v}}\rangle$ over the transverse cross section of an optical beam, we find that the operator $\widehat {\mathbf{v}} \cdot \nabla \to \langle \widehat {\mathbf{v}}\rangle \cdot \nabla = {\mathrm{{d}}}/{\mathrm{{d}}}\tau$ corresponds to the total derivative with respect to the ray parameter $\tau$ along the beam. Therefore, Eq. (40) is reformulated into a time-dependent Schrödinger equation

41 $${\mathrm{i}}\frac{d}{{d\tau }}\widetilde \psi = \hat H({\mathbf{k}})\widetilde \psi .$$

Consequently, Eqs. (8-10) can be directly obtained in terms of Ehrenfest theorem.

### Relation between Poynting vector and velocity operator

The in-plane projection of the time-averaged Poynting vector ${\bar{\mathbf{S}}}_{\mathrm{{T}}}$ for monochromatic waves can be written as

42 $$\begin{array}{*{20}{l}} {{\bar{\mathbf{S}}}_{\mathrm{{T}}}} \hfill & = \hfill & {{\kern 1pt} \frac{1}{2}{\mathrm{Re}}\left[ {{\mathbf{E}}_z^ \ast \times {\mathbf{H}}_{\mathrm{{T}}} + {\mathbf{E}}_{\mathrm{{T}}}^ \ast \times {\mathbf{H}}_z} \right]} \hfill \\ {} \hfill & = \hfill & {{\kern 1pt} \frac{1}{2}{\mathrm{Re}}\left[ {({\mathbf{E}}_z^ \ast ,{\mathbf{H}}_z^ \ast )\left( {\begin{array}{*{20}{c}} 0 & {\mathop{{\rm{I}}}\limits^{\leftrightarrow} \times \mathop{{\rm{I}}}\limits^{\leftrightarrow}} \\ { - {\kern 1pt} \mathop{{\rm{I}}}\limits^{\leftrightarrow} \times \mathop{{\rm{I}}}\limits^{\leftrightarrow}} & 0 \end{array}} \right)\left( {\begin{array}{*{20}{c}} {{\mathbf{E}}_{\mathrm{{T}}}} \\ {{\mathbf{H}}_{\mathrm{{T}}}} \end{array}} \right)} \right]} \hfill \\ {} \hfill & = \hfill & {{\kern 1pt} \frac{1}{{2\eta _0}}{\mathrm{Re}}\left[ {(E_z^ \ast ,\eta _0H_z^ \ast )(i\hat \sigma _2\varepsilon ^{izj}{\mathbf{e}}_i)\left( {\begin{array}{*{20}{c}} {{\mathbf{E}}_{\mathrm{{T}}}} \\ {\eta _0{\mathbf{H}}_{\mathrm{{T}}}} \end{array}} \right)_j} \right].} \hfill \end{array}$$

Substituting Maxwell’s equations into Eq. (42) yields

43 $$\begin{array}{*{20}{l}} {\overline {\mathbf{S}} _{\mathrm{{T}}}} \hfill & = \hfill & {{\kern 1pt} \frac{1}{{2\eta _0}}{\mathrm{Re}}\langle \psi |({\mathrm{i}}\hat \sigma _2\epsilon ^{izj}{\mathbf{e}}_i)\frac{{ - {\mathrm{i}}{\kern 1pt} \hat \sigma _2\,\epsilon _{jkz}}}{{k_0\,\varepsilon _T}}(\widehat {\mathbf{p}} - {\hat {\cal{A}}}_{(c)})^k|\psi \rangle } \hfill \\ {} \hfill & = \hfill & {{\kern 1pt} \frac{1}{{2\eta _0\,k_0}}{\mathrm{Re}}\langle \psi |\frac{1}{{2m}}(\widehat {\mathbf{p}} - {\hat {\cal{A}}}_{(c)})|\psi \rangle } \hfill \\ {} \hfill & = \hfill & {{\kern 1pt} \frac{1}{{4\mu _0\,\omega _0}}\langle \psi |\frac{1}{m}({\mathbf{k}} - {\hat {\cal{A}}})|\psi \rangle = \frac{1}{{4\mu _0\,\omega _0}}\langle \psi |\widehat {\mathbf{v}}|\psi \rangle ,} \hfill \end{array}$$

where ${\hat{\mathcal{A}}}_{(c)}=\frac{k_0}{2}\left\{\left[({\mathbf{g}}_{1}-{\mathbf{g}}_2)\times{\mathbf{e}}_z\right] \hat{\sigma}_1 -{\mathrm{i}}\left[({\mathbf{g}}_1+{\mathbf{g}}_2)\times{\mathbf{e}}_z\right]\hat{\sigma}_2 \right\}$. In the third step, we replaced $\widehat {\mathbf{p}}$ with **k** according to the eikonal approximation. As a result, the total in-plane energy flux over a transverse cross section of the optical beam is propotional to the expectation value of the velocity operator:

44 $$\langle \overline {\mathbf{S}} _{\mathrm{{T}}}\rangle = \frac{1}{{4\mu _0\omega _0}}{\int} {\mathrm{{d}}}{\mathbf{r}}_ \bot \langle \psi |\widehat {\mathbf{v}}|\psi \rangle = \frac{1}{{4\mu _0\omega _0}}\langle \widehat {\mathbf{v}}\rangle .$$

And it shows that the time-averaged Poynting vector $\overline {\mathbf{S}} _T$ is invariant under the gauge transformation Eq. (30) for EM fields (Supplementary Note 2).

### Holonomy and genuine non-Abelian AB system

From a geometric viewpoint, gauge potential and field in the physical space *M* can be described as the connection and curvature in a *G*-principle fiber bundle^32^, where the physical space serves as the base manifold, and *G* denotes the gauge group, in our case $G = {\mathrm{SU}}(2)$. Consider a particle (wave packet) travels in the physical space. Along its trajectory *γ*, the gauge potential engenders a matrix-valued geometric phase factor ${\cal{P}}{\mathrm{{exp}}}\left[ {{\mathrm{i}}{\int}_\gamma {\hat {\cal{A}}}_\mu {\mathrm{{d}}}x^\mu } \right] \in G$ (${\cal{P}}$ denotes path-ordering) on the state vector, corresponding to the parallel transport of the state in the bundle space. In particular, for a closed path *c* starting and ending at the same point $c(0) = c(1) = {\mathbf{x}}_0$, the phase factor of *c*,

45 $$\hat {\cal{U}}_c({{\hat {\cal{A}}}}) = {\cal{P}}{\mathrm{exp}}\left[ {{\mathrm{i}}{\oint}_{c} {{\hat {\cal{A}}}}_\mu {\mathrm{{d}}}x^\mu } \right],$$

is called the holonomy of the closed path *c* with respect to the gauge ${\hat {\cal{A}}}$. The collection of the holonomies corresponding to all those closed paths based at the same point **x**_0_ forms a subgroup of the gauge group *G*:

46 $${\mathrm{Hol}}({\hat {\cal{A}}}) = \left\{ {\hat {\cal{U}}_c({\hat {\cal{A}}})|c(0) = c(1) = {\mathbf{x}}_0} \right\} \subseteq G,$$

which is the holonomy group for the gauge ${\hat {\cal{A}}}$. In the literature, a gauge system is regarded as genuinely non-Abelian if and only if the holonomy group is a non-Abelian group, namely the holonomies of some loops are noncommutable with each other^10,44^. If the base manifold is simply a Euclidean space, the noncommutativity of holonomies can be traced back to noncommutable gauge fields $[\hat {\cal{F}}_{\mu \nu },\hat {\cal{F}}_{\mu^{\prime}\nu^{\prime}}] \ne 0$. However, if the base manifold possesses nontrivial topology, noncommutative holonomies can be achieved even though the gauge field vanishes everywhere (i.e. AB systems).

For an AB system, the corresponding fiber bundle is a flat bundle, since the curvature (field) $\hat {\cal{F}}_{\mu \nu } = 0$ in the whole base manifold *M* (flux regions are excluded from *M*). Here, the topology of the base manifold is characterized by its first fundamental group,

47 $$\pi _1(M) = \{ [c]{\kern 1pt} |{\kern 1pt} c(0) = c(1) = {\mathbf{x}}_0\} ,$$

which is the set of path homotopy equivalent classes [*c*] of closed paths based at **x**_0_. Path homotopy is a topologically equivalent relation “$\simeq$” for paths. If two paths *c*_1_, *c*_1_ with the same fixed base-point **x**_0_ can deform into each other continuously, they are said to be path homotopic $c_1 \simeq c_2$ and to belong to the same homotopy class [*c*_1_]. In flat bundles, the holonomies (AB phase factors) of all loops in the same homotopy class [*c*] are identical: $\hat {\cal{U}}_{[c]}$ (see proof in Supplementary Note 5). Based on this property, two necessary conditions for genuine non-Abelian AB systems can be obtained^44^:

1. The gauge group *G* is non-Abelian;
2. The first fundamental group $\pi _1(M)$ is non-Abelian.

According to the second criterion, the Wu–Yang AB system is not genuinely non-Abelian, because the fundamental group of its base manifold (a punctured plane ${\Bbb R}^2 - {\mathbf{0}}$) is an Abelian group $\pi _1({\Bbb R}^2 - {\mathbf{0}}) = {\Bbb Z}$. However, for a twice-punctured plane as shown in Fig. 3a, its fundamental group is the free group on two generators, ${\Bbb Z} \ast {\Bbb Z}$ (where * denotes a free product), which is non-Abelian^67^. Therefore, a twice-punctured plane is a qualified prototype of a genuine non-Abelian AB system.

### Gauge fixed points

The derivation of the intensity interference given by Eq. (25) is in fact valid for two arbitrary interfering beams *γ*_1_, *γ*_1_ with the same initial spin $\vec s_0$ and final envelop $a(y)$: $|\psi |^2 = 2a(y)[1 + {\mathrm{Re}}({\mathrm{e}}^{{\mathrm{i\Delta }}\theta (y)}\langle s_0|{\kern 1pt} \hat {\cal{U}}_{[c]}{\kern 1pt} |s_0\rangle )]$, where $\hat {\cal{U}}_{[c]}$ is the holonomy of the closed path $c = \gamma _2^{ - 1} \circ \gamma _1$. Since $\hat {\cal{U}}_{[c]} \in {\mathrm{SU}}(2)$, it can be generically expressed as

48 $$\hat {\cal{U}}_{[c]} = \left( {\begin{array}{*{20}{c}} {u_1} & {u_2} \\ { - u_2^ \ast } & {u_1^ \ast } \end{array}} \right),\quad {\mathrm{with}}\quad |u_1|^2 + |u_2|^2 = 1.$$

The Wilson loop reads $W(c) = {\mathrm{Tr}}\,\hat {\cal{U}}_{[c]} = 2\,{\mathrm{Re}}\,(u_1).$ For an arbitrary spinor state $|s_0\rangle = \left( {{\mathrm{cos}}\frac{\alpha }{2}{\mathrm{e}}^{ - {\mathrm{i}}\beta /2},{\mathrm{sin}}\frac{\alpha }{2}{\mathrm{e}}^{{\mathrm{i}}\beta /2}} \right)^ \top$, we have

49 $$\begin{array}{*{20}{l}} {} \hfill & {} \hfill & {\langle s_0|{\kern 1pt} \hat {\cal{U}}_{[c]}{\kern 1pt} |s_0\rangle = b{\mathrm{e}}^{{\mathrm{i}}\delta \theta }} \hfill \\ {} \hfill & = \hfill & {{\mathrm{Re}}(u_1) + {\mathrm{i}}\left[ {{\mathrm{cos}}\,\alpha {\kern 1pt} \,{\mathrm{Im}}\,(u_1) + {\mathrm{sin}}\,\alpha \,{\mathrm{Im}}\,(u_2{\mathrm{e}}^{{\mathrm{i}}\beta })} \right].} \hfill \end{array}$$

Therefore, the identity in Eq. (27) is established for any $|s_0\rangle$.

In fact, different incident spinors can interconvert through a global gauge transformation: $|s^{\prime}_0\rangle = \hat U|s_0\rangle$. Hence, the relation in Eq. (27) is straightforward

50 $$\begin{array}{*{20}{l}} {2{\kern 1pt} {\mathrm{Re}}\langle s^{\prime}_0|{\kern 1pt} \hat {\cal{U}}_{[c]}{\kern 1pt} |s^{\prime}_0\rangle } \hfill & = \hfill & {2{\kern 1pt} {\mathrm{Re}}{\kern 1pt} \langle s_0|{\kern 1pt} \hat U^{ - 1}\hat {\cal{U}}_{[c]}\hat U{\kern 1pt} |s_0\rangle } \hfill \\ {} \hfill & = \hfill & {{\mathrm{Tr}}\left( {\hat U^{ - 1}\hat {\cal{U}}_{[c]}\hat U} \right) \equiv W(c).} \hfill \end{array}$$

As a result, at the positions such that $\Delta \theta (y_n) = n\pi$, i.e. at the crests and troughs of the original interference fringes when ${\hat {\cal{A}}} = 0$, the intensities given in Eq. (28) are fixed for arbitrary incident spins, yet they are only determined by the Wilson loop *W*(*c*), provided that the dynamic phases of *γ*_1_, *γ*_2_ are unchanged.

For the two optical path $\gamma _{\mathrm{I}}$, $\gamma _{{\mathrm{II}}}$ in Fig. 3, the Wilson loop of $c_0 = \gamma _{{\mathrm{II}}}^{ - 1} \circ \gamma _{\mathrm{I}}$ is determined by the fluxes of the two vortices as $W(c_0) = 2 - 4{\mathrm{sin}}^2{\mathrm{\Phi }}_1{\mathrm{sin}}^2{\mathrm{\Phi }}_2$. Therefore, if ${\mathrm{sin}}^2{\mathrm{\Phi }}_1{\mathrm{sin}}^2{\mathrm{\Phi }}_2 = 1/2$, *W*(*c*_0_) will be reduced to zero, and the two dashed curves in Fig. 4 will completely overlap (see Supplementary Note 7 for details).

### Simulation of non-Abelian AB interference

The full-wave results of the non-Abelian AB interference shown in Figs. 3 and 4 are simulated using the commercial software COMSOL Multiphysics. In order to avoid spin–flip after reflection, the mirrors shown in Fig. 3a, e are made of an impedance-matched material, namely $\varepsilon _m/\mu _m = 1$, with a lower refractive index than the surrounding media to achieve total reflection at their surfaces. Meanwhile, the two mirrors on the right-hand side in Fig. 3e are slightly concave, so that the reflected beams with reduced widths can bypass the two singularities.

## Supplementary information

Supplementary Information

## Data Availability

The authors declare that all data supporting the findings of this study are available from the corresponding authors upon reasonable request.
