# Supplementary material for: Non-Abelian gauge field optics
Source: Nat Commun. 2019 Jul 16;10:3125. doi: 10.1038/s41467-019-10974-8 (PMC6635576; doi:10.1038/s41467-019-10974-8)
Supplement: Supplementary file 1 — Supplementary Information [file 41467_2019_10974_MOESM1_ESM.pdf]

Supplementary information

Non-Abelian gauge field optics

Chen *et al.*

Contents

Supplementary Note 1. Synthetic U(2) gauge fields in real space for light 3

Supplementary Note 2. Gauge transformation between synthetic gauge field systems 7

Supplementary Note 3. Analogy with *Zitterbewegung* effect arising from Dirac cone 9

Supplementary Note 4. ZB effect of 3D beams with finite width in z direction 11

Supplementary Note 5. Theory for genuine non-Abelian Aharonov-Bohm system 13

Supplementary Note 6. Material construction of non-Abelian AB system 17

Supplementary Note 7. Non-Abelian AB interference 20

Supplementary Note 8.Designing non-Abelian AB system with gyroelectric materials 23

Supplementary References 25

Supplementary Note 1. Synthetic U(2) gauge fields in real space for light

In the main text, the materials considered are restricted to anisotropic media without magnetoelectric (ME) coupling. In that case, an incomplete set of synthetic $U(2) = \mathrm{SU}(2) \rtimes \mathrm{U}(1)$ gauge potentials is obtained, while the $\mathcal{A}^3\hat{\sigma}_3$ component of SU(2) vector potential and U(1) vector potential are absent. Here, we consider the more general case of bi-anisotropic media through adding a specific ME coupling term, in order to obtain a complete set of U(2) gauge potentials. In frequency domain, the constitutive relations of nondissipative and nondispersive bi-anisotropic media read

$$\mathbf{D}_\omega = \overleftrightarrow{\varepsilon} \cdot \mathbf{E}_\omega + \overleftrightarrow{\chi}_{em} \cdot \mathbf{H}_\omega, \qquad \mathbf{B}_\omega = \overleftrightarrow{\mu} \cdot \mathbf{H}_\omega + \overleftrightarrow{\chi}_{me} \cdot \mathbf{E}_\omega, \qquad (\omega > 0) \tag{1}$$

with the constitutive coefficient tensors

$$\overleftrightarrow{\varepsilon}/\varepsilon_0 = \left(\begin{array}{c|c} \overleftrightarrow{\varepsilon}_T & \mathbf{g}_1 \\ \hline \mathbf{g}_1^\dagger & \varepsilon_z \end{array}\right), \quad \overleftrightarrow{\mu}/\mu_0 = \left(\begin{array}{c|c} \overleftrightarrow{\mu}_T & \mathbf{g}_2 \\ \hline \mathbf{g}_2^\dagger & \mu_z \end{array}\right), \quad \overleftrightarrow{\chi}_{em} = \overleftrightarrow{\chi}_{me}^\dagger = \overleftrightarrow{\chi} = \frac{1}{c}\left(\begin{array}{c|c} \mathbf{0} & \mathbf{t}_1 \\ \hline \mathbf{t}_2^\intercal & \chi_z \end{array}\right). \tag{2}$$

Here, all parameters are functions of x and y. The only constraint on $\overleftrightarrow{\varepsilon}$, $\overleftrightarrow{\mu}$ is the "in-plane duality" $\overleftrightarrow{\varepsilon}_T = \alpha\overleftrightarrow{\mu}_T \in \mathbb{R}$ (α is an arbitrary positive constant). In the following derivations, we let $\alpha = 1$ for convenience, and the results of $\alpha \neq 1$ is straightforward via replacing $\varepsilon_0 \to \varepsilon_0' = \alpha\,\varepsilon_0$ and $c \to c' = 1/\sqrt{\varepsilon_0'\mu_0}$ in Eq. (2). The ME coupling tensors $\overleftrightarrow{\chi}_{em}$, $\overleftrightarrow{\chi}_{me}$ are assumed to be purely real and do not have in-plane block for convenience.

Derivation of in-plane wave equation

In this section, we derive the 2D wave equations for monochromatic waves in the bi-anisotropic media given by Eq. (2), where a complete set of synthetic U(2) gauge potentials emerge. For 2D propagating waves, the fields **E**, **H** are only functions of x, y, so all terms associated with $\partial/\partial z$ are dropped, and the source-free Maxwell's equations for the complex-valued EM fields (analytic signals) can be expressed as

$$\left[\begin{pmatrix} \nabla_T\times & 0 \\ 0 & \nabla_T\times \end{pmatrix} + \frac{\partial}{\partial t}\frac{1}{c}\begin{pmatrix} \tilde{\mathbf{t}}_2\times & \tilde{\mathbf{g}}_2\times \\ -\tilde{\mathbf{g}}_1\times & -\tilde{\mathbf{t}}_1\times \end{pmatrix}\right]\begin{pmatrix} \mathbf{E}_z \\ \eta_0\mathbf{H}_z \end{pmatrix} = -\frac{\partial}{\partial t}\frac{1}{c}\begin{pmatrix} 0 & \overleftrightarrow{\mu}_T \\ -\overleftrightarrow{\varepsilon}_T & 0 \end{pmatrix}\begin{pmatrix} \mathbf{E}_T \\ \eta_0\mathbf{H}_T \end{pmatrix}, \tag{3a}$$

$$\left[\begin{pmatrix} \nabla_T\times & 0 \\ 0 & \nabla_T\times \end{pmatrix} - \frac{\partial}{\partial t}\frac{1}{c}\begin{pmatrix} \tilde{\mathbf{t}}_1\times & \tilde{\mathbf{g}}_2^\dagger\times \\ -\tilde{\mathbf{g}}_1^\dagger\times & -\tilde{\mathbf{t}}_2\times \end{pmatrix}\right]\begin{pmatrix} \mathbf{E}_T \\ \eta_0\mathbf{H}_T \end{pmatrix} = -\frac{\partial}{\partial t}\frac{1}{c}\begin{pmatrix} \chi_z & \mu_z \\ -\varepsilon_z & -\chi_z \end{pmatrix}\begin{pmatrix} \mathbf{E}_z \\ \eta_0\mathbf{H}_z \end{pmatrix}, \tag{3b}$$

where $\nabla_T = (\partial_x, \partial_y)^\intercal$, $\eta 0 = \sqrt{\mu_0/\varepsilon_0}$, $\tilde{\mathbf{g}}_i = \mathbf{e}_z \times \mathbf{g}_i = (-g_{iy}, g_{ix})^\intercal$, $\tilde{\mathbf{t}}_i = \mathbf{e}_z \times \mathbf{t}_i = (-t_{iy}, t_{ix})^\intercal$ $(i = 1, 2)$. For monochromatic waves **E**, **H** $\propto \exp(-i\omega t)$, after substituting $\partial/\partial t \to -i\omega$, Eq. (3) can be rewritten using Pauli matrices as

$$\left[\hat{\sigma}_0\left(\nabla_T \times + ik_0\tilde{\mathbf{t}}_-\times\right) + \hat{\sigma}_1(ik_0\tilde{\mathbf{g}}_-\times) - i\hat{\sigma}_2(ik_0\tilde{\mathbf{g}}_+\times) - \hat{\sigma}_3(ik_0\tilde{\mathbf{t}}_+\times)\right]\begin{pmatrix} \mathbf{E}_z \\ \eta_0\mathbf{H}_z \end{pmatrix} = \hat{\sigma}_2\left(-k_0\overleftrightarrow{\varepsilon}_T\right)\begin{pmatrix} \mathbf{E}_T \\ \eta_0\mathbf{H}_T \end{pmatrix}, \tag{4}$$

$$\left[\hat{\sigma}_0\left(\nabla_T \times + ik_0\tilde{\mathbf{t}}_-\times\right) - \hat{\sigma}_1(ik_0\tilde{\mathbf{g}}_-^\dagger\times) + i\hat{\sigma}_2(ik_0\tilde{\mathbf{g}}_+^\dagger\times) + \hat{\sigma}_3(ik_0\tilde{\mathbf{t}}_+\times)\right]\begin{pmatrix} \mathbf{E}_T \\ \eta_0\mathbf{H}_T \end{pmatrix} = -k_0\left[i\,\hat{\sigma}_1\,n_- + \hat{\sigma}_2\,n_+ - i\,\hat{\sigma}_3\,\chi_z\right]\begin{pmatrix} \mathbf{E}_z \\ \eta_0\mathbf{H}_z \end{pmatrix}, \tag{5}$$

with $\tilde{\mathbf{g}}_\pm = \frac{1}{2}(\tilde{\mathbf{g}}_1 \pm \tilde{\mathbf{g}}_2)$, $\tilde{\mathbf{t}}_\pm = \frac{1}{2}(\tilde{\mathbf{t}}_1 \pm \tilde{\mathbf{t}}_2)$, and $n_\pm = \frac{1}{2}(\varepsilon_z \pm \mu_z)$, where we have already imposed the in-plane duality condition $\overleftrightarrow{\varepsilon}_T = \overleftrightarrow{\mu}_T$. Substitution of $\hat{\sigma}_2(-\overleftrightarrow{\varepsilon}_T^{-1})$·Eq. (4) into $k_0\hat{\sigma}_2$·Eq. (5) yields

$$\left[\hat{\sigma}_0\left(\partial_i + ik_0\tilde{t}_{-i}\right) + ik_0\left(\hat{\sigma}_1\tilde{g}_{-i}^* + i\hat{\sigma}_2\tilde{g}_{+i}^* - \hat{\sigma}_3\tilde{t}_{+i}\right)\right]\epsilon^{zij}(\varepsilon_T^{-1})_{jk}\epsilon^{klz}\left[\hat{\sigma}_0\left(\partial_l + ik_0\tilde{t}_{-l}\right) + ik_0\left(\hat{\sigma}_1\tilde{g}_{-l} - i\hat{\sigma}_2\tilde{g}_{+l} - \hat{\sigma}_3\tilde{t}_{+l}\right)\right]\begin{pmatrix} Ez \\ \eta_0 H_z \end{pmatrix}$$
$$= k_0^2\left(\hat{\sigma}_0 n_+ + \hat{\sigma}_1\chi_z + \hat{\sigma}_3 n_-\right)\begin{pmatrix} Ez \\ \eta_0 H_z \end{pmatrix}, \qquad (\ i, j, k, l \in \{x, y\}\ ).$$

On account of $\epsilon^{zij}(\varepsilon_T^{-1})_{jk}\epsilon^{klz} = \epsilon^{ij}(\varepsilon_T^{-1})_{ij}\epsilon^{kl} = -\varepsilon_T^{ik}/\det(\overleftrightarrow{\varepsilon}_T)$ (ϵ^{ijk}, ϵ^{ij} are 3D and 2D Levi-Civita symbols), we obtain

$$\left\{\frac{1}{2}\left[\hat{\mathbf{p}} - \hat{\sigma}_0\mathbf{A} - \left(\hat{\mathcal{A}} - i\hat{\mathcal{A}}_I\right)\right]\cdot\overleftrightarrow{m}^{-1}\cdot\left[\hat{\mathbf{p}} - \hat{\sigma}_0\mathbf{A} - \left(\hat{\mathcal{A}} + i\hat{\mathcal{A}}_I\right)\right] - k_0^2\left(\hat{\sigma}_0 n_+ + \hat{\sigma}_1\chi_z + \hat{\sigma}_3 n_-\right)\right\}\begin{pmatrix} E_z \\ \eta_0 H_z \end{pmatrix} = 0, \tag{6}$$

where $\overleftrightarrow{m}^{-1} = \frac{2}{\det(\overleftrightarrow{\varepsilon}_T)}\overleftrightarrow{\varepsilon}_T$ is the inverse of an effective anisotropic mass, $\mathbf{A} = -k_0\,\tilde{\mathbf{t}}_- = k_0\,\mathbf{t}_- \times \mathbf{e}_z$ denotes an emergent Abelian vector potential, and

$$\hat{\mathcal{A}}_{(c)} = \hat{\mathcal{A}} + i\hat{\mathcal{A}}_I = \underbrace{k_0\left[\hat{\sigma}_1\mathrm{Re}(\mathbf{g}_-)\times\mathbf{e}_z + \hat{\sigma}_2\mathrm{Im}(\mathbf{g}_-)\times\mathbf{e}_z - \hat{\sigma}_3\mathbf{t}_+\times\mathbf{e}_z\right]}_{\hat{\mathcal{A}} = k_0\begin{pmatrix} -\mathbf{t}_+\times\mathbf{e}_z & \mathbf{g}_-^\dagger\times\mathbf{e}_z \\ \mathbf{g}_-\times\mathbf{e}_z & \mathbf{t}_+\times\mathbf{e}_z\end{pmatrix}} + i\underbrace{k_0\left(\hat{\sigma}_1\mathrm{Im}(\mathbf{g}_+)\times\mathbf{e}_z - \hat{\sigma}_2\mathrm{Re}(\mathbf{g}_+)\times\mathbf{e}_z\right)}_{\hat{\mathcal{A}}_I = k_0\begin{pmatrix} 0 & i\mathbf{g}_+^\dagger\times\mathbf{e}_z \\ -i\mathbf{g}_+\times\mathbf{e}_z & 0\end{pmatrix}} \tag{7}$$

can be regarded as a complex-valued non-Abelian vector potential with $\mathbf{g}_\pm = \frac{1}{2}(\mathbf{g}_1 \pm \mathbf{g}_2^*)$ and $\mathbf{t}_\pm = \frac{1}{2}(\mathbf{t}_1 \pm \mathbf{t}_2)$.

The imaginary part, $\hat{\mathcal{A}}_I$, of non-Abelian potential can be further taken out from the "kinetic energy part":

$$\begin{aligned}\Big\{&\frac{1}{2}\big(\hat{\mathbf{p}} - \hat{\mathscr{A}}\big)\cdot\overleftrightarrow{m}^{-1}\cdot\big(\hat{\mathbf{p}} - \hat{\mathscr{A}}\big) - k_0^2\big(\hat{\sigma}_0 n_+ + \hat{\sigma}_1\chi_z + \hat{\sigma}_3 n_-\big)\\ &+\frac{1}{2}\left[\hat{\mathcal{A}}_I\cdot\overleftrightarrow{m}^{-1}\cdot\big(\hat{\sigma}_0\nabla_T - i\hat{\mathscr{A}}\big) - \big(\hat{\sigma}_0\nabla_T - i\hat{\mathscr{A}}\big)\cdot\overleftrightarrow{m}^{-1}\cdot\hat{\mathcal{A}}_I + \hat{\mathcal{A}}_I\cdot\overleftrightarrow{m}^{-1}\cdot\hat{\mathcal{A}}_I\right]\Big\}\begin{pmatrix} E_z \\ \eta_0 H_z\end{pmatrix} = 0,\end{aligned} \tag{8}$$

where $\hat{\mathscr{A}} = \hat{\sigma}_0\mathsf{A} + \hat{\mathcal{A}}$ is the complete U(2) real vector potential, and the second line associated with $\hat{\mathcal{A}}_I$ can be decomposed as

$$\begin{aligned}&\hat{\mathcal{A}}_I\cdot\overleftrightarrow{m}^{-1}\cdot\big(\hat{\sigma}_0\nabla_T - i\hat{\mathscr{A}}\big) - \big(\hat{\sigma}_0\nabla_T - i\hat{\mathscr{A}}\big)\cdot\overleftrightarrow{m}^{-1}\cdot\hat{\mathcal{A}}_I + \hat{\mathcal{A}}_I\cdot\overleftrightarrow{m}^{-1}\cdot\hat{\mathcal{A}}_I\\ =& -\nabla_T\cdot\big(\overleftrightarrow{m}^{-1}\cdot\hat{\mathcal{A}}_I\big) + i\big(-\hat{\mathcal{A}}_I\cdot\overleftrightarrow{m}^{-1}\cdot\hat{\mathcal{A}} + \hat{\mathcal{A}}\cdot\overleftrightarrow{m}^{-1}\cdot\hat{\mathcal{A}}_I\big) + \hat{\mathcal{A}}_I\cdot\overleftrightarrow{m}^{-1}\cdot\hat{\mathcal{A}}_I\\ =& -\nabla_T\cdot\big(\overleftrightarrow{m}^{-1}\cdot\hat{\mathcal{A}}_I\big) + i\,\mathrm{Tr}\Big(\overleftrightarrow{m}^{-1}\cdot\big[\hat{\mathcal{A}},\hat{\mathcal{A}}_I\big]\Big) + \hat{\mathcal{A}}_I\cdot\overleftrightarrow{m}^{-1}\cdot\hat{\mathcal{A}}_I,\end{aligned} \tag{9}$$

each term of which can be further expressed with the material parameters:

$$\begin{aligned}-\nabla_T\cdot\big(\overleftrightarrow{m}^{-1}\cdot\hat{\mathcal{A}}_I\big) &= -k_0\nabla\cdot\Big\{\overleftrightarrow{m}^{-1}\cdot\Big[\big(\mathrm{Im}(\mathbf{g}_+)\hat{\sigma}_1 - \mathrm{Re}(\mathbf{g}_+)\hat{\sigma}_2\big)\times\mathbf{e}_z\Big]\Big\}\\ &= -k_0\partial_i\Big\{(\overleftrightarrow{m}^{-1})^{ij}\epsilon_{jk}\big(\mathrm{Im}(\mathbf{g}_+)\hat{\sigma}_1 - \mathrm{Re}(\mathbf{g}_+)\hat{\sigma}_2\big)^k\Big\}\\ &= -k_0\partial_i\Big\{\big(-2\epsilon^{il}(\overleftrightarrow{\varepsilon}_T^{-1})_{lm}\epsilon^{mj}\epsilon_{jk}\big)\big(\mathrm{Im}(\mathbf{g}_+)\hat{\sigma}_1 - \mathrm{Re}(\mathbf{g}_+)\hat{\sigma}_2\big)^k\Big\}\\ &= -2k_0\epsilon^{il}\partial_i\Big\{(\overleftrightarrow{\varepsilon}_T^{-1})_{lk}\big(\mathrm{Im}(\mathbf{g}_+)\hat{\sigma}_1 - \mathrm{Re}(\mathbf{g}_+)\hat{\sigma}_2\big)^k\Big\}\\ &= -2k_0\,\mathbf{e}_z\cdot\Big\{\nabla\times\Big[\overleftrightarrow{\varepsilon}_T^{-1}\cdot\big(\mathrm{Im}(\mathbf{g}_+)\hat{\sigma}_1 - \mathrm{Re}(\mathbf{g}_+)\hat{\sigma}_2\big)\Big]\Big\},\end{aligned} \tag{10a}$$

$$\begin{aligned}i\,\mathrm{Tr}\Big(\overleftrightarrow{m}^{-1}\cdot\big[\hat{\mathcal{A}},\hat{\mathcal{A}}_I\big]\Big) &= i\,(\overleftrightarrow{m}^{-1})^{ij}\mathcal{A}_i^a\mathcal{A}_{I\,j}^b\big[\hat{\sigma}_a,\hat{\sigma}_b\big] = i\,(\overleftrightarrow{m}^{-1})^{ij}\mathcal{A}_i^a\mathcal{A}_{I\,j}^b(2i\epsilon_{abc}\hat{\sigma}^c)\\ &= 4\big[(\mathcal{A}_i^a\epsilon^{il})(\overleftrightarrow{\varepsilon}_I^{-1})_{lm}(\epsilon^{mj}\mathcal{A}_{I\,j}^b)\epsilon_{abc}\big]\hat{\sigma}^c\\ &= 4k_0^2\Big[\big(\mathbf{t}_+\cdot\overleftrightarrow{\varepsilon}_T^{-1}\cdot\mathrm{Re}(\mathbf{g}_+)\big)\hat{\sigma}_1 + \big(\mathbf{t}_+\cdot\overleftrightarrow{\varepsilon}_T^{-1}\cdot\mathrm{Im}(\mathbf{g}_+)\big)\hat{\sigma}_2 + \mathrm{Re}\big(\mathbf{g}_-\cdot\overleftrightarrow{\varepsilon}_T^{-1}\cdot\mathbf{g}_+^\dagger\big)\hat{\sigma}_3\Big]\end{aligned} \tag{10b}$$

$$\begin{aligned}\hat{\mathcal{A}}_I\cdot\overleftrightarrow{m}^{-1}\cdot\hat{\mathcal{A}}_I &= k_0^2\Big[\big(\mathrm{Im}(\mathbf{g}_+)\hat{\sigma}_1 - \mathrm{Re}(\mathbf{g}_+)\hat{\sigma}_2\big)\times\mathbf{e}_z\Big]\cdot\overleftrightarrow{m}^{-1}\cdot\Big[\big(\mathrm{Im}(\mathbf{g}_+)\hat{\sigma}_1 - \mathrm{Re}(\mathbf{g}_+)\hat{\sigma}_2\big)\times\mathbf{e}_z\Big]\\ &= 2k_0^2\big(\mathrm{Im}(\mathbf{g}_+)\hat{\sigma}_1 - \mathrm{Re}(\mathbf{g}_+)\hat{\sigma}_2\big)\cdot\overleftrightarrow{\varepsilon}_T^{-1}\cdot\big(\mathrm{Im}(\mathbf{g}_+)\hat{\sigma}_1 - \mathrm{Re}(\mathbf{g}_+)\hat{\sigma}_2\big)\\ &= 2k_0^2\big(\mathrm{Im}(\mathbf{g}_+)\cdot\overleftrightarrow{\varepsilon}_T^{-1}\cdot\mathrm{Im}(\mathbf{g}_+) + \mathrm{Re}(\mathbf{g}_+)\cdot\overleftrightarrow{\varepsilon}_T^{-1}\cdot\mathrm{Re}(\mathbf{g}_+)\big)\\ &= 2k_0^2\big(\mathbf{g}_+\overleftrightarrow{\varepsilon}_T^{-1}\cdot\mathbf{g}_+^\dagger\big)\hat{\sigma}_0\end{aligned} \tag{10c}$$

Supplementary Table 1. The complete set of the components of the synthetic $U(2) = \mathrm{SU}(2) \rtimes \mathrm{U}(1)$ gauge potential corresponding to the spinor state $|\psi\rangle = (E_z, \eta_0 H_z)^{\intercal}$, where the "inner product" $\langle\!\langle\cdot,\cdot\rangle\!\rangle$ for two 2D vectors $\mathbf{a}$, $\mathbf{b}$ is defined as $\langle\!\langle\mathbf{a},\mathbf{b}\rangle\!\rangle = \mathbf{a}\cdot\overleftrightarrow{\varepsilon}_T^{-1}\cdot\mathbf{b}^{\dagger}$.

		expression	physical origin
SU(2)	vector potential $\hat{\mathcal{A}} = \mathcal{A}^a\hat{\sigma}_a$	$\mathcal{A}^1 = k_0\mathrm{Re}\left(\mathbf{g}_-\right)\times\mathbf{e}_z$	reciprocal anisotropy of $\overleftrightarrow{\varepsilon}$, $\overleftrightarrow{\mu}$ [1, 2] (deviation of principal axis from z-direction)
		$\mathcal{A}^2 = k_0\mathrm{Im}\left(\mathbf{g}_-\right)\times\mathbf{e}_z$	gyroelectric or gyromagnetic effects induced by in-plane magnetic field [3]
		$\mathcal{A}^3 = -k_0\mathbf{t}_+\times\mathbf{e}_z$	real & symmetric part of ME tensors [4] (anisotropic Tellegen media)
	scalar potential $\hat{\mathcal{A}}_0 = \mathcal{A}_0^a\hat{\sigma}_a$	$\mathcal{A}_0^1 = k_0\mathbf{e}_z\cdot\left[\nabla\times\left(\overleftrightarrow{\varepsilon}_T^{-1}\cdot\mathrm{Im}\left(\mathbf{g}_+\right)\right)\right] + k_0^2\left[\chi_z - 2\langle\!\langle\mathbf{t}_+,\mathrm{Re}(\mathbf{g}_+)\rangle\!\rangle\right]$	• inhomogeneity of $\mathbf{g}_+$ and $\overleftrightarrow{\varepsilon}_T$ • coupling between $\mathbf{g}_+$ and $\mathbf{t}_+$ • χ_z component of ME tensor
		$\mathcal{A}_0^2 = -k_0\mathbf{e}_z\cdot\left[\nabla\times\left(\overleftrightarrow{\varepsilon}_T^{-1}\cdot\mathrm{Re}\left(\mathbf{g}_+\right)\right)\right] + 2k_0^2\langle\!\langle\mathbf{t}_+,\mathrm{Im}(\mathbf{g}_+)\rangle\!\rangle$	
		$\mathcal{A}_0^3 = k_0^2\left[\dfrac{\varepsilon_z - \mu_z}{2} - 2\mathrm{Re}\langle\!\langle\mathbf{g}_-,\mathbf{g}_+\rangle\!\rangle\right]$	• difference between ε_z and μ_z • coupling between $\mathbf{g}_+$ and $\mathbf{g}_-$
U(1)	vector potential	$\mathsf{A} = k_0\,\mathbf{t}_-\times\mathbf{e}_z$	real & antisymmetric part of ME tensors (moving media or static toroidal moment [5–7])
	scalar potential	$\mathsf{V}_0 = k_0^2\left(\langle\!\langle\mathbf{g}_+,\mathbf{g}_+\rangle\!\rangle - \dfrac{\varepsilon_z+\mu_z}{2}\right)$	$\mathbf{g}_+$, ε_z, μ_z

After substituting Eqs. (9,10) into Eq. (8), we arrive at the final form of the in-plane wave equation [1]

$$\hat{H}|\psi\rangle = \left[\frac{1}{2}\left(\hat{\mathbf{p}} - \hat{\mathscr{A}}\right)\cdot\overleftrightarrow{m}^{-1}\cdot\left(\hat{\mathbf{p}} - \hat{\mathscr{A}}\right) - \hat{\mathscr{A}}_0\right]|\psi\rangle = \left[\frac{1}{2}\left(\hat{\mathbf{p}} - \mathsf{A}\hat{\sigma}_0 - \hat{\mathcal{A}}\right)\cdot\overleftrightarrow{m}^{-1}\cdot\left(\hat{\mathbf{p}} - \mathsf{A}\hat{\sigma}_0 - \hat{\mathcal{A}}\right) - \hat{\mathcal{A}}_0 + \mathsf{V}_0\hat{\sigma}_0\right]|\psi\rangle = 0. \tag{11}$$

Here, the effective Hamiltonian $\hat{H}$ is precisely like that of a non-relativistic spin-1/2 particle traveling in a $U(2) = \mathrm{SU}(2)\rtimes\mathrm{U}(1)$ background gauge potential $\{\hat{\mathscr{A}}_\mu\}$, where "$\rtimes$" denotes the semidirect product of two groups. The effective U(2) group potential always can be decomposed into two parts $\{\hat{\mathscr{A}}_\mu\} = \{\mathsf{A}_\mu\hat{\sigma}_0 + \hat{\mathcal{A}}_\mu\}$, where $\{\mathsf{A}_\mu\} = \{-\mathsf{V}_0, \mathsf{A}\}$ ($\mu = 0,1,2$) denotes an effective Abelian U(1) Maxwell-type gauge potential, while $\{\hat{\mathcal{A}}_\mu\} = \{\hat{\mathcal{A}}_0, \hat{\mathcal{A}}\}$ denotes an effective non-Abelian SU(2) Yang-Mills gauge potential ($\hat{\mathcal{A}}_\mu = \mathcal{A}_\mu^a\hat{\sigma}_a$ are $\mathbf{su}(2)$-Lie-Algebra-valued, namely they are 2×2 traceless Hermitian matrices). Their expressions are listed in Supplementary Table 1. And the results in the main text correspond to the reduced case with $\mathbf{t}_\pm = 0$ and $\chi_z = 0$.

As shown in Supplementary Table 1, different $\hat{\sigma}$-components of both vector and scalar potentials have distinct physical origins. For the SU(2) vector potential, $\mathcal{A}^1$ is caused by the the deviation of the principal axis of $\mathrm{Re}(\overleftrightarrow{\varepsilon})$, $\mathrm{Re}(\overleftrightarrow{\mu})$ from z-direction, which can be realized in reciprocal anisotropic materials [1, 2]. $\mathcal{A}^2$ component stems from the the imaginary part of the off-block-diagonal term $\mathrm{Im}(\mathbf{g}_-)$ in $\overleftrightarrow{\varepsilon}$, $\overleftrightarrow{\mu}$, which can be excited by in-plane magnetic field in magneto-optic media [3]. And $\mathcal{A}^3$ originates from the symmetric off-diagonal part of ME coupling $\mathbf{t}_+$, which, together with χ_z, actually denotes an anisotropic Tellegen media with the ME tensor $\tilde{\chi}_{em} = \tilde{\chi}_{me}^{\intercal} = \mathrm{diag}\left(\frac{1}{2}(\chi_z + \sqrt{4|\mathbf{t}_+|^2 + \chi_z^2}), \frac{1}{2}(\chi_z - \sqrt{4|\mathbf{t}_+|^2 + \chi_z^2}), 0\right)$ in the principal frame [4].

The U(2) gauge potential can induce an emergent non-Abelian U(2) gauge field acting on the spinor wave function, in terms of the covariant derivative operator $\hat{\mathscr{D}}_i = \partial_\mu\hat{\sigma}_0 - i\hat{\mathscr{A}}_\mu = \partial_\mu\hat{\sigma}_0 - i\hat{\mathcal{A}}_\mu - \mathsf{A}_\mu\hat{\sigma}_0$:

$$\hat{\mathscr{F}}_{\mu\nu} = i[\hat{\mathscr{D}}_\mu, \hat{\mathscr{D}}_\nu] = \partial_\mu\hat{\mathscr{A}}_\nu - \partial_\nu\hat{\mathscr{A}}_\mu - i[\hat{\mathscr{A}}_\mu, \hat{\mathscr{A}}_\nu] = \underbrace{\left(\partial_\mu\mathsf{A}_\nu - \partial_\nu\mathsf{A}_\mu\right)}_{\mathrm{U(1)\ field:}\ \mathsf{F}_{\mu\nu}}\hat{\sigma}_0 + \underbrace{\partial_\mu\hat{\mathcal{A}}_\nu - \partial_\nu\hat{\mathcal{A}}_\mu - i[\hat{\mathcal{A}}_\mu, \hat{\mathcal{A}}_\nu]}_{\mathrm{SU(2)\ field:}\ \hat{\mathcal{F}}_{\mu\nu} = i[\hat{\mathcal{D}}_\mu, \hat{\mathcal{D}}_\nu]}, \tag{12}$$

[1] For a fixed frequency, Eq. (11) is analogous to the stationary Schrodinger equation: $(\hat{H} - E)|\psi\rangle = 0$. As the zero point of the U(1) scalar potential V_0 is arbitrary, it can always be selected such that $E = 0$. The free choice of E will not affect the stationary dynamics, but it is meaningless to compare the eigen-energy for the effective Hamiltonian at different frequencies.

where $\hat{\mathcal{D}}_\mu = \partial_\mu \hat{\sigma}_0 - i\hat{\mathcal{A}}_\mu$. As shown, the U(2) gauge field also can be decomposed into a U(1) part and a SU(2) part. Since the effective SU(2) gauge field is $\mathbf{su}(2)$-Lie-algebra-valued, and also can be expanded by Pauli matrices, $\hat{\mathcal{F}}_{\mu\nu} = \mathcal{F}^a_{\mu\nu}\hat{\sigma}_a$, the SU(2) part of Eq. (12) can be rewritten in a component form:

$$\mathcal{F}^a_{\mu\nu} = \partial_\mu \mathcal{A}^a_\nu - \partial_\nu \mathcal{A}^a_\mu + 2\epsilon^a{}_{bc}\mathcal{A}^b_\mu \mathcal{A}^c_\nu, \tag{13}$$

where $2\epsilon_{abc}$ is the structure constant of $\mathbf{su}(2)$ Lie algebra, $[\hat{\sigma}_a, \hat{\sigma}_b] = 2i\epsilon_{abc}\hat{\sigma}^c$.

Analogous to the real EM field, the effective U(1) gauge field $\mathsf{F}_{\mu\nu}$ can be alternatively treated as a pair of effective Abelian magnetic and electric fields B, E (not to be confused with the real EM fields $\mathbf{B}$, $\mathbf{E}$):

$$\mathsf{B} = \nabla \times \mathsf{A} = -\mathbf{e}_z (\nabla \cdot \mathbf{t}_-), \quad \mathsf{E} = -\nabla \mathsf{V}_0, \tag{14}$$

Similarly, if we express the tensor of the SU(2) non-Abelian field as

$$\left(\hat{\mathcal{F}}_{\mu\nu}\right) = \begin{pmatrix} 0 & -\hat{\mathcal{E}}_x & -\hat{\mathcal{E}}_y \\ \hat{\mathcal{E}}_x & 0 & \hat{\mathcal{B}}_z \\ \hat{\mathcal{E}}_y & -\hat{\mathcal{B}}_z & 0 \end{pmatrix}, \tag{15}$$

different components of the SU(2) gauge field also can be classified into an effective non-Abelian magnetic field $\hat{\mathcal{B}}$ and an effective non-Abelian electric field $\hat{\mathcal{E}}$ separately:

$$\hat{\mathcal{B}} = \frac{1}{2}\epsilon^{ij}\hat{\mathcal{F}}_{ij}\mathbf{e}_z = \nabla \times \hat{\mathcal{A}} - i\hat{\mathcal{A}} \times \hat{\mathcal{A}}, \qquad \hat{\mathcal{E}} = -\hat{\mathcal{F}}_{0i}\mathbf{e}_i = \nabla \hat{\mathcal{A}}_0 + i[\hat{\mathcal{A}}_0, \hat{\mathcal{A}}]. \tag{16}$$

As the system is z-invariant, the effective magnetic fields B, $\hat{\mathcal{B}}$ are along z direction, while the effective electric fields E, $\hat{\mathcal{E}}$ always lie in xy-plane. We will show that these effective non-Abelian SU(2) magnetic and electric fields can affect the centroid motion of the spinor wave function $|\psi\rangle$ in a similar way as real EM fields acting on charged particles. According to Supplementary Table **1**, the SU(2) vector potential $\hat{\mathcal{A}}$ only depends on $\mathbf{g}_-$ and $\mathbf{t}_+$. Hence, the SU(2) magnetic field has a relatively simple expression depending on $\mathbf{g}_-$ and $\mathbf{t}_+$:

$$\hat{\mathcal{B}} = k_0^2\Big[2\big(\mathbf{t}_+ \times \mathrm{Im}(\mathbf{g}_-)\big)\hat{\sigma}_1 - 2\big(\mathbf{t}_+ \times \mathrm{Re}(\mathbf{g}_-)\big)\hat{\sigma}_2 + i\left(\mathbf{g}_- \times \mathbf{g}_-^*\right)\hat{\sigma}_3\Big] - k_0\nabla \cdot \left[\mathrm{Re}\left(\mathbf{g}_-\right)\hat{\sigma}_1 + \mathrm{Im}\left(\mathbf{g}_-\right)\hat{\sigma}_2 - \mathbf{t}_+\hat{\sigma}_3\right]\mathbf{e}_z. \tag{17}$$

By contrast, the SU(2) electric field $\hat{\mathcal{E}}$, is determined by all the following components: $\mathbf{g}_\pm$, $\mathbf{t}_+$, ε_z, μ_z, and $\overleftrightarrow{\varepsilon}_T$.

Supplementary Note 2. Gauge transformation between synthetic gauge field systems

Since the choice of the gauge of the wave function can be arbitrary, we can consider a gauge transformation of the wave function $|\psi'\rangle = \hat{U}(\mathbf{r})|\psi\rangle$, where $\hat{U}(\mathbf{r}) = \hat{U}(x,y)$ is now generalized to be a xy-dependent U(2) matrix. The wave equation is covariant under the gauge transformation: $\hat{U}\hat{H}|\psi\rangle = (\hat{U}\hat{H}\hat{U}^\dagger)|\psi'\rangle = 0$ with the transformed Hamiltonian

$$\hat{H}' = \hat{U}\hat{H}\hat{U}^\dagger = -\frac{1}{2}\left(\hat{U}\hat{\mathscr{D}}_i\hat{U}^\dagger\right)\left(m^{-1}\right)^{ij}\left(\hat{U}\hat{\mathscr{D}}_j\hat{U}^\dagger\right) - \left(\hat{U}\hat{\mathscr{A}}_0\hat{U}^\dagger\right) = \frac{1}{2}\hat{\mathscr{D}}_i'\left(m^{-1}\right)^{ij}\hat{\mathscr{D}}_j' - \hat{\mathscr{A}}_0', \tag{18}$$

and the transformed covariant derivative satisfies

$$\hat{\mathscr{D}}_i' = \hat{U}\hat{\mathscr{D}}_i\hat{U}^\dagger = \partial_i\hat{\sigma}_0 - i\left(\hat{U}\hat{\mathscr{A}}_i\hat{U}^\dagger + i\hat{U}\partial_i\hat{U}^\dagger\right) = \partial_i\hat{\sigma}_0 - i\hat{\mathscr{A}}_i'. \tag{19}$$

As a result, we obtain the rule of gauge transformation for the gauge potential:

$$\left.\begin{aligned}\hat{\mathscr{A}}_i' &= \hat{U}\hat{\mathscr{A}}_i\hat{U}^\dagger + i\hat{U}\partial_i\hat{U}^\dagger\\ \hat{\mathscr{A}}_0' &= \hat{U}\hat{\mathscr{A}}_0\hat{U}^\dagger = \hat{U}\hat{\mathscr{A}}_0\hat{U}^\dagger + \underbrace{i\hat{U}\partial_0\hat{U}^\dagger}_{=0}\end{aligned}\right\} \quad \hat{\mathscr{A}}_\mu' = \hat{U}\hat{\mathscr{A}}_\mu\hat{U}^\dagger + i\hat{U}\partial_\mu\hat{U}^\dagger. \tag{20}$$

In addition, since the 2×2 unitary matrix can be expressed generically as $\hat{U} = e^{i\varphi}\hat{\mathcal{U}}$ with a U(1) part $e^{i\varphi}$ and a SU(2) part $\hat{\mathcal{U}}$ satisfying $\det(\hat{\mathcal{U}}) = 1$, we have $i\hat{U}\partial_\mu\hat{U}^\dagger = -\partial_\mu\varphi\hat{\sigma}_0 + i\hat{\mathcal{U}}\partial_\mu\hat{\mathcal{U}}^\dagger$. And $\mathrm{Tr}\left(i\hat{\mathcal{U}}\partial_\mu\hat{\mathcal{U}}^\dagger\right) = i\det(\hat{\mathcal{U}})\partial_\mu\det(\hat{\mathcal{U}}^\dagger) \equiv 0$, hence $i\hat{\mathcal{U}}\partial_\mu\hat{\mathcal{U}}^\dagger \in \mathbf{su}(2)$, the gauge transformation of U(1) and SU(2) parts of the gauge potential obeys

$$\mathsf{A}_\mu' = \mathsf{A}_\mu - \partial_\mu\varphi, \qquad \hat{\mathcal{A}}_\mu' = \hat{\mathcal{U}}\hat{\mathcal{A}}_\mu\hat{\mathcal{U}}^\dagger + i\hat{\mathcal{U}}\partial_\mu\hat{\mathcal{U}}^\dagger. \tag{21}$$

Meanwhile, the gauge transformation of gauge fields reads

$$\hat{\mathscr{F}}_{\mu\nu}' = i[\hat{\mathscr{D}}_\mu', \hat{\mathscr{D}}_\nu'] = \hat{U}\hat{\mathscr{F}}_{\mu\nu}\hat{U}^\dagger \quad \begin{cases}\mathrm{U(1)}: & \hat{\mathsf{F}}_{\mu\nu}' = \hat{\mathsf{F}}_{\mu\nu} \quad \Leftrightarrow \quad \hat{\mathsf{B}}' = \hat{\mathsf{B}}, \quad \hat{\mathsf{E}}' = \hat{\mathsf{E}}\\ \mathrm{SU(2)}: & \hat{\mathcal{F}}_{\mu\nu}' = \hat{\mathcal{U}}\hat{\mathcal{F}}_{\mu\nu}\hat{\mathcal{U}}^\dagger \quad \Leftrightarrow \quad \hat{\mathcal{B}}' = \hat{\mathcal{U}}\hat{\mathcal{B}}\hat{\mathcal{U}}^\dagger, \quad \hat{\mathcal{E}}' = \hat{\mathcal{U}}\hat{\mathcal{E}}\hat{\mathcal{U}}^\dagger\end{cases} \tag{22}$$

Unlike the U(1) field which is independent of the gauge choice, the SU(2) part of the gauge field is gauge-dependent.

It should be noted that the meaning of gauge covariance in the synthetic gauge system is subtly different from that in real gauge systems. For real gauge fields, the choice of gauge refers to the process of regulating the excess unphysical degrees of freedom, hence the gauge transformation leaves all observables unaffected, while all of the gauge-dependent quantities cannot be directly observed. However, in the synthetic gauge system, different gauges correspond to different materials, and the gauge dependence of the effective wave function $|\psi\rangle = (E_z, \eta_0 H_z)^\top$ can be directly detected, since the EM fields, including their phases, are measurable. According to the discussions in the Methods of the main text, the gauge transformation acting on the wave function $|\psi'\rangle = \hat{U}(\mathbf{r})|\psi\rangle$ gives rise to the transformation of the total EM fields as

$$\Psi' = \tilde{U}(\mathbf{r})\Psi = \begin{pmatrix}\hat{\sigma}_2\hat{U}(\mathbf{r})\hat{\sigma}_2 & 0\\ 0 & \hat{U}(\mathbf{r})\end{pmatrix}\Psi, \tag{23}$$

with $\Psi = (\mathbf{E}_T, \eta_0\mathbf{H}_T, E_z, \eta_0 H_z)^\top$. Considering the 2D Maxwell's equations expressed in terms of Ψ:

$$\underbrace{\left(\begin{array}{c|c}0 & i\hat{\sigma}_2(i\nabla_T\times)\mathbf{e}_z\\ \hline i\hat{\sigma}_2\,\mathbf{e}_z\cdot(i\nabla_T\times) & 0\end{array}\right)}_{\mathcal{M}}\Psi = k_0\underbrace{\left(\begin{array}{cc|cc}\overleftrightarrow{\varepsilon}_T & 0 & \mathbf{g}_1 & \mathbf{t}_1/c\\ 0 & \overleftrightarrow{\varepsilon}_T & \mathbf{t}_2/c & \mathbf{g}_2\\ \hline \mathbf{g}_1^\dagger & \mathbf{t}_2^\top/c & \varepsilon_z & \chi_z\\ \mathbf{t}_1^\top/c & \mathbf{g}_2^\dagger & \chi_z & \mu_z\end{array}\right)}_{\mathcal{N}}\Psi, \tag{24}$$

the gauge transformation of Maxwell's equations shows that

$$(\tilde{U}\mathcal{M}\tilde{U}^\dagger)\Psi' = (\mathcal{M} + \Delta\mathcal{M})\Psi' = k_0(\tilde{U}\mathcal{N}\tilde{U}^\dagger)\Psi', \tag{25}$$

where

$$\Delta\mathcal{M} = \left(\begin{array}{c|c} 0 & i\hat{\sigma}_2(i\hat{U}\nabla_T\hat{U}^\dagger)\times\mathbf{e}_z \\ \hline -i(i\hat{U}\nabla_T\hat{U}^\dagger)\times\mathbf{e}_z\hat{\sigma}_2 & 0 \end{array}\right). \tag{26}$$

Then we find that Ψ' satisfies the Maxwell's equations $\mathcal{M}\Psi' = k_0\mathcal{N}'\Psi'$ in the transformed material:

$$\mathcal{N}' = \tilde{U}\mathcal{N}\tilde{U}^\dagger - \Delta\mathcal{M}/k_0 \quad \begin{cases} \overleftrightarrow{\varepsilon}'_T = \overleftrightarrow{\varepsilon}_T \\ \begin{pmatrix}\varepsilon'_z & \chi'_z \\ \chi'_z & \mu'_z\end{pmatrix} = \hat{U}\begin{pmatrix}\varepsilon_z & \chi_z \\ \chi_z & \mu_z\end{pmatrix}\hat{U}^\dagger \\ \begin{pmatrix}\mathbf{t}'_2/c & \mathbf{g}'_2 \\ -\mathbf{g}'_1 & -\mathbf{t}'_1/c\end{pmatrix} = \hat{U}\begin{pmatrix}\mathbf{t}_2/c & \mathbf{g}_2 \\ -\mathbf{g}_1 & -\mathbf{t}_1/c\end{pmatrix}\hat{U}^\dagger + \frac{1}{k_0}(i\hat{U}\nabla_T\hat{U}^\dagger)\times\mathbf{e}_z, \end{cases} \tag{27}$$

moreover, the synthetic U(2) gauge potential in this transformed material is consistent with the result given by the gauge transformation Eqs. (20,21).

To be precise, the gauge covariance of the synthetic gauge system means that if the material parameters in two systems can be related according to the gauge transformation Eq. (27), the solutions of EM fields in the two systems have a one-to-one correspondence in terms of the mapping Eq. (23), and the forms of synthetic gauge potentials and fields in the two systems are transformed in exactly the same way as the real non-Abelian gauge potentials and fields expressed in different gauges. Moreover, all of the gauge-independent quantities in real gauge systems, such as the effective probability density $|\psi(\mathbf{r})|^2$, the quadratic forms of gauge fields $\mathrm{Tr}\left(\hat{\mathcal{F}}_{\alpha\beta}\hat{\mathcal{F}}_{\mu\nu}\right)$, and the Wilson loop, are also identical in the two synthetic gauge systems which are related by the material and field transformations. Especially, the time-averaged in-plane Poynting vectors for the field Ψ' in the transformed medium is also identical with that in the original medium:

$$\begin{aligned} \bar{\mathbf{S}}'_T &= \frac{1}{2}\mathrm{Re}\left[(\mathbf{E}'^*_z, \mathbf{H}'^*_z)\left(i\hat{\sigma}_2\otimes(\overleftrightarrow{I}\times\overleftrightarrow{I})\right)\begin{pmatrix}\mathbf{E}'_T \\ \mathbf{H}'_T\end{pmatrix}\right] \\ &= \frac{1}{2}\mathrm{Re}\left[(\mathbf{E}^*_z, \mathbf{H}^*_z)(\hat{U}^\dagger\otimes\overleftrightarrow{I})\left(i\hat{\sigma}_2\otimes(\overleftrightarrow{I}\times\overleftrightarrow{I})\right)\left((\hat{\sigma}_2\hat{U}\hat{\sigma}_2)\otimes\overleftrightarrow{I}\right)\begin{pmatrix}\mathbf{E}_T \\ \mathbf{H}_T\end{pmatrix}\right] \\ &= \frac{1}{2}\mathrm{Re}\left[(\mathbf{E}^*_z, \mathbf{H}^*_z)\left(i\hat{\sigma}_2\otimes(\overleftrightarrow{I}\times\overleftrightarrow{I})\right)\begin{pmatrix}\mathbf{E}_T \\ \mathbf{H}_T\end{pmatrix}\right] = \bar{\mathbf{S}}_T. \end{aligned} \tag{28}$$

This result indicates that we can design material parameters using non-Abelian gauge transformation to manipulate the spin (polarization) of light without changing its flow.

Supplementary Note 3. Analogy with *Zitterbewegung* effect arising from Dirac cone

The original ZB effect for Dirac electrons stems from the superposition of positive and negative energy states [8]. And a majority of previous photonic realizations of ZB are based on a Dirac cone dispersion. By contrast, the appearance of ZB effect in our system does not rely on a Dirac cone dispersion. In this section, we show that, in some limiting situations, the ZB effect in our system can also be understood from a Dirac cone structure.

Consider the transition from a reduced Abelian medium ($\hat{\mathcal{A}} = \mathcal{A}_y^2\,\mathbf{e}_y\hat{\sigma}_2, = k_0\tilde{\mathcal{A}}_y^2\,\mathbf{e}_y\hat{\sigma}_2,\ \hat{\mathcal{A}}_0 = 0$) to a non-Abelian medium ($\hat{\mathcal{A}} = k_0\tilde{\mathcal{A}}_y^2\,\mathbf{e}_y\hat{\sigma}_2 + k_0\tilde{\mathcal{A}}_x^1\,\mathbf{e}_x\hat{\sigma}_1,\ \hat{\mathcal{A}}_0 = 0$, i.e. example I in the main text):

$$\begin{array}{ccc}\text{Abelian medium} & & \text{non-Abelian medium}\\ \overleftrightarrow{\varepsilon}/\varepsilon_0 = \overleftrightarrow{\mu}/\mu_0 = \left(\begin{array}{cc|c}\varepsilon_T & 0 & -i\tilde{\mathcal{A}}_y^2\\ 0 & \varepsilon_T & 0\\ \hline i\tilde{\mathcal{A}}_y^2 & 0 & \varepsilon_z\end{array}\right), & \Rightarrow & \left\{\begin{array}{l}\overleftrightarrow{\varepsilon}/\varepsilon_0 = \left(\begin{array}{cc|c}\varepsilon_T & 0 & -i\tilde{\mathcal{A}}_y^2\\ 0 & \varepsilon_T & \tilde{\mathcal{A}}_x^1\\ \hline i\tilde{\mathcal{A}}_y^2 & \tilde{\mathcal{A}}_x^1 & \varepsilon_z\end{array}\right),\\ \overleftrightarrow{\mu}/\mu_0 = \left(\begin{array}{cc|c}\varepsilon_T & 0 & -i\tilde{\mathcal{A}}_y^2\\ 0 & \varepsilon_T & -\tilde{\mathcal{A}}_x^1\\ \hline i\tilde{\mathcal{A}}_y^2 & -\tilde{\mathcal{A}}_x^1 & \varepsilon_z\end{array}\right).\end{array}\right.\end{array} \tag{29}$$

In the Abelian medium, the sole nonzero component of the SU(2) gauge potential is $\mathcal{A}^2 = k_0\tilde{\mathcal{A}}_y^2\mathbf{e}_y$, the effective Hamiltonian has U(1) spin rotation symmetry about the $\hat{\sigma}_2$-axis, thus the two eigenmodes are $\psi_\pm = (1, \pm i)^\top$ (i.e. $E_z \pm i\eta_o H_z$) whose pseudo-spins are uniformly polarized along the $\hat{\sigma}_2$-axis for an arbitrary direction of the wave vector. The two branches of isofrequency contours are degenerate at $\mathbf{k} = k\,\mathbf{e}_x = \sqrt{\varepsilon_T\varepsilon_z - (\tilde{\mathcal{A}}_y^2)^2}\,k_0$. In the vicinity of

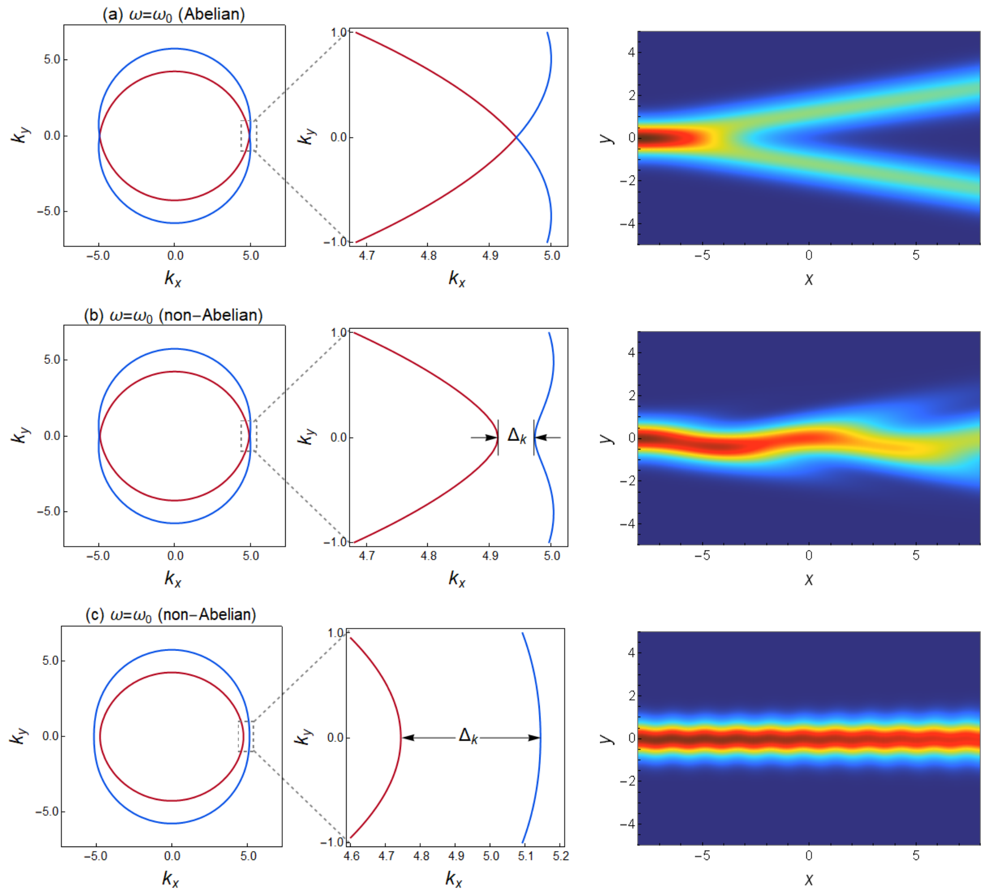

Supplementary Figure 1. Transition from a Abelian medium to a non-Abelian medium corresponding to Eq. (29). (a) Reduced Abelian gauge field medium with $\mathcal{A}_y^2 = 0.15k_0$; (b) Non-Abelian perturbed medium with $\mathcal{A}_y^2 = 0.15k_0$ and a perturbation $\mathcal{A}_x^1 = 0.006k_0$; (c) Non-Abelian medium with $\mathcal{A}_y^2 = 0.15k_0$ and $\mathcal{A}_x^1 = 0.04k_0$. Other parameters of the three media are identical: $\varepsilon_T = \varepsilon_z = 1$.

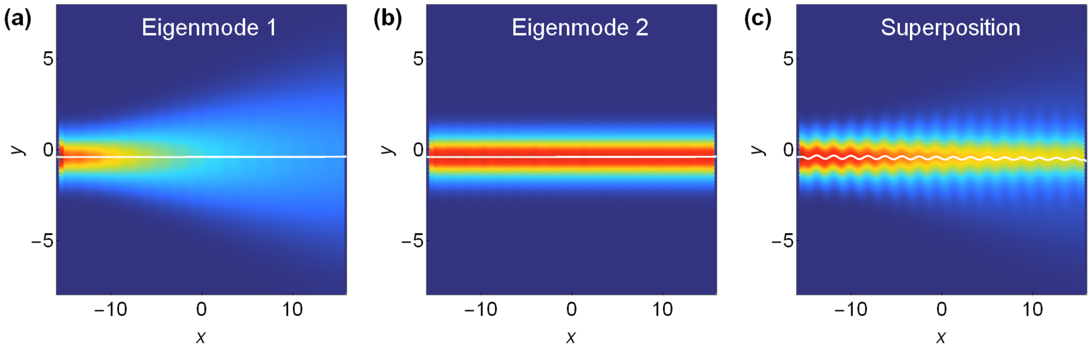

Supplementary Figure 2. Spatial ZB of monochromatic beam arising from eigenmode superposition. (a,b) Beams with eigen-polarizations travel in straight lines. (c) A beam superposed by the two eigenfields shown in (a,b) experiences a wavy centroid trajectory (white curve). The parameters of the background non-Abelian medium are given by $\varepsilon_T = 1$, $\varepsilon_z = 1.2$, $\mu_z = 0.8$, $\mathbf{g}_1 = -\mathbf{g}_2 = 0.3\mathbf{e}_x$.

the degenerate point, the two isofrequency contours intersect linearly, and thus can be regarded as a 1D Dirac cone as shown in Supplementary Figure 1(a), provided that the x axis is treated as the pseudo-time dimension.

When the component $\mathcal{A}^1 = \mathcal{A}_x^1 \mathbf{e}_x$ emerges, the medium turns into non-Abelian characterized by the nonzero non-Abelian magnetic field $\hat{\mathcal{B}} = 2\mathcal{A}^1 \times \mathcal{A}^2 \hat{\sigma}_3 = \mathcal{A}_x^1 \mathcal{A}_y^2 \mathbf{e}_z \hat{\sigma}_3$. Meanwhile, the previous intersected isofrequency contours become fully gapped. As long as $\mathcal{A}_x^1 \ll \mathcal{A}_y^2$, the isofrequency contours nearby $\mathbf{k} = k\,\mathbf{e}_x$ can be regarded as a 1D gapped Dirac cone as shown in Supplementary Figure 1(b). And the width of the gap, i.e. the beat wave number corresponds to twice the effective mass of the 1D Dirac electron M_e:

$$\Delta_k = \left|k_+ - k_-\right| = 2|\mathcal{A}_x^1| = 2M_e. \tag{30}$$

According to the standard ZB effect of realistic electrons [8], the superposition of two eigenmodes at $\mathbf{k}$ (positive and negative energy states) will give rise to a trembling motion whose frequency is exactly determined by effective Dirac mass $2M_e = \Delta_k$. This intuitive explanation is consistent with our analytical result in Eq. (15) of the main text.

For broader scenarios, the non-Abelian media cannot be treated as the perturbation of an Abelian system, and the dispersion does not correspond to a gapped Dirac cone either, as illustrated in Supplementary Figure 1(c). However, the ZB effect, arising from the beating of two eigenmodes, still can be observed and is quantitatively in agreement with our analytical result, as we have been demonstrated with examples in the main text. In Supplementary Figure 2, we visualize the ZB effect as the consequence of beating between the two eigenmodes. In Supplementary Figure 2(a,b), two beams incident from x-direction are polarized along the two eigen-polarizations on the $k_y = 0$ cross section respectively, and they propagate in straight lines. However, when we superpose the fields of the two eigen-polarized beams, the obtained beam undergoes an obvious trembling motion as shown in Supplementary Figure 2(c).

Comparison with k-space Lorentz force induced by Berry curvature

It is worthwhile to mention that an optical wave packet propagating in **inhomogeneous** and weakly anisotropic media will also experience a virtual non-Abelian Lorentz force $\left(\hat{\Omega}_{\mathbf{k}} \times \frac{d}{d\tau}\hat{\mathbf{k}}\right)$ in the momentum $\mathbf{k}$-space induced by the non-Abelian Berry curvature $\hat{\Omega}(\mathbf{k})$ [9–11]. However, the existence of $\mathbf{k}$-space Lorentz force relies on the inhomogeneity of the media, while our non-Abelian Lorentz force in the real space can be generated purely from the anisotropy of the media, they are accordingly distinct from each other. In homogeneous media, the wave vector of a wave packet is conserved, i.e. $\frac{d}{d\tau}\hat{\mathbf{k}} = 0$, hence the $\mathbf{k}$-space Lorentz force vanishes $\left(\hat{\Omega}_{\mathbf{k}} \times \frac{d}{d\tau}\hat{\mathbf{k}}\right) \equiv 0$; whereas the SU(2) non-Abelian gauge fields in real space can still exist $\hat{\mathcal{F}}_{\mu\nu} = -i[\hat{\mathcal{A}}_\mu, \hat{\mathcal{A}}_\nu] \neq 0$ and so does the real-space Lorentz force, $\frac{1}{2}\left(\hat{\mathbf{v}} \times \hat{\mathcal{B}} - \hat{\mathcal{B}} \times \hat{\mathbf{v}}\right) + \hat{\mathcal{E}}$.

Another difference between the real-space and the $\mathbf{k}$-space schemes is that the real-space scheme is applicable for any monochromatic full-wave phenomena, as the wave equation (2) shown in the main text duplicates exactly the stationary Schrödinger equation for spin-1/2 particles interacting with background SU(2) gauge fields, while the $\mathbf{k}$-space scheme is limited to geometric optics approximation [9–11].

Supplementary Note 4. ZB effect of 3D beams with finite width in z direction

In our theory, the EM fields are supposed to be translationally invariant along the z-axis. In realistic systems, optical beams should have a finite width in the z-direction. In this section, we test whether our theory can be applied to describing real 3D beams.

If the field of a 3D beam has a Gaussian-like distribution in the z-direction: $\mathbf{E} \sim \exp\left(-(z-z_0)^2/w_z^2\right)$, its derivative $\partial_z \mathbf{E} \sim -\frac{2}{w_z^2}(z-z_0)\exp\left(-(z-z_0)^2/w_z^2\right)$ tends to zero in the middle section $z = z_0$. Thus we expect that the middle section $z = z_0$ would be a feasible 2D domain where the 2D theory of non-Abelian gauge field optics proves a good description. To substantiate our analysis, we examined the two types of non-Abelian media studied in the main text.

As for the gyrotropic material with $\overleftrightarrow{\varepsilon}_T = \overleftrightarrow{\mu}_T$, $\varepsilon_z = \mu_z$, $\mathbf{g}_1 = -\mathbf{g}_2^*$, it can exert a synthetic non-Abelian magnetic field on the light beams propagating in x-direction. As shown in Supplementary Figure 3, we numerically simulated the propagation a 3D Gaussian type beam in this medium. According to Supplementary Figure 3(c), the beam propagates strictly along the horizontal plane since the isofrequency contours are symmetric with respect to the $k_z = 0$ plane (see Supplementary Figure 3(d)). Therefore, the vertical center of the beam is fixed at $z = 0$ and the ansatz $\partial_z \mathbf{E}(z=0) \approx 0$ is always satisfied. Consequently, the 2D centroid trajectory extracted from the fields falling on the middle plane $z = 0$ perfectly matches the analytical result predicted by the 2D theory as shown in Supplementary Figure 3(e).

For the biaxial dielectric material with misaligned principal axes, it performs as a background non-Abelian electric field acting on beams propagating in the xy-plane. We simulated three 3D beams with different waists in the z-direction, namely $w_z = 3\lambda_0$, $6\lambda_0$, $8\lambda_0$, traveling in the biaxial medium. The simulation results reveal that the beams will split into two branches in the z-direction after propagating a distance as shown in Supplementary Figure 4(c,f,i). The splitting effect originates from the unparallelism of the group velocities of the two eigenmodes. According to the isofrequency contours of the medium in the $k_y = 0$ plane in Supplementary Figure 4(j), the group velocity of eigenmode $|\uparrow\rangle$ is always along the x-axis, while the group velocity of eigenmode $|\downarrow\rangle$ has a nonzero z-component. Accordingly, the vertical center of $|\downarrow\rangle$ branch moves along the z-axis. Nevertheless, the numerical centroid trajectories extracted from the $z = z_0$ section shown in Supplementary Figure 4(k) are still fairly consistent with the 2D theory in the superposed region of the two eigenmodes, they deviate from the analytical curve only when the two eigenmodes split away in the z-direction. In particular, it shows that the superposed region increases with wider beam waist w_z. Therefore, the 2D theory is even applicable for beams whose centers are not confined on the $z = z_0$ plane in a considerable large region, if the beam width w_z is wide enough.

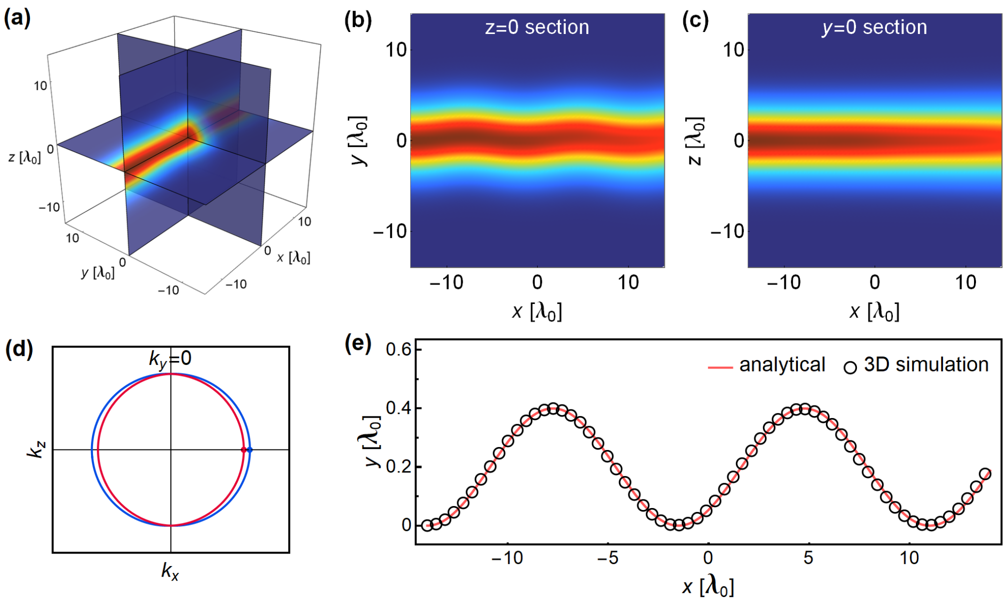

Supplementary Figure 3. ZB effect of a 3D beam with finite widths in both y and z directions in a gyrotropic medium with the parameters $\overleftrightarrow{\varepsilon}_T = \overleftrightarrow{\mu}_T = \overleftrightarrow{I}_{2\times 2}$, $\varepsilon_z = \mu_z = 1$, $\mathbf{g}_1 = -\mathbf{g}_2^* = (i\,0.1,\ 0.04)^\top$. The beam waists in y and z directions are identical $w_y = w_z = 5\lambda_0$. (a) Slice view and (b,c) section views of full-wave simulated intensity distribution of the 3D beam. (d) Isofrequency contours of the medium in the xz-plane. (e) Centroid trajectories of the 2D theory (red curve) and of the numerical fields on $z = 0$ section (black circles) shown in (b).

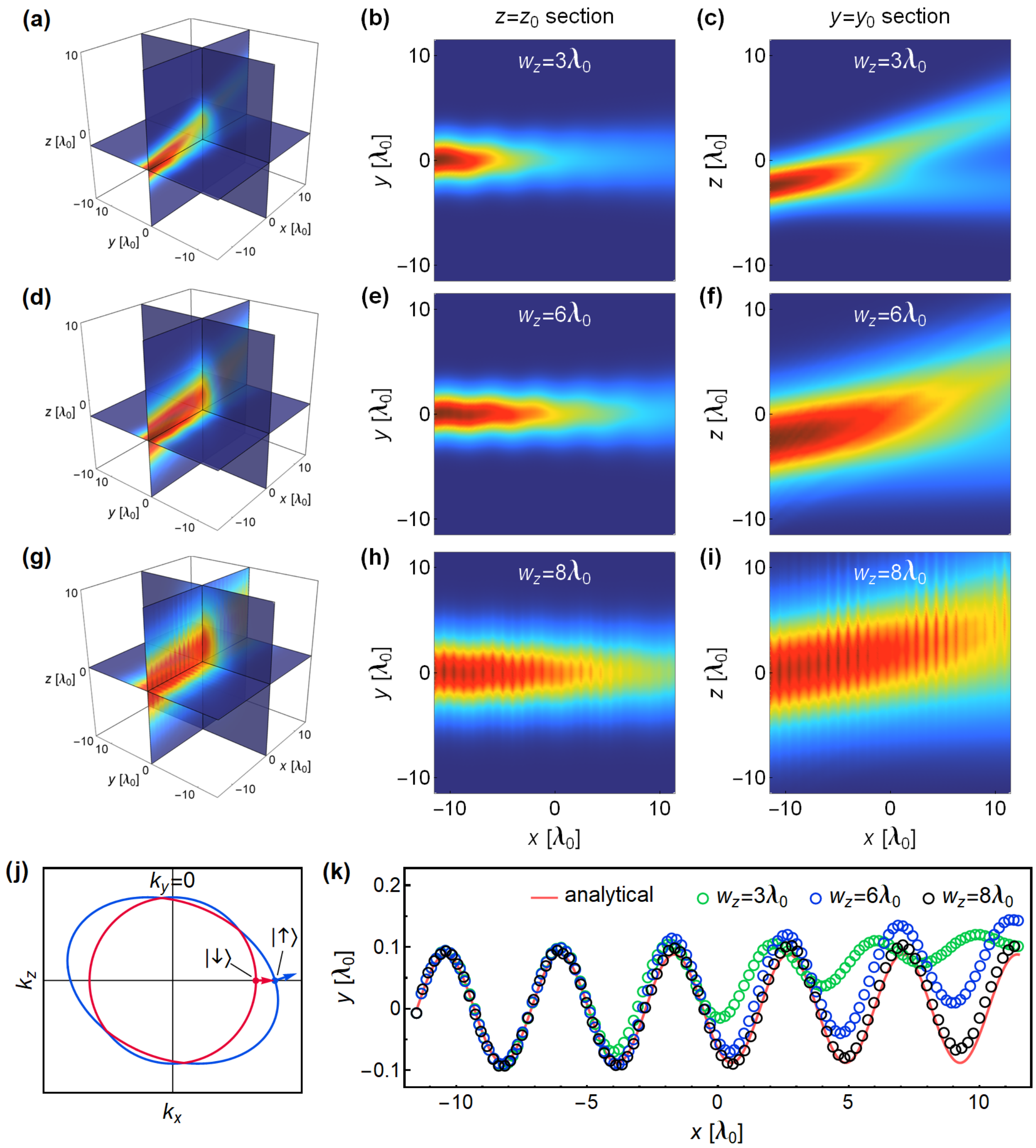

Supplementary Figure 4. ZB effect of 3D beams in a biaxial dielectric medium with the parameters $\overleftrightarrow{\varepsilon}_T = \overleftrightarrow{I}_{2\times 2}$, $\varepsilon_z = 1.6$, $\mathbf{g}_1 = (0.3, 0)^\top$, and $\mu/\mu_0 = 1$. The beam waists in the z directions are respectively (a-c) $w_z = 3\lambda_0$, (d-f) $w_z = 6\lambda_0$, (g-i) $w_z = 8\lambda_0$. (a,d,g) Slice view, (b,e,h) $z = z_0$ section view, and (c,f,i) $y = y_0$ section view of full-wave simulated intensity distributions of the 3D beams, where (y_0, z_0) denotes the center of beam on the initial plane $x = x_0$. (j) Isofrequency contours of the medium in the xz-plane, where the red and blue arrows denote the group velocity directions of the two eigenmodes with $\mathbf{k}$ along the positive k_x-axis. (k) Centroid trajectories of the 2D theory (red curve) and of the numerical fields on $z = z_0$ section (black circles) for the three simulated 3D beams shown in (b,e,h).

Supplementary Note 5. Theory for genuine non-Abelian Aharonov-Bohm system

In this section, we supply a self-content introduction of genuine non-Abelian AB system in a more rigorous manner. We will give its precise definition, prove the core property of AB effect, namely the AB phase factors for two homotopic closed paths are identical, and finally deduce the necessary conditions for constructing a genuine non-Abelian AB system.

1. Definition of Genuine non-Abelian AB system

Consider a simply connected space M endowed with a gauge structure. Mathematically, this system is described by a G-principal fiber bundle, $E \to M$, where E denotes the total bundle space, M denotes the base manifold, and G denotes the gauge group (the fiber $f_{\mathbf{x}}$ is homeomorphic to G at each $\mathbf{x} \in M$). If the gauge field (G-curvature) is zero $\hat{\mathcal{F}}_{\mu\nu} = 0$ (i.e. G- connection is flat) in the whole space, the gauge potential can be globally written as a pure gauge

$$\hat{\mathcal{A}}_\mu = i\,\hat{U}\partial_\mu \hat{U}^{-1}, \qquad (\,\hat{U} \in G\,) \tag{31}$$

and can be gauged away via global gauge transformation $\hat{U}^{-1}$:

$$\hat{\mathcal{A}}'_\mu = \hat{U}^{-1}\hat{\mathcal{A}}_\mu \hat{U} + i\,\hat{U}^{-1}\partial_\mu \hat{U} = 0. \tag{32}$$

Therefore, the pure gauge should have no measurable effect in a simply connected space. However, if the region of zero curvature $\hat{\mathcal{F}}_{\mu\nu} = 0$ is only a multiply connected subspace of the whole spacetime, the situation turns to be very interesting. In this case, although the gauge potential still can be written as $\hat{\mathcal{A}}_\mu = i\,\hat{U}\partial_\mu \hat{U}^{-1}$ locally, it cannot be gauged away globally via gauge transformation in the whole multiply connected space, and therefore implies nontrivial physical effect. Such a field-free system with irremovable gauge potential is referred to as an **Aharonov-Bohm system** [12].

Consider a particle (wave packet) characterized by a spinor state $|\psi\rangle$ propagating in the zero-field region. From a semi-classical picture, the particle will trace the same trajectory as the case of $\hat{\mathcal{A}} = 0$ due to the vanishing non-Abelian Lorentz force. Nevertheless, the evolution of the state vector $|\psi\rangle$ along a curve $\gamma : [0,1] \to M$ is affected by the gauge potential as follows:

$$|\psi\rangle = \mathcal{P}\,\exp\left[i\int_\gamma \hat{\mathcal{A}}_\mu dx^\mu\right]|\psi\rangle_0 = \hat{U}_\gamma\;|\psi\rangle_0\,. \tag{33}$$

Here, $|\psi\rangle_0$ denotes the state vector along the same path when $\hat{\mathcal{A}} = 0$, where the dynamic phase is included in it. As shown in Eq. (33), the nonzero gauge potential $\hat{\mathcal{A}}$ generates an addition geometric phase factor, namely the non-Abelian AB phase factor, expressed by a path-ordering integration along the curve γ stating at point $\gamma(0) = \mathbf{x}_0$:

$$\hat{U}_\gamma = \mathcal{P}\,\exp\left[i\int_\gamma \hat{\mathcal{A}}_\mu dx^\mu\right] \in G. \tag{34}$$

From a geometric point of view, this AB phase factor corresponds to the parallel transport $\mathrm{T}_\gamma(p_0) = p_0 \cdot \hat{U}_\gamma = \tilde{\gamma}(1)$ along the horizontally lifted path $\tilde{\gamma} : [0,1] \to E$ in the bundle space E, where $p_0 = \tilde{\gamma}(0) \in f_{\mathbf{x}_0}$ denotes the starting point of the lifted curve, and $f_{\mathbf{x}_0}$ denotes the fiber at $\mathbf{x}_0$. For a certain reference point p_0, the horizontal lift $\tilde{\gamma}$ of γ is unique. Physically, the choice of p_0 determines the local gauge $\hat{\mathcal{A}}(\mathbf{x}_0)$ at $\mathbf{x}_0$. And according to the property of parallel transport, the non-Abelian AB phase factor of a concatenate path $\gamma_1 \circ \gamma_2$ such that $\gamma_1(0) = \gamma_2(1)$ satisfies $\hat{U}_{\gamma_1\circ\gamma_2} = \hat{U}_{\gamma_1} \circ \hat{U}_{\gamma_2}$.

For a closed path (loop) c starting and ending at the same basepoint $\mathbf{x}_0$, a local gauge fixes the starting point $p_0 = \tilde{c}(0) \in f_{\mathbf{x}_0}$ of the lifted path $\tilde{c}$. However, $\tilde{c}$ need not to be closed, i.e. the end point of $\tilde{c}$ can be different from p_0, as shown in Supplementary Figure 5(c). The non-Abelian phase factor for the closed loop c,

$$\hat{\mathcal{U}}_c(\hat{\mathcal{A}}) = \mathcal{P}\,\exp\left[i\oint_c \hat{\mathcal{A}}_\mu dx^\mu\right], \tag{35}$$

is called the **holonomy** of the horizontally lifted loop $\tilde{c}$ with respect to the gauge $\hat{\mathcal{A}}$. The holonomies corresponding to all those closed loops based at $\mathbf{x}_0$ constitute a subgroup of the gauge group G:

$$\mathrm{Hol}_{p_0}(\hat{\mathcal{A}}) = \left\{\hat{\mathcal{U}}_c(\hat{\mathcal{A}})\,\middle|\; c(0) = c(1) = \mathbf{x}_0,\;\tilde{c}(0) = p_0\right\} \subseteq G, \tag{36}$$

which is the **holonomy group** of $\hat{\mathcal{A}}$ with the reference point p_0. $\hat{\mathcal{U}}_c(\hat{\mathcal{A}}) = \mathbb{I}$ if and only if $\tilde{c}$ is also closed, corresponding to the unit element of $\mathrm{Hol}_{p_0}(\hat{\mathcal{A}})$ [13]. In flat bundles with zero curvature $\hat{\mathcal{F}}_{\mu\nu} = 0$, the holonomy group of the flat connection $\hat{\mathcal{A}}$ is primarily determined by the topology of the base manifold M. The illustration of the holonomies in a flat bundle in shown in Supplementary Figure 5(c).

The Aharonov-Bohm system with non-Abelian gauge potential was first introduced by Wu and Yang in their paper connecting gauge theory with fiber bundle [14], where they predicted intriguing phenomena for the scattering of nucleon carrying weak isospin by a SU(2) flux tube, such as the spatial fluctuation of proton-neutron mixing ratio. Later on, Aharonov and Casher proposed an electric counterpart of the original AB effect, namely the scattering of neutral magnetic dipoles by a charged line [15]. Indeed, both the Wu-Yang scheme and the Aharonov-Casher effect are mathematically equivalent to an AB system with a SU(2) non-Abelian vortex [16, 17]. And the optical realization with anisotropic media has been proposed in Ref. 1. However, it can be shown that this kind of systems with a single SU(2) vortex always can be decomposed into two decoupled Abelian subsystems under a proper gauge [18], and the holonomy for a closed path c_n winding around the vortex n times takes the form [13]

$$\hat{\mathcal{U}}_{c_n} = \mathcal{P}\exp\left[\oint_{c_n} \hat{\mathcal{A}}_\mu dx^\mu\right] = \exp\left(i\,n\Phi\,\hat{\sigma}_3\right) = \begin{pmatrix} \exp(i\,n\Phi) & 0 \\ 0 & \exp(-i\,n\Phi) \end{pmatrix}, \tag{37}$$

where $\Phi\hat{\sigma}_3$ is the non-Abelian flux of the vortex. Accordingly, the holonomices of arbitrary two loops commute with each other, thus the holonomy group is still Abelian. In this sense, such systems can be still treated as Abelian or apparently non-Abelian, as specified in the main text [19, 20]. In a more rigorous manner, a genuine non-Abelian AB system can be defined as follows [19]:

Definition 1. *An Aharonov-Bohm system is called* ***genuinely non-Abelian*** *if and only if the corresponding Holonomy group* $\mathrm{Hol}(\hat{\mathcal{A}})$ *of the flat connection (gauge potential)* $\hat{\mathcal{A}}$ *is a non-Abelian group.*

It is noteworthy that a different route of generalizing AB effect is the non-Abelian vortex-vortex scattering [21–24]. In contrast to the non-Abelian vortices in this route being dynamic quasi-particles and respecting non-Abelian statistics, the non-Abelian vortices in our AB systems are merely non-dynamic background.

2. Necessary conditions of genuine non-Abelian AB system

In order to characterize the holonomy group of a flat bundle, we first introduce the concept of path homotopy. **Path homotopy** refers to a topological equivalence relation "$\simeq$" for paths sharing the fixed endpoints. Intuitively, if a path γ_1 can deform continuously into another path γ_2 while keeping the endpoints fixed, γ_1 and γ_2 are said to be homotopic to each other, $\gamma_1 \simeq \gamma_2$ (see Supplementary Figure 5(a)). Strictly speaking, path homotopy denotes precisely the mapping of the continuous deformation from γ_1 to γ_2 [25]. All the paths that are homotopic to each other form the **homotopy equivalence class** $[\gamma] = \left\{\gamma' \middle| \gamma'(0) = \mathbf{x}_0, \gamma'(1) = \mathbf{x}_1, \gamma' \simeq \gamma\right\}$, where any element γ of the class can be chosen as the class representative.

For closed paths through the same basepoint $\mathbf{x}_0$ in the base manifold, their homotopy classes constitute the quotient set with respect to the homotopy equivalence relation "$\simeq$":

$$\pi_1(M, \mathbf{x}_0) = \left\{[c] \,\middle|\, c(0) = c(1) = \mathbf{x}_0\right\} = \left\{c \,\middle|\, c(0) = c(1) = \mathbf{x}_0\right\}\Big/ \simeq . \tag{38}$$

By introducing the multiplication between classes, $[c_1] \circ [c_2] = [c_1 \circ c_2]$ ($c_1 \circ c_2$ denotes the concatenation of the two loops c_1, c_2), $\pi_1(M, \mathbf{x}_0)$ also forms a group, known as the **first fundamental group** of the base manifold M. Though the definition of fundamental group relies on the basepoint $\mathbf{x}_0$, it can be proved that $\pi_1(M, \mathbf{x}_0)$ and $\pi_1(M, \mathbf{x}_0')$ are isomorphic for arbitrary $\mathbf{x}_0, \mathbf{x}_0' \in M$ as long as M is path-connected. The fundamental group is an important topological invariant for characterizing the properties of a manifold. Moreover, we will prove that the first fundamental group of the base manifold is homomorphic to the holonomy group of the corresponding flat bundle in the following part of this section.

In AB systems with Abelian gauge potentials, it is well-known that the AB phase factors along two homotopic loops c_1, c_2 are identical due to the null magnetic flux passing through the enclosed area of the combined loop $c_1 - c_2$. Indeed, this result can be generalized to a theorem for generic AB systems with non-Abelian gauge potentials as follows.

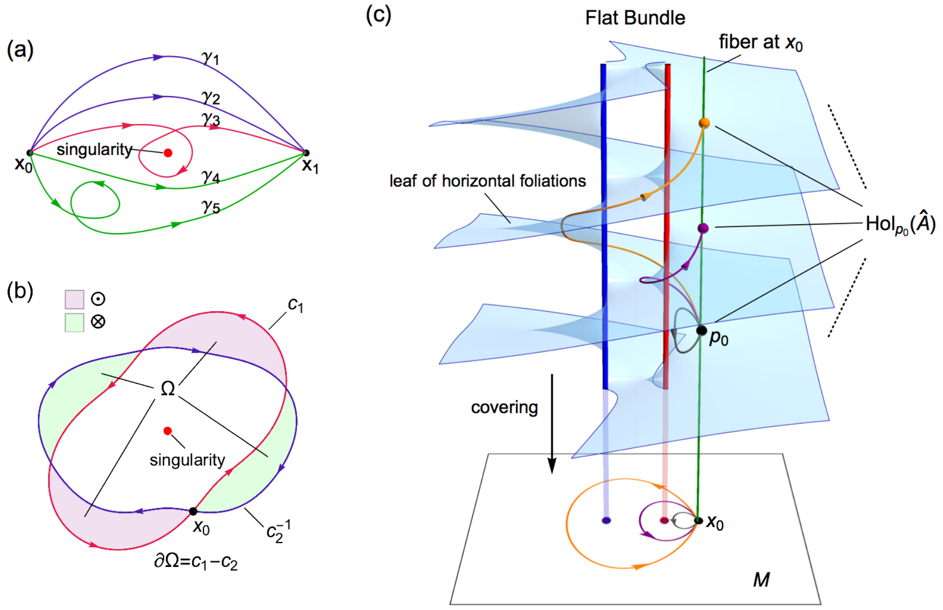

Supplementary Figure 5. (a) Illustration of homotopic paths with fixed endpoints $\mathbf{x}_0$, $\mathbf{x}_1$ in a punctured plane. The curves in a same color can deform continuously into each other and thus are path-homotopic, namely $\gamma_1 \simeq \gamma_2$, $\gamma_4 \simeq \gamma_5$, whereas the curves in different colors are not homotopic. (b) Two homotopic loops based at $\mathbf{x}_0$ form the boundary of a 2D orientable region Ω (mathematically, a 2-chain). The purple (green) color indicates the orientation of the region is outward (inward) the paper plane. (c) Holonomies in a flat bundle with a twice-punctured base manifold M. For the three closed paths based at $\mathbf{x}_0$ in different homotopy classes, their horizontally lifted paths in the bundle space start from p_0 to different terminals on the fiber $f_{\mathbf{x}_0}$. All of the terminals of such lifted paths compose the holonomy group $\mathrm{Hol}_{p_0}(\hat{\mathcal{A}})$. The light blue surface illustrates a leaf of horizontal foliations through the point p_0 embedding in the bundle space, which covers the base manifold M. Note: the surface is merely a cartoon schematic but not an actual universal covering space of M. Thus the lifted paths on this surface cannot differentiate all of different homotopy classes.

Theorem 1. *In a flat bundle, the holonomies of two path-homotopic loops c_1 and c_2, i.e. they belong to the same homotopy class $c_1, c_2 \in [c]$, are identical*

$$\hat{\mathcal{U}}_{c_1}(\hat{\mathcal{A}}) = \hat{\mathcal{U}}_{c_2}(\hat{\mathcal{A}}) \equiv \hat{\mathcal{U}}_{[c]}(\hat{\mathcal{A}}). \tag{39}$$

Proof. The proof of this theorem can be found in the textbooks of differential geometry [26] and in the literature [19, 27]. For completeness, we give a concise but accessible proof imitating the derivation of the Abelian case. Let us examine the following product

$$\hat{\mathcal{U}}_{c_1}(\hat{\mathcal{A}})\hat{\mathcal{U}}_{c_2}(\hat{\mathcal{A}})^{-1} = \hat{\mathcal{U}}_{c_1}(\hat{\mathcal{A}})\hat{\mathcal{U}}_{c_2^{-1}}(\hat{\mathcal{A}}) = \hat{\mathcal{U}}_{c_1\circ c_2^{-1}}(\hat{\mathcal{A}}) = \mathcal{P}\exp\left[i\oint_{c_1\circ c_2^{-1}}\hat{\mathcal{A}}_\mu dx^\mu\right].$$

The Hurewicz's theorem implies that the two homotopic loops c_1, c_2 are necessarily homologous when treated as 1-cycles [25]. According to the definition of homology, $c_1 \circ c_2^{-1} \sim c_1 - c_2 = \partial\Omega$ must be the boundary of a 2-chainΩ (the 2D orientable region enclosed by $c_1 - c_2$ as shown in Supplementary Figure 5(b)). In terms of the **non-Abelian Stokes theorem** [28–30] and $\hat{\mathcal{F}}_{\mu\nu} = 0$, the path-ordered exponential along the loop $c_1 \circ c_2^{-1}$ equals the identity element of G:

$$\hat{\mathcal{U}}_{c_1}(\hat{\mathcal{A}})\hat{\mathcal{U}}_{c_2}(\hat{\mathcal{A}})^{-1} = \mathcal{P}\exp\left[i\oint_{\partial\Omega}\hat{\mathcal{A}}_\mu dx^\mu\right] = \mathscr{P}_s\exp\left[\frac{i}{2}\int_\Omega \hat{U}(\mathbf{x},\mathbf{x}_0)^{-1}\underbrace{\hat{\mathcal{F}}_{\mu\nu}(\mathbf{x})}_{=0}\hat{U}(\mathbf{x}_0,\mathbf{x})\,dx^\mu\wedge dx^\nu\right] = \mathbb{I}, \tag{40}$$

where $\mathscr{P}_s$ denotes the "surface ordering", and $\hat{U}(\mathbf{x}_0,\mathbf{x}) = \mathcal{P}\exp\left[i\int_{\mathbf{x}}^{\mathbf{x}_0}\hat{\mathcal{A}}_\mu dx^\mu\right]$ is the non-Abelian phase factor along a

path connecting the field point $\mathbf{x}$ and the basepoint $\mathbf{x}_0$. As such, the homotopic invariance of the holonomies in flat bundle has been proved. □

Physically, the theorem manifests the quantization of the AB phase factors along paths with fixed endpoints, namely they can only take discrete values (in general, they are matrices) for different homotopic paths, while the AB phase factor is invariant against the continuous deformation of the path in the field-free region. This is the key signature of AB systems associated with either Abelian or non-Abelian gauge potentials. In a simply connected space, the fundamental group is trivial, namely all loops belong to $[\mathbf{x}_0]$. Hence, any state will return to itself as it travels along a closed loop. **Therefore, an AB system requires the fundamental group of the base manifold is nontrivial.** According to **Theorem 1**, the holonomy group of a flat bundle and the fundamental group of the corresponding base manifold are naturally connected.

Corollary 1. *For a flat bundle $E \to M$, there is a* ***surjective group homomorphism*** *from the first fundamental group $\pi_1(M, \mathbf{x}_0)$ of the base manifold M to the holonomy group $\mathrm{Hol}_{p_0}(\hat{\mathcal{A}})$ of the bundle:*

$$\pi_1(M, \mathbf{x}_0) \ni [c] \longrightarrow \hat{\mathcal{U}}_{[c]}(\hat{\mathcal{A}}) \in \mathrm{Hol}_{p_0}(\hat{\mathcal{A}}) = \left\{ \hat{\mathcal{U}}_{[c]} \,\middle|\, [c] \in \pi_1(M, \mathbf{x}_0),\ \tilde{c}(0) = p_0 \right\}. \tag{41}$$

In other words, the holomony group $\mathrm{Hol}_{p_0}(\hat{\mathcal{A}})$ of the flat bundle is just a representation of the fundamental group $\pi_1(M, \mathbf{x}_0)$ of the base manifold.

As the holonomy group $\mathrm{Hol}(\hat{\mathcal{A}})$ is a subgroup of the gauge group G, an Abelian gauge group indicates all of the holonomies commute with each other. Meanwhile, in terms of **Corollary 1**, for an AB system with a non-Abelian holonomy group, there should exit two loops c_1, c_2 such that $\hat{\mathcal{U}}_{[c_1]}\hat{\mathcal{U}}_{[c_2]} \neq \hat{\mathcal{U}}_{[c_2]}\hat{\mathcal{U}}_{[c_1]}$. Thus $[c_1 \circ c_2] \neq [c_2 \circ c_1]$, in other words, $\pi_1(M)$ must also be non-Abelian. To summarize, we obtain

Necessary conditions for genuine non-Abelian AB systems:

1. *The gauge group G is non-Abelian;*
2. *The first fundamental group $\pi_1(M)$ of the base manifold is non-Abelian.*

Supplementary Note 6. Material construction of non-Abelian AB system

Inspired by the scheme of two noncommutative $SU(3)$ vortices proposed in Ref. 19, we will proceed to construct a SU(2) genuine non-Abelian AB system. We assume the synthetic SU(2) gauge potential only carrying two nonzero components: $\hat{\mathcal{A}} = \mathcal{A}^1\hat{\sigma}_1 + \mathcal{A}^2\hat{\sigma}_2$. Besides, a nontrivial gauge potential with vanishing field $\hat{\mathcal{F}}_{ij} = 0$ can be locally expressed in the pure gauge form $\hat{\mathcal{A}} = i\hat{U}\nabla\hat{U}^{-1}$. If we demand $\mathcal{A}^2 = 0$ when $y > 0$, and $\mathcal{A}^1 = 0$ when $y < 0$, the unitary transformation matrix can be simply expressed as follows in upper and lower semi-space respectively:

$$\hat{U} = \exp\left(i\,\zeta_1(\mathbf{r})\,\hat{\sigma}_1\right) \quad (y>0), \qquad \hat{U} = \exp\left(i\,\zeta_2(\mathbf{r})\,\hat{\sigma}_2\right) \quad (y<0). \tag{42}$$

Here, $\zeta_1(\mathbf{r})$, $\zeta_2(\mathbf{r})$, referred to as pre-potentials, are some multivalued functions of x, y that smoothly tend to zero at $y = 0$, and the corresponding components of the vector potential take the form $\mathcal{A}^i = \nabla\zeta_i(\mathbf{r}) \quad (i = 1, 2)$.

The pre-potentials with nontrivial topology can be constructed in the following way:

$$\zeta_1(\mathbf{r}) = \frac{\Phi_1}{2\pi}S(\mathbf{r})\,\phi(\mathbf{r}-\mathbf{r}_0), \quad \zeta_2(\mathbf{r}) = \frac{\Phi_2}{2\pi}S(-\mathbf{r})\,\phi(\mathbf{r}+\mathbf{r}_0), \tag{43}$$

with the corresponding synthetic gauge potential

$$\hat{\mathcal{A}} = \begin{cases} \mathcal{A}^1\hat{\sigma}_1 = \dfrac{\Phi_1}{2\pi}\nabla\Big(S(\mathbf{r})\,\phi(\mathbf{r}-\mathbf{r}_0)\Big)\hat{\sigma}_1 = \dfrac{\Phi_1}{2\pi}\Big(S(\mathbf{r})\;\nabla\phi(\mathbf{r}-\mathbf{r}_0) + \phi(\mathbf{r}-\mathbf{r}_0)\nabla S(\mathbf{r})\Big)\hat{\sigma}_1, & (y>0) \\ \mathcal{A}^2\hat{\sigma}_2 = \dfrac{\Phi_2}{2\pi}\nabla\Big(S(-\mathbf{r})\,\phi(\mathbf{r}+\mathbf{r}_0)\Big)\hat{\sigma}_2 = \dfrac{\Phi_2}{2\pi}\Big(S(-\mathbf{r})\underbrace{\nabla\phi(\mathbf{r}+\mathbf{r}_0)}_{\text{vortex}} + \phi(\mathbf{r}+\mathbf{r}_0)\nabla S(-\mathbf{r})\Big)\hat{\sigma}_2, & (y<0) \end{cases} \tag{44}$$

where $\phi(\mathbf{r}\mp\mathbf{r}_0)$ is the polar angle (see Supplementary Figure 6(a)) with respect to $\pm\mathbf{r}_0 = \pm r_0\,\mathbf{e}_y$ whose gradient represents an irrotational free vortex located at $\pm\mathbf{r}_0$:

$$\nabla\phi(\mathbf{r}\mp\mathbf{r}_0) = \frac{\mathbf{e}_\phi}{|\mathbf{r}\mp\mathbf{r}_0|} = \frac{-(y\mp r_0)\,\mathbf{e}_x + x\,\mathbf{e}_y}{|\mathbf{r}\mp\mathbf{r}_0|^2}, \tag{45}$$

and $S(\pm\mathbf{r})$ is a smooth cutoff function such that (i) the two components $\mathcal{A}^1, \mathcal{A}^2$ are gradually compressed in the upper and lower half-space without overlap, and (ii) $\mathcal{A}^{1,2}$ tends to a free vortex nearby $\pm\mathbf{r}_0$ respectively, i.e.

$$\begin{aligned} &\text{(i)} \quad \mathcal{A}^i \to 0 && \Leftrightarrow \quad S(\pm\mathbf{r}) \to 0, \quad \text{as } y \to 0; \\ &\text{(ii)} \quad \mathcal{A}^i = \frac{\Phi_i}{2\pi}\nabla\phi(\mathbf{r}\mp\mathbf{r}_0) && \Leftrightarrow \quad S(\pm\mathbf{r}) = 1, \quad \text{as } |\mathbf{r}\mp\mathbf{r}_0| < \delta r. \end{aligned}$$

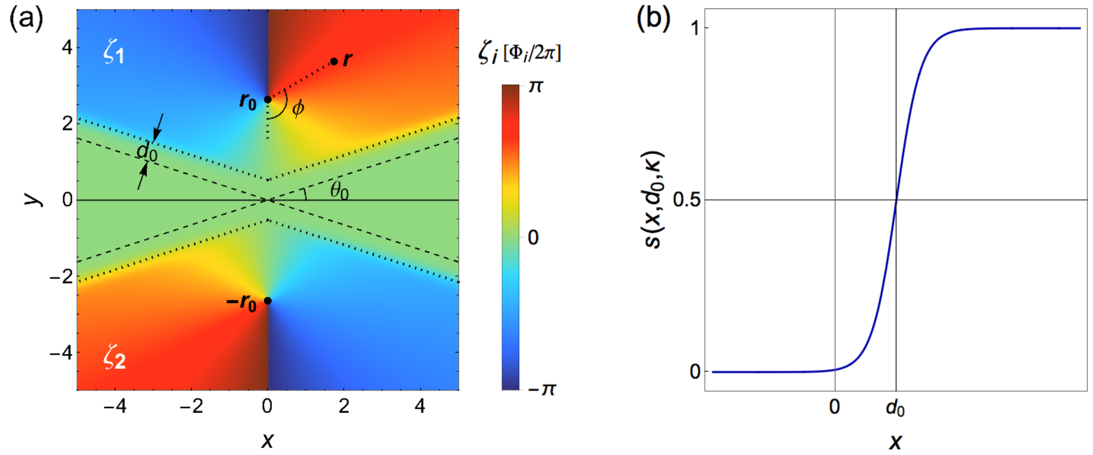

Supplementary Figure 6. (a) Spatial distributions of the pre-potentials ζ_1 (in the upper half-plane $y > 0$) and ζ_2 (in the lower half-plane $y < 0$), whose gradients give the corresponding components of the synthetic SU(2) gauge potential $\mathcal{A}^i = \nabla\zeta_i$ $(i = 1, 2)$. (b) The profile of the sigmoid function $s(x, d_0, \kappa)$ for constructing the cutoff function $S(\mathbf{r})$ in Eq. (47).

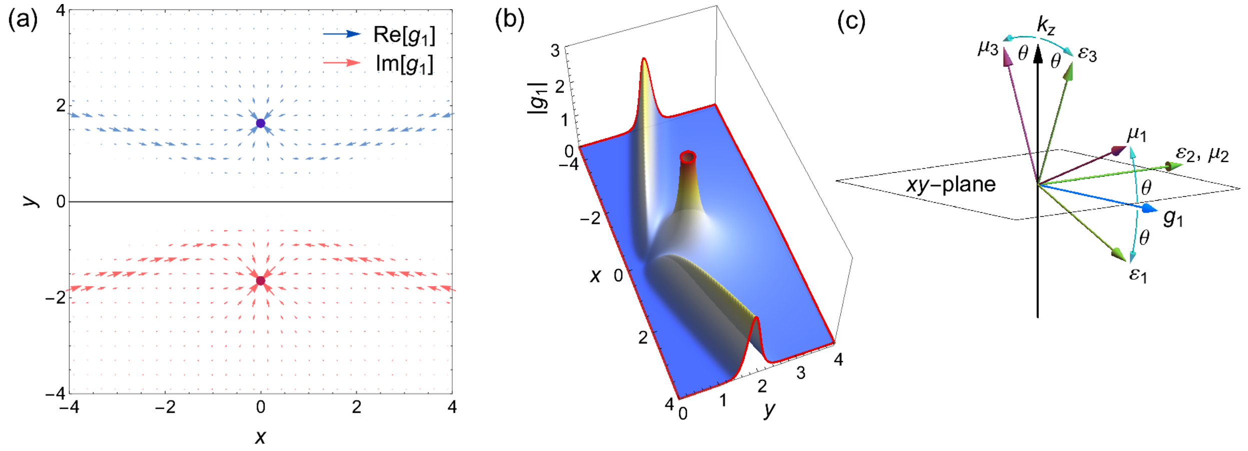

Supplementary Figure 7. (a) Spatial distribution of $\mathbf{g}_1 = -\mathbf{g}_2^*$ (the off-diagonal term of $\overleftrightarrow{\varepsilon}$, $\overleftrightarrow{\mu}$). (b) Distribution of $|\mathbf{g}_1|$, which determines the principal components of $\overleftrightarrow{\varepsilon}$, $\overleftrightarrow{\mu}$ (see Eq. (51)), for the reciprocal anisotropic medium in the upper half-plane. (c) The orientation of the principal frames of $\overleftrightarrow{\varepsilon}$, $\overleftrightarrow{\mu}$ for the medium in the upper half-plane.

Meanwhile, since $\phi(\mathbf{r}\mp\mathbf{r}_0)$ is multivalued, the branch cut of $\phi(\mathbf{r}\mp\mathbf{r}_0)$ is selected along $\mathbf{r} = \pm(\mathbf{r}_0 + y\mathbf{e}_y)$ $(y > 0)$ as shown in Supplementary Figure 6(a). To ensure the monodromy of $\mathcal{A}^i$, we should also require $S(\pm\mathbf{r}) = 1$ in a neighborhood of the branch cut of $\phi(\mathbf{r}\mp\mathbf{r}_0)$. The form of $S(\mathbf{r})$ is not unique, here we adopt an approximate but rather simple expression:

$$S(\mathbf{r}) = s(y\cos\theta_0 + x\sin\theta_0, \kappa, d_0)\cdot s(y\cos\theta_0 - x\sin\theta_0, \kappa, d_0) \tag{46}$$

with the sigmoid function

$$s(x, k, d_0) = \frac{1}{\exp[\kappa(d_0 - x)] + 1}, \tag{47}$$

and the values of the parameters θ_0, κ, d_0 do not affect the AB phase factors as long as the asymptotic conditions of $S(\pm\mathbf{r})$ are met. The obtained pre-potentials ζ_1, ζ_2 and the gauge potential $\hat{\mathcal{A}}$ are plotted in Supplementary Figure 7(a) and in Fig. 2a of the main text respectively.

Notably, the non-Abelian holonomy of a loop encircling a vortex depends on the choice of basepoint. Nevertheless, in our system, the gauge potential is commutative in either upper or lower half-space. For an arbitary loop c_1 (c_2) lying entirely in the upper (lower) half-space and encircling the upper (lower) vortex anticlockwise once, its holonomy, $\hat{\mathcal{U}}_{[c_i]}$ $(i = 1, 2)$, is uniquely determined as

$$\hat{\mathcal{U}}_{c_i} = \mathcal{P}\exp\left[i\oint_{c_i}\hat{\mathcal{A}}^i\cdot d\mathbf{r}\,\hat{\sigma}_i\right] = \exp\left[i\oint_{c_i}d\zeta_i\,\hat{\sigma}_i\right] = \exp\left[i\frac{\Phi_i}{2\pi}\oint_{|\mathbf{r}\mp\mathbf{r}_0|<\delta r}d\phi\,\hat{\sigma}_i\right] = \exp\left[i\Phi_i\hat{\sigma}_i\right], \quad (i = 1, 2) \tag{48}$$

where the integral in the second-to-last step is along a sufficiently small circle enclosing the vortex, and Φ_1, Φ_2 can be regarded as the flux of the two vortices respectively. For loops through a basepoint on the x-axis $(y = 0)$, Eq. (48) is still valid. Therefore, $\hat{\mathcal{U}}_{c_1}, \hat{\mathcal{U}}_{c_2}$ can be used as the two generators to obtain the holonomy group (being homomorphic to the free group $\mathbb{Z}*\mathbb{Z}$) of this AB system. As shown in Supplementary Figure 6(a) as well as Fig. 2a of the main text, the cutoff function $S(\mathbf{r})$ in Eq. (46) renders the gauge potential $\hat{\mathcal{A}}$ vanishing in the whole conic region of $|\arctan(y/x)| < \theta_0$. As such, each point on the cross section of an optical beam with a finite width can undergo a unique non-Abelian AB phase factor when arriving at the screen.

From the synthetic gauge potential in Eq. (44) and Table. 1 in the main text, we can inversely construct the desired material parameters. To guarantee $\hat{\mathcal{A}}_0 \equiv 0$ and $V_0 = \text{const.}$, we demand that the diagonal components of $\overleftrightarrow{\varepsilon}$, $\overleftrightarrow{\mu}$ obey $\varepsilon_T = \mu_T$, $\varepsilon_z = \mu_z$ and are homogeneous in the whole space, meanwhile, their off-diagonal parts satisfy $\mathbf{g}_1 = -\mathbf{g}_2^*$. As such, the synthetic $\hat{\sigma}_1$-vortex in the upper half-space $(y > 0)$ can be made using reciprocal anisotropic metamaterials [1] with $\mathbf{g}_1 = -\mathbf{g}_2 = \mathbf{e}_z\times\mathcal{A}^1/k_0$. The constitutive tensors take the following forms:

$$\overleftrightarrow{\varepsilon}/\varepsilon_0 = \left(\begin{array}{cc|c} \varepsilon_T & 0 & -\mathcal{A}_y^1/k_0 \\ 0 & \varepsilon_T & \mathcal{A}_x^1/k_0 \\ \hline -\mathcal{A}_y^1/k_0 & \mathcal{A}_x^1/k_0 & \varepsilon_z \end{array}\right), \qquad \overleftrightarrow{\mu}/\mu_0 = \left(\begin{array}{cc|c} \varepsilon_T & 0 & \mathcal{A}_y^1/k_0 \\ 0 & \varepsilon_T & -\mathcal{A}_x^1/k_0 \\ \hline \mathcal{A}_y^1/k_0 & -\mathcal{A}_x^1/k_0 & \varepsilon_z \end{array}\right). \tag{49}$$

Whereas the synthetic $\hat{\sigma}_2$-vortex in the lower half-space ($y < 0$) need to be constructed out of gyrotropic materials with $\mathbf{g}_1 = \mathbf{g}_2 = i\,\mathbf{e}_z \times \mathcal{A}^2/k_0$, so the relative permittivity and permeability are of entirely equal form

$$\overleftrightarrow{\varepsilon}/\varepsilon_0 = \overleftrightarrow{\mu}/\mu_0 = \left(\begin{array}{cc|c} \varepsilon_T & 0 & -i\mathcal{A}_y^2/k_0 \\ 0 & \varepsilon_T & i\mathcal{A}_x^2/k_0 \\ \hline i\mathcal{A}_y^2/k_0 & -i\mathcal{A}_x^2/k_0 & \varepsilon_z \end{array}\right). \tag{50}$$

The orientation of the off-diagonal vector $\mathbf{g}_1 = -\mathbf{g}_2^*$ is plotted in Supplementary Figure 7(a).

We further analyze the property of the reciprocal anisotropic medium used in the upper half-plane. By diagonalizing $\overleftrightarrow{\varepsilon}$ and $\overleftrightarrow{\mu}$ given by Eq. (49) into $\tilde{\varepsilon}/\varepsilon_0 = \mathrm{diag}(\varepsilon_1, \varepsilon_2, \varepsilon_3)$ and $\tilde{\mu}/\mu_0 = \mathrm{diag}(\mu_1, \mu_2, \mu_3)$, we obtain their principal components:

$$\begin{aligned} \varepsilon_1 = \mu_1 &= \frac{1}{2}\left(\varepsilon_T + \varepsilon_z - \sqrt{(\varepsilon_T - \varepsilon_z)^2 + 4|\mathbf{g}_1|^2}\right), \\ \varepsilon_2 = \mu_2 &= \varepsilon_T, \\ \varepsilon_3 = \mu_3 &= \frac{1}{2}\left(\varepsilon_T + \varepsilon_z + \sqrt{(\varepsilon_T - \varepsilon_z)^2 + 4|\mathbf{g}_1|^2}\right). \end{aligned} \tag{51}$$

The distribution of $|\mathbf{g}_1|$ is plotted in Supplementary Figure 7(b). Despite the identical principal components of $\overleftrightarrow{\varepsilon}$ and $\overleftrightarrow{\mu}$, their principal frames do not coincide as shown in Supplementary Figure 7(c). For a certain $\mathbf{g}_1 = -\mathbf{g}_2$, we have

$$\begin{aligned} &\mathbf{e}_{\varepsilon_1} = \cos\theta\,\mathbf{e}_{\mathbf{g}_1} - \sin\theta\,\mathbf{e}_z, \quad \mathbf{e}_{\varepsilon_2} = \mathbf{e}_z \times \mathbf{e}_{\mathbf{g}_1}, \quad \mathbf{e}_{\varepsilon_3} = \sin\theta\,\mathbf{e}_{\mathbf{g}_1} + \cos\theta\,\mathbf{e}_z, \\ &\mathbf{e}_{\mu_1} = \cos\theta\,\mathbf{e}_{\mathbf{g}_1} + \sin\theta\,\mathbf{e}_z, \quad \mathbf{e}_{\mu_2} = \mathbf{e}_z \times \mathbf{e}_{\mathbf{g}_1}, \quad \mathbf{e}_{\mu_3} = -\sin\theta\,\mathbf{e}_{\mathbf{g}_1} + \cos\theta\,\mathbf{e}_z, \end{aligned} \tag{52}$$

where $\theta = \arctan\left[\frac{\sqrt{(\varepsilon_T - \varepsilon_z)^2 + 4|\mathbf{g}_1|^2} + (\varepsilon_T - \varepsilon_z)}{2|\mathbf{g}_1|}\right]$ is the angle between ε_1-axis and xy-plane. In particular, if $\varepsilon_z = \varepsilon_T$, the parameters are reduced to $\varepsilon_1 = \mu_1 = \varepsilon_T - |\mathbf{g}_1|$, $\varepsilon_2 = \mu_2 = \varepsilon_T$, $\varepsilon_3 = \mu_3 = \varepsilon_T + |\mathbf{g}_1|$, and $\theta = \pi/4$.

Supplementary Note 7. Non-Abelian AB interference

1. Spin density interference

In the main text, we have shown that the genuine non-Abelian nature of the AB system is characterized by the nontrivial interference of the two beams $\gamma_{\rm I}$, $\gamma_{\rm II}$. In what follows, we will prove that the interfering spin density is always perpendicular to $\Delta\vec{s} = \vec{s}_{\rm I} - \vec{s}_{\rm II}$ on the screen. The final normalized spinor states of the two beams are given by $|s_i\rangle = \hat{U}_{\gamma_i}|s_0\rangle$ ($i = {\rm I},{\rm II}$), thus the orientation of the spin density satisfies

$$\begin{aligned}\vec{s}(y) &\propto \left(\langle s_{\rm I}|e^{-i\theta_{\rm I}(y)} + \langle s_{\rm II}|e^{-i\theta_{\rm II}(y)}\right)\vec{\hat{\sigma}}\left(e^{i\theta_{\rm I}(y)}|s_{\rm I}\rangle + e^{i\theta_{\rm II}(y)}|s_{\rm II}\rangle\right)\\ &\propto \vec{s}_{\rm I} + \vec{s}_{\rm II} + 2{\rm Re}\left(e^{i\Delta\theta(y)}\langle s_{\rm I}|\vec{\hat{\sigma}}|s_{\rm II}\rangle\right).\end{aligned} \tag{53}$$

It is obvious that $(\vec{s}_{\rm I} + \vec{s}_{\rm II})\cdot\Delta\vec{s} = 0$, so we only need to prove $\langle s_{\rm I}|\vec{\hat{\sigma}}|s_{\rm II}\rangle\cdot\Delta\vec{s} = 0$. Assuming $|s_{\rm I}\rangle = (a,b)^\intercal$, $|s_{\rm II}\rangle = (c,d)^\intercal$, we can obtain

$$\begin{aligned}\langle s_{\rm I}|\vec{\hat{\sigma}}|s_{\rm II}\rangle &= (a^*d + b^*c)\vec{e}_1 + i(-a^*d + b^*c)\vec{e}_2 + (a^*c - b^*d)\vec{e}_3,\\ \vec{s}_{\rm I} &= 2\,{\rm Re}(ab^*)\vec{e}_1 + 2i\,{\rm Im}(ab^*)\vec{e}_2 + (|a|^2 - |b|^2)\vec{e}_3,\\ \vec{s}_{\rm II} &= 2\,{\rm Re}(cd^*)\vec{e}_1 + 2i\,{\rm Im}(cd^*)\vec{e}_2 + (|c|^2 - |d|^2)\vec{e}_3.\end{aligned}$$

Then, we have

$$\langle s_{\rm I}|\vec{\hat{\sigma}}|s_{\rm II}\rangle\cdot(\vec{s}_{\rm I} - \vec{s}_{\rm II}) = (a^*b + b^*d)\left(|a|^2 + |b|^2 - |c|^2 - |d|^2\right) = 0.$$

Consequently, the interfering pseudo-spins $\vec{s}(y)$ lie on the great circle perpendicular to the axis $\Delta\vec{s} = \vec{s}_{\rm I} - \vec{s}_{\rm II}$ on the Bloch sphere. In addition, the SU(2) phase factor $\exp[i\Phi_i\sigma_i]$ of winding around a vortex corresponds to a $SO(3)$ rotation of the pseudo-spin about the $\vec{e}_i$-axis clockwise through an angle $2\Phi_i$, i.e. $R_i = \exp[-2\Phi_i\vec{e}_i\times\mathbb{I}]$. As a result, the final spins of the two beams can be expressed as

$$\vec{s}_{\rm I} = R_{\gamma_{\rm I}}\vec{s}_0 = R_{\gamma_0}R_2R_1^{-1}\,\vec{s}_0, \tag{54a}$$

$$\vec{s}_{\rm II} = R_{\gamma_{\rm II}}\vec{s}_0 = R_{\gamma_0}R_1^{-1}R_2\,\vec{s}_0. \tag{54b}$$

Therefore, the axis $\Delta\vec{s}$ perpendicular to the spin density on screen is determined by the commutator of the two rotation matrices:

$$\Delta\vec{s} = R_{\gamma_0}\left[R_2, R_1^{-1}\right]\vec{s}_0 = 4\sin\Phi_1\,\sin\Phi_2\cdot\Big(\vec{u}(\Phi_1,\Phi_2)\times\vec{s}_0 - 2\vec{u}(\Phi_1,\Phi_2)\cdot(\vec{s}_0\times\vec{e}_3)\vec{e}_3\Big), \tag{55}$$

where $R_{\gamma_0} = \mathbb{I}$ in our case, and $\vec{u}(\Phi_1,\Phi_2)$ is a vector defined in the spin space:

$$\vec{u} = \cos\Phi_1\sin\Phi_2\,\vec{e}_1 + \sin\Phi_1\cos\Phi_2\,\vec{e}_2 + \cos\Phi_1\cos\Phi_2\,\vec{e}_3, \tag{56}$$

whose norm $|\vec{u}|^2 = 1 - \sin^2\Phi_1\,\sin^2\Phi_2 \le 1$. If the initial spin satisfies $\vec{u}(\Phi_1,\Phi_2)\times\vec{s}_0 - 2\vec{u}(\Phi_1,\Phi_2)\cdot(\vec{s}_0\times\vec{e}_3)\vec{e}_3 = 0$, the spins of the two beams will finally evolve into the same direction $\vec{s}_{\rm I} = \vec{s}_{\rm II}$, and hence the orientation of the spin density will not fluctuate on the screen. Nevertheless, the nontrivial AB effect can still be detected from the phase shift $\delta\theta$ and the contracted amplitude $b < 1$ in intensity interference pattern in this situation.

As for the intensity interference part $|\psi|^2$, the phase shift $\delta\theta$ and the relative amplitude b of the interference pattern can be explicitly written as functions of the vortex fluxes Φ_1, Φ_2 and the initial spin $\vec{s}_0$:

$$\delta\theta(\Phi_1,\Phi_2,\vec{s}_0) = \arctan\left[\frac{2\sin\Phi_1\sin\Phi_2}{1 - 2\sin^2\Phi_1\sin^2\Phi_2}\vec{s}_0\cdot\vec{u}(\Phi_1,\Phi_2)\right], \tag{57}$$

$$b(\Phi_1,\Phi_2,\vec{s}_0) = \Big[\big(1 - 2\sin^2\Phi_1\sin^2\Phi_2\big)^2 + 4\sin^2\Phi_1\sin^2\Phi_2\big(\vec{s}_0\cdot\vec{u}(\Phi_1,\Phi_2)\big)^2\Big]^{1/2}. \tag{58}$$

They are, obviously, periodic with respect to Φ_1 and Φ_2. In general, the relative amplitude $b \le 1$ with equality if and only if $\vec{s}_0$ is parallel to $\vec{u}$. Therefore, a contracted amplitude ($b < 1$) is a signature of the genuine non-Abelian AB interference. At the same time, the Wilson loop of c_0 is given by

$$W(c_0) = {\rm Tr}\hat{\mathcal{U}}_{c_0} = 2b\cos\delta\theta = 2 - 4\sin^2\Phi_1\sin^2\Phi_2. \tag{59}$$

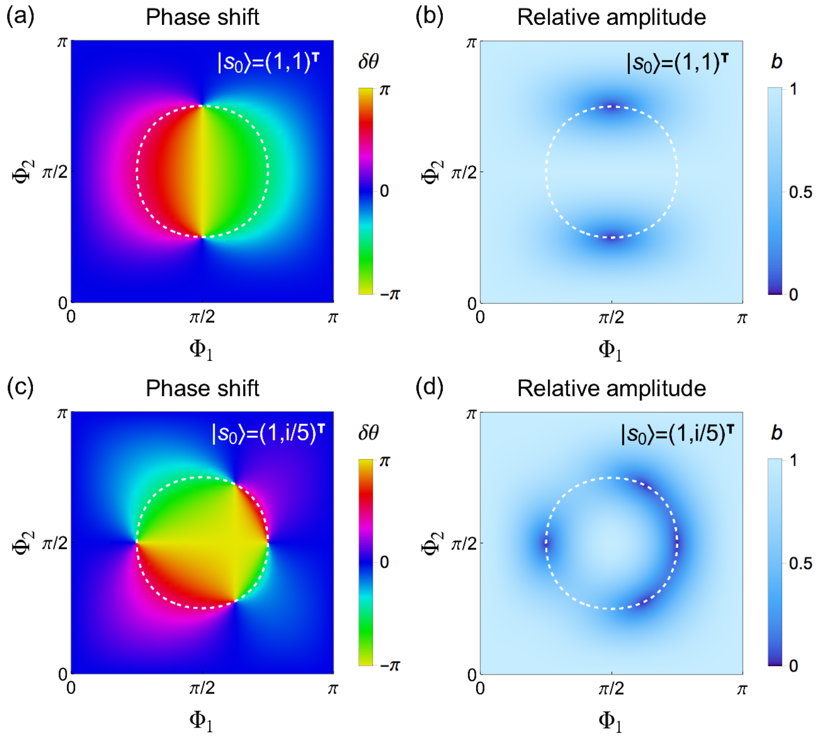

Supplementary Figure 8. Phase shift $\delta\theta$ and relative amplitude b of the intensity interference fringes varying with the vortex fluxes Φ_1, Φ_2 for two incident spinors: (a,b) $|s_0\rangle = (1,1)^\top$, and (c,d) $|s_0\rangle = (1, i/5)^\top$. On the white dashed circle, the Wilson loop of the closed path $c_0 = \gamma_{\rm I} \circ \gamma_{\rm II}^{-1}$ equals zero, $W(c_0) = 0$. For a fixed $|s_0\rangle$, there exist singularities of the phase shift $\delta\theta$ lying on the white circle, and the relative amplitude is reduced to zero, i.e. $b = 0$, at such singularities.

In particular, when $\sin^2\Phi_1 \sin^2\Phi_2 = 1/2$, the phase shift converges to two discrete numbers: $\delta\theta = \pm\pi/2$, and the relative amplitude is reduced to $b(\Phi_1,\Phi_2) = \sqrt{2}|\vec{s}_0 \cdot \vec{u}(\Phi_1,\Phi_2)|$, and the Wilson loop is reduced to zero: $W(c_0) = b\cos\delta\theta = 0$. Furthermore, if $\sin^2\Phi_1 \sin^2\Phi_2 = 1/2$ and $\vec{s}_0 \cdot \vec{u} = 0$ are achieved simultaneously, the relative amplitude is reduced to zero $b = 0$, as a result, the spatial fluctuation of the quasi-intensity $|\psi|^2$ vanishes. According to Eq. (25) in the main text, $b = 0$ implies that the finial spinors of the two beams are orthogonal: $\langle\psi_{\rm I}(y)|\psi_{\rm II}(y)\rangle = a(y)^2\langle s_{\rm I}|s_{\rm II}\rangle = 0$, and hence their superposition leads to no intensity fluctuation but a pure spin rotation around the axis $\Delta\vec{s} = 2\vec{s}_{\rm I}$ on the screen.

Supplementary Figure 8 shows the non-Abelian-honolomy induced phase shift $\delta\theta$ and relative amplitude b as functions of Φ_1 and Φ_2 for two fixed incident spinors. With the initial spinor $|s_0\rangle = (1,1)^\top$, we observe, in Supplementary Figure 8(a), two singularities at $\{\Phi_1,\Phi_2\} = \{\pi/2, \pi/4\}$ and $\{\Phi_1,\Phi_2\} = \{\pi/2, 3\pi/4\}$, where the phase shift $\delta\theta(\Phi_1,\Phi_2)$ is ill-defined. Meanwhile, the interference amplitude b drops to zero at the singularities, as shown in Supplementary Figure 8(b). This result is supported by examining the spinors of the two beams, which evolve eventually into the pair of orthogonal states $|\uparrow\rangle = (1,0)^\top$, $|\downarrow\rangle = (0,1)^\top$ for both singularities. Similarly, with the incident spinor $|s_0\rangle = (1, i/5)^\top$, there are four singularities of $\delta\theta(\Phi_1,\Phi_2)$ corresponding to the zero points of $b(\Phi_1,\Phi_2)$ as shown in Supplementary Figure 8 (c,d). All of such singularities of $\delta\theta(\Phi_1,\Phi_2)$ lie on the curve of $\sin^2\Phi_1\sin^2\Phi_2 = 1/2$ for any initial spin $\vec{s}_0$.

2. Spin projected interference

Apart from directly observing the total intensity $|\psi|^2(y)$ on the screen discussed in the main text, the nontrivial interference effect can alternatively be detected via measuring the projected intensity onto a certain spin direction with a spin filter. For a certain spin direction $\vec{n}$, the corresponding projection operator reads $|n\rangle\langle n| = \frac{1}{2}(\mathbb{I} + \vec{n}\cdot\vec{\sigma})$. The

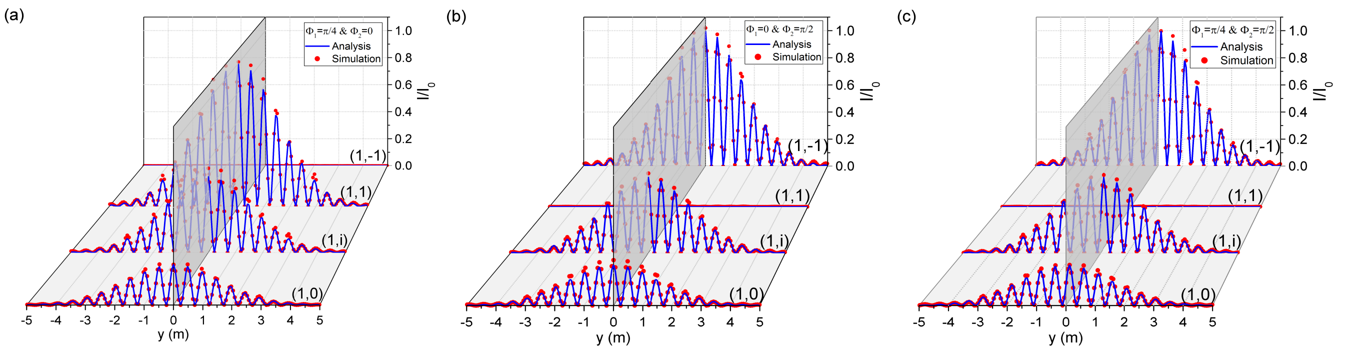

Supplementary Figure 9. Spin projected interference patterns for the non-Abelian AB system shown in Fig.2 of the main text with the vortex fluxes (a) $\{\Phi_1, \Phi_2\} = \{\pi/4, 0\}$, (b) $\{\Phi_1, \Phi_2\} = \{0, \pi/2\}$, and (c) $\{\Phi_1, \Phi_2\} = \{\pi/4, \pi/2\}$. In the three cases, the incident spinor is given by $|s_0\rangle = (1,1)^\top$, the spin-filter states $|n\rangle$ for the projection are selected as $(1,0)^\top$, $(1,i)^\top$, $(1,1)^\top$ and $(1,-1)^\top$. The red dotes and the blue lines correspond to the full-wave simulation and the theoretical results using Eq. (60) respectively. The grey reference planes located at y=0 marks the original position of the central peak without phase shift. In (a,b), the central peak is coincident with the grey plane, thus there is no phase shift $\delta\theta = 0$, while in (c) the phase shift is $\delta\theta = \pi/2$.

projected intensity on this spin direction is given by

$$\begin{aligned} |\langle n\,|\,\psi(y)\rangle|^2 &= \langle\psi(y)\,|n\rangle\langle n|\,\psi(y)\rangle = \left\langle\psi(y)\left|\frac{1}{2}\left(\mathbb{I}+\vec{n}\cdot\vec{\sigma}\right)\right|\psi(y)\right\rangle \\ &= \frac{1}{2}|\psi|^2(y)\Big(1+\vec{n}\cdot\vec{s}(y)\Big), \end{aligned} \tag{60}$$

where $|\psi\rangle = |\psi_{\rm I}\rangle + |\psi_{\rm II}\rangle$, and $\vec{s} = \langle\psi|\vec{\sigma}|\psi\rangle/|\psi|^2$. In general, a projected interference pattern can exhibit the nontrivial information of the intensity fringes $|\psi|^2(y)$ and the spin fluctuation $\vec{s}(y)$ simultaneously. If $\vec{n}$ is parallel to $\Delta\vec{s}$ (see Eq. (55), we have $\vec{n}\cdot\vec{s}(y) \equiv 0$, and hence the projected interference pattern would be in the same shape as the total intensity $|\psi|^2(y)$.

We carry out the numerical simulation of the interference in our designed non-Abelian AB system where the finial states of the two beams $\gamma_{\rm I}$, $\gamma_{\rm II}$ are projected onto certain spin directions on the detection plane. First, we study the situation of a single vortex by setting either $\Phi_1 = 0$ or $\Phi_2 = 0$. In this case, the system is reduced to being essentially Abelian. Because of the same AB phase factor for the two beams, the superposed spin density $|\psi|^2(y)\vec{s}$ are uniformly oriented on the screen, and there is no phase shift ($\delta\theta = 0$) and amplitude contraction ($b = 1$). Thus, the projected interference onto any direction $\vec{n}$ is consistent with the total intensity up to a scaling parameter $(1+\vec{n}\cdot\vec{s})/2$. In Supplementary Figure 9(a), the projected interference patterns onto 4 different directions are plotted, where only the $\hat{\sigma}_1$ vortex exists with the flux $\Phi_1 = \pi/4$ and the incident spinor reads $|s_0\rangle = (1,1)^\top$. As $|s_0\rangle$ is an eigenstate of $\hat{\sigma}_1$, the spin is conserved during propagation. Accordingly, the projection onto $(1,1)^\top$ are identical to the total intensity $|\psi|^2(y)$, while the projection onto $(1,-1)^\top$ vanishes as shown in Supplementary Figure 9(a), and the projected intensities onto $(1,0)^\top$ and $(1,i)^\top$ are exactly halved. Supplementary Figure 9(b) shows the projected interference patterns for a single $\hat{\sigma}_2$ vortex carrying the flux $\Phi_2 = \pi/2$. As the incident spinor $|s_0\rangle = (1,1)^\top$ is not an eigenstate of $\hat{\sigma}_2$, the spins of the two beams rotate along the trajectories and finally convert to $|s_{\rm I}\rangle = |s_{\rm II}\rangle = (1,-1)^\top$, evidenced by the vanishing projection onto $(1,1)^\top$.

In Supplementary Figure 9(c), we illustrate the projected interference patterns in a genuine non-Abelian AB system with two noncommutative vortices. And the specific fluxes $\{\Phi_1, \Phi_2\} = \{\pi/4, \pi/2\}$ indicate a zero Wilson loop $W(c_0) = 0$. And since $\vec{u} = \sqrt{2}/2\vec{e}_1$, $\vec{s}_0 = \vec{e}_1$ satisfy $\vec{u}\times\vec{s}_0 - 2\vec{u}\cdot(\vec{s}_0\times\vec{e}_3)\vec{e}_3 = 0$, the finial spins of the two beams fall into a uniform direction $\vec{s}_{\rm I} = \vec{s}_{\rm II} = -\vec{e}_1$, leading to the vanishing projection onto $(1,1)^\top$. Therefore, the projected intensity on any direction $\vec{n}$ is always proportional to the total intensity: $|\langle n|\psi(y)\rangle|^2 = \frac{1}{2}|\psi(y)|^2(1-\vec{n}\cdot\vec{e}_1)$. Meanwhile, the phase shift and relative amplitude for all the projected interference patterns are fixed as $\delta\theta = \pi/2$, $b = \sqrt{2}|\vec{s}_0\cdot\vec{u}| = 1$, which are confirmed by the numerical results in Supplementary Figure 9(c).

Supplementary Note 8. Designing non-Abelian AB system with gyroelectric materials

Although the genuine non-Abelian AB system designed in the main text has a rather simple geometry, the background materials is required to support both gyroelectric and gyromagnetic responses, meanwhile, their electric and magnetic gyrotation vectors should obey a rigorous relation. In this section, we offer an alternative design of the non-Abelian AB system using gyroelectric materials without gyromagnetic response which would be more easily realized in practice.

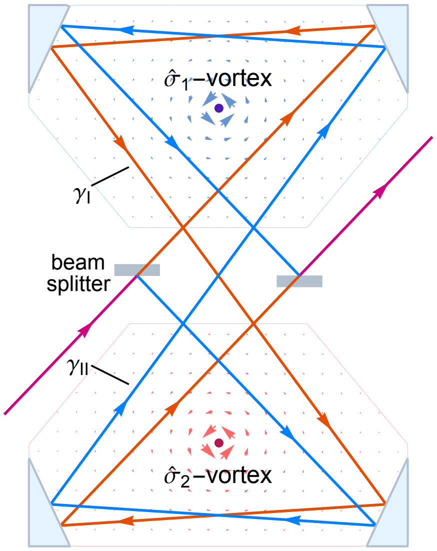

Supplementary Figure 10. Alternative design of the non-Abelian AB system with two interfering optical paths $\gamma_{\rm I}$, $\gamma_{\rm II}$, where the background light blue (red) arrows denote the $\hat{\sigma}_1$ ($\hat{\sigma}_2$) component $\mathcal{A}^1$ ($\mathcal{A}^2$) of the synthetic non-Abelian vector potential. The media used to imitate $\hat{\sigma}_1$-vortex and $\hat{\sigma}_1$-vortex in the upper and lower half-spaces are, respectively, an inhomogeneous reciprocal anisotropic material and an inhomogeneous gyroelectric material.

As shown in Supplementary Figure 10, the lower half-plane contains a synthetic non-Abelian vortices made up of gyroelectric materials:

$$\overleftrightarrow{\varepsilon}/\varepsilon_0 = \left(\begin{array}{c|c} \varepsilon_T \overleftrightarrow{I}_{2\times2} & i\,\mathbf{g}_1 \\ \hline -i\,\mathbf{g}_1 & \varepsilon_z \end{array}\right), \qquad \overleftrightarrow{\mu}/\mu_0 = \left(\begin{array}{c|c} \mu_T \overleftrightarrow{I}_{2\times2} & 0 \\ \hline 0 & \mu_z \end{array}\right), \tag{61}$$

where $\varepsilon_T = \alpha\,\mu_T$ ($\alpha > 0$) is supposed to be homogeneous, while $\mathbf{g}_1$, ε_z, and μ_z are real-valued functions of coordinates x, y. By rescaling the vacuum permittivity $\varepsilon_0' = \alpha\,\varepsilon_0$, we obtain the synthetic vector and scalar gauge potentials:

$$\hat{\mathcal{A}} = k_0 \frac{\mathbf{g}_1 \times \mathbf{e}_z}{2\sqrt{\alpha}}\,\hat{\sigma}_2, \quad \hat{\mathcal{A}}_0 = \frac{k_0}{2\sqrt{\alpha}}\mathbf{e}_z\cdot(\nabla\times\mathbf{g}_1)\,\hat{\sigma}_1 + \frac{{k_0}^2}{2}\left(\varepsilon_z - \alpha\mu_z - \frac{|\mathbf{g}_1|^2}{\alpha}\right)\hat{\sigma}_3, \quad V_0 = \frac{k_0^2}{2}\left[\frac{|\mathbf{g}_1|^2}{2\alpha} - \varepsilon_z - \alpha\mu_z\right]. \tag{62}$$

Since $\mathbf{g} = \mathbf{g}_1 \times \mathbf{e}_z$ is precisely the gyration vector of the gyroelectric materials, $\mathcal{A}_2$ is parallel to the gyration vector everywhere. In order to eliminate the synthetic non-Abelian magnetic fields $\hat{\mathcal{B}} = \nabla \times \hat{\mathcal{A}}_2\hat{\sigma}_2 = 0$ in the whole medium (except for a small domain which is simplified as a singularity), we let $\mathcal{A}_2$ be an irrotational vortex with the center at $-\mathbf{r}_0 = -r_0\mathbf{e}_y$:

$$\mathcal{A}_2 = \frac{\Phi_2}{2\pi}\nabla\arctan\left(\frac{x}{y+r_0}\right) = \frac{\Phi_2}{2\pi|\mathbf{r}+\mathbf{r}_0|^2}\left[-(y+r_0)\mathbf{e}_x + x\mathbf{e}_y\right]. \tag{63}$$

As such, the off-diagonal term of $\overleftrightarrow{\varepsilon}$ is given by

$$\mathbf{g}_1 = \frac{2\sqrt{\alpha}}{k_0}\,\mathbf{e}_z \times \hat{\mathcal{A}}_2 = -\frac{\Phi_2\sqrt{\alpha}}{\pi k_0|\mathbf{r}+\mathbf{r}_0|^2}\left[x\mathbf{e}_x + (y+r_0)\mathbf{e}_y\right]. \tag{64}$$

In order to extinguish non-Abelian and Abelian electric fields $\hat{\mathcal{E}} = \nabla\hat{\mathcal{A}}_0 + i[\hat{\mathcal{A}}_0, \hat{\mathcal{A}}]$ and $\mathsf{E} = -\nabla V_0$, the scalar potentials should satisfy $\hat{\mathcal{A}}_0 = 0$ and $V_0 = \text{const.}$. The benefit of adopting the irrotational vortex to form $\mathcal{A}_2$ is that $\mathcal{A}_0^1 \propto \mathbf{e}_z \cdot (\nabla \times \mathbf{g}_1) = 0$ can be simultaneously satisfied. Therefore, the requirements to the scalar potentials can be met providing that the z-components of permittivity and permeability satisfy

$$\varepsilon_z = \frac{1}{2}\left(\frac{|\mathbf{g}_1|^2}{\alpha} + \text{const.}\right) = \frac{1}{2}\left(\frac{\Phi_2^2}{\pi^2 k_0^2}|\mathbf{r}+\mathbf{r}_0|^{-2} + \text{const.}\right), \tag{65a}$$

$$\mu_z = \frac{1}{2\alpha}\left(-\frac{|\mathbf{g}_1|^2}{\alpha} + \text{const.}\right) = \frac{1}{2\alpha}\left(-\frac{\Phi_2^2}{\pi^2 k_0^2}|\mathbf{r}+\mathbf{r}_0|^{-2} + \text{const.}\right). \tag{65b}$$

To realize this kind of gyroelectric materials, a promising approach is to design suitable gyroelectric metamaterials with either passive magneto-optic composites [31] or active structures [32] which have the advantages of strong gyrotropic response and turnable anisotropy. In particular, if the gyroelectricity is induced by magneto-optic effect, the gyration vector $\mathbf{g}$ should be parallel and proportional to the external magnetic field. For the present case, if a line current alone z-axis is placed at the $-\mathbf{r}_0$, the generated magnetic field forms an irrotational vortex, and the induced gyration vector gives exactly the $\hat{\sigma}_2$-vortex.

In the upper half-plane, the synthetic $\hat{\sigma}_1$-vortex with the center at $\mathbf{r}_0 = r_0\mathbf{e}_y$ can be constructed by reciprocal anisotropic materials

$$\overleftrightarrow{\varepsilon}/\varepsilon_0 = \left(\begin{array}{c|c} \varepsilon_T \overleftrightarrow{I}_{2\times 2} & \mathbf{g}_1' \\ \hline \mathbf{g}_1' & \varepsilon_z \end{array}\right), \qquad \overleftrightarrow{\mu}/\mu_0 = \left(\begin{array}{c|c} \mu_T \overleftrightarrow{I}_{2\times 2} & 0 \\ \hline 0 & \mu_z \end{array}\right), \tag{66}$$

with $\varepsilon_T = \alpha\,\mu_T$ ($\alpha > 0$) being homogeneous and $\mathbf{g}_1'$, ε_z, μ_z being real-valued functions of x, y. Similarly to the lower half-space, we let $\hat{\mathcal{A}} = \mathcal{A}_1\hat{\sigma}_1$ form an irrotational vortex in the anisotropic material:

$$\mathcal{A}_1 = \frac{\Phi_1}{2\pi}\nabla\arctan\left(\frac{x}{y-r_0}\right) = \frac{\Phi_1}{2\pi|\mathbf{r}-\mathbf{r}_0|^2}\left[-(y-r_0)\mathbf{e}_x + x\mathbf{e}_y\right], \tag{67}$$

and let $\hat{\mathcal{A}}_0 = 0$ and $V_0 = \text{const.}$. Then the material parameters are obtained as follows

$$\mathbf{g}_1' = -\frac{\Phi_1\sqrt{\alpha}}{\pi k_0|\mathbf{r}-\mathbf{r}_0|^2}\left[x\mathbf{e}_x + (y-r_0)\mathbf{e}_y\right], \tag{68a}$$

$$\varepsilon_z = \frac{1}{2}\left(\frac{\Phi_1^2}{\pi^2 k_0^2}|\mathbf{r}-\mathbf{r}_0|^{-2} + \text{const.}\right), \tag{68b}$$

$$\mu_z = \frac{1}{2\alpha}\left(-\frac{\Phi_1^2}{\pi^2 k_0^2}|\mathbf{r}-\mathbf{r}_0|^{-2} + \text{const.}\right). \tag{68c}$$

In comparison with the design in the main text, a disadvantage of the reduced non-Abelian AB system is that the sudden change of the synthetic gauge potentials will induce nonzero non-Abelian gauge flux at the boundaries of the materials. In order to prevent the boundary flux from affecting the total flux enclosed by the optical paths, the optical paths encircling the non-Abelian vortices should form closed loops before traversing the boundaries of the upper or lower pieces of materials. Thus we designed a new optical path diagram shown in Supplementary Figure **10**. The new AB system can give rise to the identical interfering results as the original non-Abelian system in the main text, namely the optical beams passing through the two paths γ_{I} and γ_{II} can gain the non-Abelian phase factors $\hat{U}_{\gamma_{\mathrm{I}}} = \hat{U}_2^{-1}\hat{U}_1$ and $\hat{U}_{\gamma_{\mathrm{II}}} = \hat{U}_1\hat{U}_2^{-1}$ respectively, where $\hat{U}_i = \exp\left[i\Phi_i\hat{\sigma}_i\right]$ ($i = 1, 2$). Since $\hat{U}_1$ and $\hat{U}_2$ do not commute with each other, the opposite sequences of winding around the two vortices lead to different non-Abelian phase factors $\hat{U}_{\gamma_{\mathrm{I}}} \neq \hat{U}_{\gamma_{\mathrm{II}}}$.

Supplementary References

[1] Liu, F. & Li, J. Gauge field optics with anisotropic media. *Phys. Rev. lett.* **114**, 103902 (2015).

[2] Liu, F., Wang, S., Xiao, S., Hang, Z. H. & Li, J. Polarization-dependent optics using gauge-field metamaterials. *Appl. Phys. Lett.* **107**, 241106 (2015).

[3] Fang, K. & Fan, S. Effective magnetic field for photons based on the magneto-optical effect. *Phys. Rev. A* **88**, 043847 (2013).

[4] Jacobs, D. A., Miroshnichenko, A. E., Kivshar, Y. S. & Khanikaev, A. B. Photonic topological Chern insulators based on Tellegen metacrystals. *New J. Phys.* **17**, 125015 (2015).

[5] Cook, R. J., Fearn, H. & Milonni, P. W. Fizeaus experiment and the Aharonov–Bohm effect. *Am. J. Phys.* **63**, 705–710 (1995).

[6] Leonhardt, U. & Piwnicki, P. Optics of nonuniformly moving media. *Phys. Rev. A* **60**, 4301 (1999).

[7] Sawada, K. & Nagaosa, N. Optical magnetoelectric effect in multiferroic materials: Evidence for a Lorentz force acting on a ray of light. *Phys. Rev. Lett.* **95**, 237402 (2005).

[8] Zawadzki, W. & Rusin, T. M. Zitterbewegung (trembling motion) of electrons in semiconductors: a review. *J. Phys.: Condens. Matter* **23**, 143201 (2011).

[9] Onoda, M., Murakami, S. & Nagaosa, N. Geometrical aspects in optical wave-packet dynamics. *Phys. Rev. E* **74**, 066610 (2006).

[10] Bliokh, K. Y., Frolov, D. Y. & Kravtsov, Y. A. Non-Abelian evolution of electromagnetic waves in a weakly anisotropic inhomogeneous medium. *Phys. Rev. A* **75**, 053821 (2007).

[11] Bliokh, K. Y., Niv, A., Kleiner, V. & Hasman, E. Geometrodynamics of spinning light. *Nat. Photon.* **2**, 748–753 (2008).

[12] Aharonov, Y. & Bohm, D. Significance of electromagnetic potentials in the quantum theory. *Phys. Rev.* **115**, 485 (1959).

[13] Chruściński, D. & Jamiołkowski, A. *Geometric phases in classical and quantum mechanics* (Birkhuser Basel, 2004).

[14] Wu, T. T. & Yang, C. N. Concept of nonintegrable phase factors and global formulation of gauge fields. *Phys. Rev. D* **12**, 3845 (1975).

[15] Aharonov, Y. & Casher, A. Topological quantum effects for neutral particles. *Phys. Rev. Lett.* **53**, 319 (1984).

[16] Anandan, J. Electromagnetic effects in the quantum interference of dipoles. *Phys. Lett. A* **138**, 347–352 (1989).

[17] Oh, S., Ryu, C.-M. & Salk, S.-H. S. Equivalence between Aharonov-Bohm and Aharonov-Casher effects, and motive forces. *Phys. Rev. A* **50**, 5320 (1994).

[18] Horváthy, P. Non-Abelian Aharonov-Bohm effect. *Phys. Rev. D* **33**, 407 (1986).

[19] Sundrum, R. & Tassie, L. J. Non-Abelian Aharonov–Bohm effects, Feynman paths, and topology. *J. Math. Phys.* **27**, 1566–1570 (1986).

[20] Goldman, N., Juzeliūnas, G., Öhberg, P. & Spielman, I. B. Light-induced gauge fields for ultracold atoms. *Rep. Prog. Phys.* **77**, 126401 (2014).

[21] Wilczek, F. & Wu, Y.-S. Space-time approach to holonomy scattering. *Phys. Rev. Lett.* **65**, 13 (1990).

[22] Bucher, M. The Aharonov-Bohm effect and exotic statistics for non-Abelian vortices. *Nucl. Phys. B* **350**, 163–178 (1991).

[23] Lo, H.-K. & Preskill, J. Non-Abelian vortices and non-Abelian statistics. *Phys. Rev. D* **48**, 4821 (1993).

[24] Nayak, C., Simon, S. H., Stern, A., Freedman, M. & Sarma, S. D. Non-Abelian anyons and topological quantum computation. *Rev. Mod. Phys.* **80**, 1083 (2008).

[25] Rotman, J. J. *An introduction to algebraic topology* (Springer, 2013).

[26] Kobayashi, S. & Nomizu, K. *Foundations of differential geometry*, vol. 1 (Interscience Publishers, 1963).

[27] Oh, C. H., Soo, C. P. & Lai, C. H. The propagator in the generalized Aharonov–Bohm effect. *J. Math. Phys.* **29**, 1154–1157 (1988).

[28] Aref'eva, I. Y. Non-Abelian Stokes formula. *Theor. Math. Phys.* **43**, 353–356 (1980).

[29] Fishbane, P. M., Gasiorowicz, S. & Kaus, P. Stokes's theorems for non-Abelian fields. *Phys. Rev. D* **24**, 2324 (1981).

[30] Broda, B. Non-Abelian Stokes theorem. In *Modern Nonlinear Optics, Part 2*, vol. 119, 429–468 (Wiley, 2002), 2 edn.

[31] Sadatgol, M., Rahman, M., Forati, E., Levy, M. & Güney, D. Ö. Enhanced faraday rotation in hybrid magneto-optical metamaterial structure of bismuth-substituted-iron-garnet with embedded-gold-wires. *J. Appl. Phys.* **119**, 103105 (2016).

[32] Wang, Z. *et al.* Gyrotropic response in the absence of a bias field. *Proc. Natl. Acad. Sci.* **109**, 13194–13197 (2012).
